# Noncanonical transcription initiation is primarily tissue specific and epigenetically tuned in paleopolyploid plants

**DOI:** 10.1093/plcell/koae288

**Published:** 2024-11-14

**Authors:** Xutong Wang, Jingbo Duan, Chancelor B Clark, Wanjie Feng, Jianxin Ma

**Affiliations:** Department of Agronomy, Purdue University, West Lafayette, IN 47907, USA; Center for Plant Biology, Purdue University, West Lafayette, IN 47907, USA; National Key Laboratory of Crop Genetic Improvement, College of Plant Science and Technology, Hubei Hongshan Laboratory, Huazhong Agricultural University, Wuhan, Hubei 430070, China; Department of Agronomy, Purdue University, West Lafayette, IN 47907, USA; Center for Plant Biology, Purdue University, West Lafayette, IN 47907, USA; Department of Agronomy, Purdue University, West Lafayette, IN 47907, USA; Center for Plant Biology, Purdue University, West Lafayette, IN 47907, USA; National Key Laboratory of Crop Genetic Improvement, College of Plant Science and Technology, Hubei Hongshan Laboratory, Huazhong Agricultural University, Wuhan, Hubei 430070, China; Department of Agronomy, Purdue University, West Lafayette, IN 47907, USA; Center for Plant Biology, Purdue University, West Lafayette, IN 47907, USA

## Abstract

Alternative transcription initiation (ATI) appears to be a ubiquitous regulatory mechanism of gene expression in eukaryotes. However, the extent to which it affects the products of gene expression and how it evolves and is regulated remain unknown. Here, we report genome-wide identification and analysis of transcription start sites (TSSs) in various soybean (*Glycine max*) tissues using a survey of transcription initiation at promoter elements with high-throughput sequencing (STRIPE-seq). We defined 193,579 TSS clusters/regions (TSRs) in 37,911 annotated genes, with 56.5% located in canonical regulatory regions and 43.5% from start codons to 3′ untranslated regions, which were responsible for changes in open reading frames of 24,131 genes. Strikingly, 6,845 genes underwent ATI within coding sequences (CDSs). These CDS-TSRs were tissue-specific, did not have TATA-boxes typical of canonical promoters, and were embedded in nucleosome-free regions flanked by nucleosomes with enhanced levels of histone marks potentially associated with intragenic transcriptional initiation, suggesting that ATI within CDSs was epigenetically tuned and associated with tissue-specific functions. Overall, duplicated genes possessed more TSRs, exhibited lower degrees of tissue specificity, and underwent stronger purifying selection than singletons. This study highlights the significance of ATI and the genomic and epigenomic factors shaping the distribution of ATI in CDSs in a paleopolyploid eukaryote.

## Teaser

Widespread transcription initiation within the coding sequences of genes is associated with tissue specificity and is marked by chromatin accessibility in the soybean genome.

**IN A NUTSHELL**

**Background:** Transcriptional regulation is essential for biological processes like development and environmental response. Accurate identification of transcription start sites (TSSs) is critical for understanding gene expression. While canonical TSSs are generally located in promoter regions, non-canonical TSSs are also observed, indicating complex regulatory mechanisms. Traditional TSS identification methods are costly and labor-intensive. STRIPE-seq, a recent, efficient technique for genome-wide TSS mapping, offers a new approach. As a polyploid crop with multiple whole-genome duplication events, soybean serves as an ideal model to study transcriptional patterns.

**Question:** The impact of transcription initiation on gene expression, evolution, and regulation in soybean remains only partially understood. This study uses STRIPE-seq to profile transcription start regions (TSRs) in soybean and explores tissue-specific transcription and evolutionary dynamics. By integrating other available omics datasets, we aim to identify epigenetic features associated with these TSRs and their potential role in diversifying gene function across tissues.

**Findings:** The study identified extensive noncanonical TSRs within coding regions across multiple soybean tissues, revealing a new layer of transcriptional complexity. TSRs mapped to numerous genes, with a large portion in coding regions that lack typical promoter elements like TATA-boxes and display strong tissue specificity. Genes with multiple TSRs had higher expression levels and underwent stronger purifying selection, underscoring functional significance. Tissue-specific TSRs within coding regions likely enhance protein diversification and enable tissue-specific gene functions. These TSRs exhibit distinct histone modifications, suggesting regulated noncanonical initiation that may support functional specialization in the polyploid soybean genome.

**Next steps:** This work raises key questions about transcription regulation in soybean. Future research will examine (i) mechanisms underlying tissue-specific alternative transcription initiation (ATI) in coding regions, (ii) functional impacts of truncated proteins from ATI, (iii) the sequence basis for transcription initiation in soybean, and (iv) how transcription initiation variation in soybean populations has evolved and is linked to traits important for domestication and crop improvement.

## Introduction

Transcriptional regulation is crucial for various biological processes such as development, tissue differentiation, and environmental responses (Singh 1998; Macrae and Long 2012). Understanding of the fundamental aspects of transcription relies on the accurate identification of transcription start sites (TSSs), which are typically hallmarked with cis-regulatory DNA elements, TATA-boxes, in various eukaryotic genomes (Davuluri et al. 2008; Mejía-Guerra et al. 2015; Policastro et al. 2020; Thieffry et al. 2020). The TSSs are core parts of promoters and often clustered as transcription start regions (TSRs) (Policastro et al. 2020). Canonical TSRs are typically located in the core promoter regions of genes, but noncanonical TSRs besides those regions were also observed. Despite the availability of genome sequences and annotations for numerous eukaryotic organisms, TSRs in most of these organisms remain to be identified for an in-depth and comprehensive understanding of the regulatory mechanisms of transcription and the functional consequences.

Over the past few decades, several methods, including cap analysis of gene expression (CAGE) (Murata et al. 2014), nano-CAGE (Batut and Gingeras 2013), paired-end analysis of TSSs (Shahmuradov et al. 2017), and RNA annotation and mapping of promoters for the analysis of gene expression (Ni et al. 2010; Batut and Gingeras 2013; Napoli 2017), have been developed and extensively used to identify TSRs in eukaryotes. Each of these methods has its own advantages and disadvantages, but the major limitations are the cost and labor-intensive nature (Shahmuradov et al. 2017; de Medeiros Oliveira et al. 2021). Recently, a fast, efficient, and simple protocol, called survey of transcription initiation at promoter elements with high-throughput sequencing (STRIPE-seq), was developed and first used for genome-wide identification of the capped 5′ ends of transcripts in yeast and humans (Policastro et al. 2020). The STRIPE-seq library construction involves 3 enzymatic steps to deplete uncapped RNA fragments. Initially, uncapped RNA fragments are eliminated through digestion with terminator exonuclease (TEX). This is followed by a template-switching reverse transcription (TSRT) step, utilizing a barcoded reverse transcription oligo (RTO) with a random pentamer primer. Subsequently, a unique molecular identifier (UMI)-containing, 5′-biotin-modified template-switching oligo (TSO) is introduced, featuring 3 3′ riboguanosines that facilitate its annealing to the untemplated triplet Cs generated by reverse transcriptase upon reaching the m7G cap. The final step involves a single round of PCR amplification using the purified TSRT product as a template, ensuring the incorporation of TruSeq adapters on both ends of the insert. This refined protocol effectively selects capped transcripts while minimizing bias and maintaining the integrity of the original RNA population. STRIPE-seq enables the identification of TSRs and quantification of gene expression levels simultaneously, representing an additional advantage over other methods.

Soybean (*Glycine max* [L.] Merr.) is a primary source of protein for livestock feed as well as a valuable contributor to vegetable protein, oil, and various industrial and bioenergy compounds (Hartman et al. 2011). As such, soybean is among the few crops whose reference genomes were assembled over a decade ago (Schmutz et al. 2010). One of the unique features of the soybean genome is that it has experienced 2 recent rounds of whole-genome duplication (WGD) events that occurred ∼59 and ∼13 million years ago (MYA), respectively, followed by the loss of a large proportion of the duplicated genes (Schmutz et al. 2010; Du et al. 2012; Zhao et al. 2017). On the other hand, although approximately two-thirds of the duplicated genes formed through the more recent WGD event are retained in the current soybean genome, the majority of the duplicated genes showed distinct expression patterns regarding transcript abundance and tissue specificity, probably reflecting their subfunctionalization and/or neofunctionalization (Zhao et al. 2017). Therefore, soybean is an ideal system to understand how the transcription patterns, such as the distribution of TSSs of duplicated genes, have been shaped by WGD and subsequent subgenome differentiation.

The annotation of putative TSSs in the soybean reference genome was primarily based on predictions, lacking experimental validation. In this study, STRIPE-seq was employed to identify and validate TSRs in the soybean reference genome, heralding the inaugural application of this technology in plants. By profiling TSRs across the entire genome in 5 vegetative tissues and 3 reproductive tissues, we identified alternative transcription initiations (ATIs) in the context of tissue specification, subgenome fractionation, and epigenomic modification. This analysis revealed the evolutionary factors and their interplay driving transcriptional and functional innovation of plant genes in a paleopolyploid genome.

## Results

### STRIPE-seq identified genome-wide TSRs in soybean

**Figure 1.**
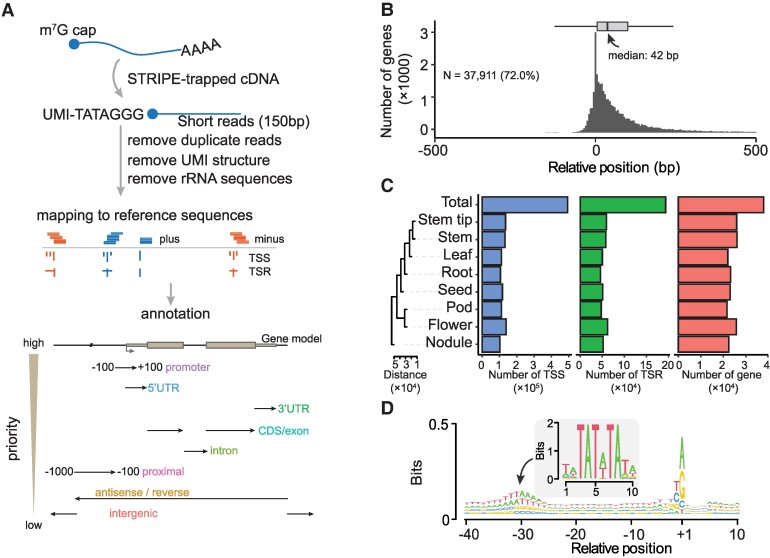
Annotation and evaluation of TSRs detected by survey of transcription initiation at promoter elements with STRIPE-seq. **A)** Workflow of processing STRIPE-seq reads and hierarchical annotation strategy and schematic representation of the annotation of TSRs to the reference genome. The priority annotation levels, ranked from high to low, are displayed on the left side of the bottom panel. **B)** The distribution of TSRs compared with annotated TSSs (Williams 82 version 2). The boxplot at the top illustrates the distribution of distances between annotated TSSs and the detected TSRs. In each box plot, borders represent the first and third quartiles, center line denotes median, and whiskers extend to 1.5 times the IQR beyond the quartiles. **C)** Statistics are provided for the numbers of TSSs, TSRs, and genes covered in 8 tissues and the total. The cladogram on the left shows the distances among tissues, calculated using the Euclidean metric based on the relative abundance of TSRs. For each tissue, we only have 1 replicate for quantification. **D)** Sequence patterns around all unique TSSs. The TSS was defined as the peak site of the TSR. The motif in the central panel represents the TATA-box calculated in soybean.

We identified TSRs in 8 soybean tissues, including leaves, stems, stem tips, roots, nodules, flowers, pods, and developing seeds, using the STRIPE-seq protocol with slight modification, which involved incorporating the RiboMinus Plant Kit (Invitrogen) for rRNA depletion from total RNAs isolated from each of the 8 tissues (Policastro et al. 2020). This modification was important because the TEX included in STRIPE is less effective than RiboMinus at removing rhizobial rRNAs from nodule tissue, which contains prokaryotic cells (Čuklina et al. 2016). The STRIPE-seq of the 8 issues generated 646 million reads (Supplementary Table S1). On average, 92% of the reads from each tissue contained a unique UMI, indicating that the vast majority of reads started from 5′ caps of mRNAs, and thus our STRIPE-seq experiment was of high quality (Supplementary Table S1). To quantify TSS abundances and gene expression levels as accurately as possible, we removed redundant reads from PCR amplification involved in library construction based on the UMIs (Fig. 1A), and then conducted saturation analysis at different levels of read coverage (3, 6, and 9 reads) defining a particular TSS in each tissue based on nonredundant reads with distinct UMIs. The saturation analysis revealed that the numbers of genes with detected TSSs at each level reached a plateau in all tissues, suggesting that the sequence depth was sufficient for the intended analyses (Supplementary Fig. S1). An TSS was defined when it was supported by >1 nonredundant transcripts per million (TPM) in at least 1 tissue, corresponding to an average unique read count of 7.3 per tissue. This cutoff would effectively exclude “fake” TSSs resulted from degraded RNAs from genuine ones defined by reads staring from the capped 5′ ends. The majority of TSSs detected in gene bodies were found to be distributed within 42 bp downstream of previously annotated TSSs in the soybean (cv. Williams 82) reference genome (Schmutz et al. 2010; soybase.org; Fig. 1B; Supplementary Fig. S1), demonstrating the effectiveness of the STRIPE-seq method in capturing TSSs.

A total of 492,858 unique TSSs were detected by STRIPE-seq in the WM82.a2 reference genome (soybase.org). These TSSs defined 193,579 TSRs of 37,911 protein-coding genes, accounting for approximately two-thirds of all protein-coding genes annotated in the entire genome (Fig. 1C). According to this TSS dataset, only a small percentage (2.81%) of annotated genes in reference genome have correctly annotated TSSs, exemplifying an urgent need for experiment-based identification and annotation of TSSs in plants. The TSR numbers detected in our study varied considerably among the 8 tissues, ranging from 45,943 in roots to 62,214 in flowers, with an average of 53,354 per tissue. Likewise, the average numbers of genes with defined TSRs range from 21,998 in leaves to 25,806 in flowers, with an average of 23,760 per tissue, and noticeably, the nodule tissue exhibited the most unique TSRs compared with other tissues (Fig. 1C). To evaluate the accuracy of TSRs defined by STRIPE-seq, we compared all the TSSs identified in this study with those annotated in the reference genome, those suggested by de novo RNA-seq transcripts, and those defined by full-length RNA-seq (Li et al. 2021). Only the TSRs defined by STRIPE-seq showed typical TATA-box and Pyrimidine–Purine (PyPu) motifs in the initiator (*Inr*) elements both (Supplementary Fig. S2), which were enriched at +30 bp and +1 bp upstream of the identified TSRs, respectively (Fig. 1D). These observations further underscore the efficacy of STRIPE-seq in identifying TSRs in soybean. In contrast, the TSSs in the soybean Williams 82 reference genome were annotated solely based gene annotation software without support from any experimental data, and thus unsurprisingly, those annotated TSSs did not exhibit either the TATA-box motif or the PyPu motif (Supplementary Fig. S2), echoing our observation that >97% of those annotated TSSs in the reference genome were not supported by the STRIPE-seq data.

### Genes with multiple TSRs exhibit higher levels of expression and experienced stronger purifying selection than those with a single TSR

**Figure 2.**
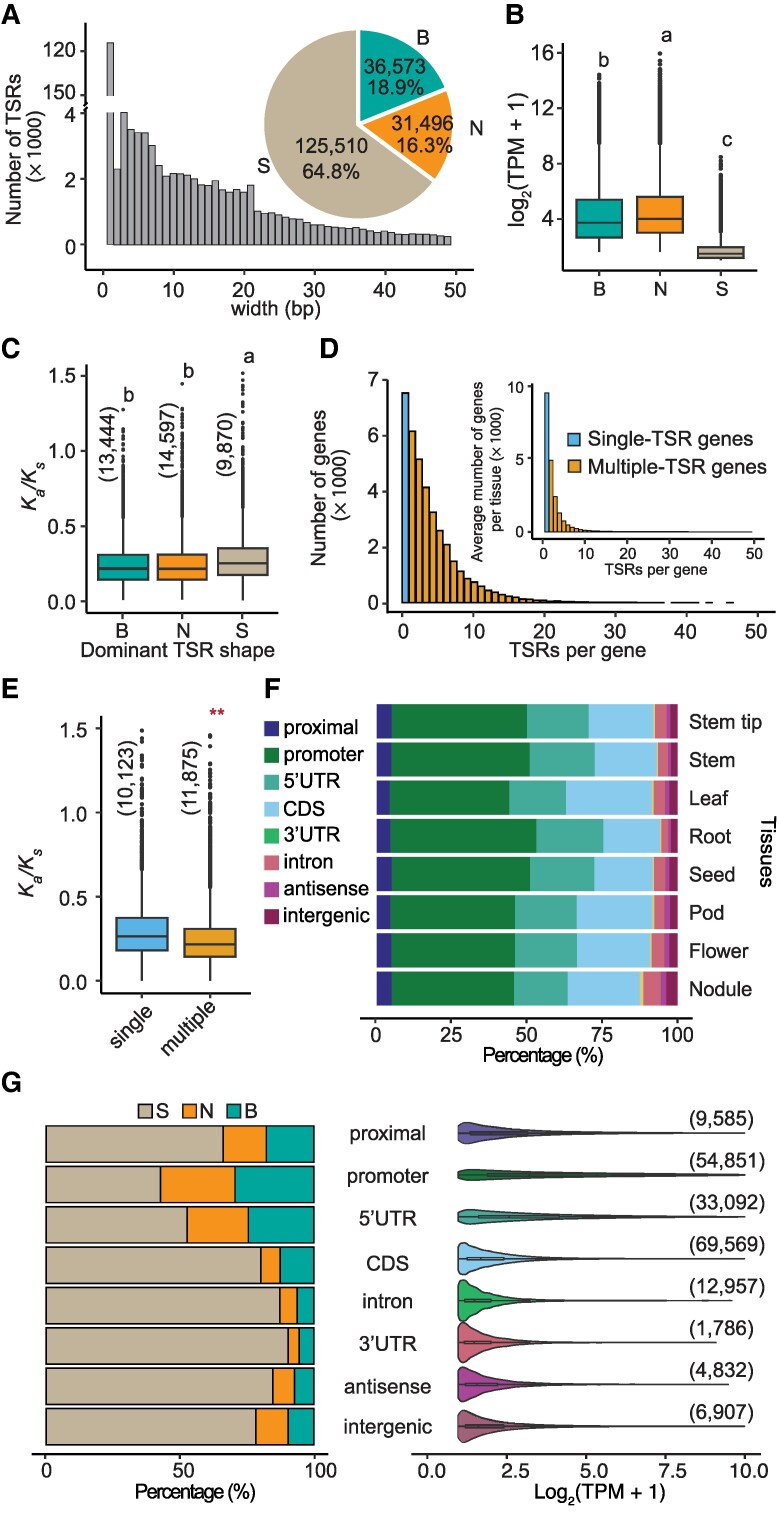
The characteristics of TSRs detected by STRIPE-seq in soybean. **A)** The bar plot displays the length distribution of TSRs. The number and proportion of the 3 TSR shapes are displayed in the pie chart. Only the length of TSRs that are <50 bp were displayed in the bar plot. **B)** The expression levels of genes with different TSRs. The values depicted represent TPM, which is a normalized expression level calculated using the reads from STRIPE-seq for a given gene. The number of the 3 TSR shapes was present in the pie chart in panel **A**. Statistical analysis was performed using one-way ANOVA with multiple comparisons, and significant differences are indicated by letters on the plot. **C)** The *K_a_/K_s_* value of TSRs in the 3 shape categories. The numbers in parentheses represent the sample size. Statistical analysis was performed using one-way ANOVA with multiple comparisons, and significant differences are indicated by letters on the plot. **D)** The number distribution of TSRs per gene. The outer and inner bar plots display the distribution in total and average of each tissue, respectively. **E)** The *K_a_/K_s_* value of TSRs in single and multiple-TSR genes. “single” and “multiple” represent single-TSR and multiple-TSR genes, respectively. The numbers in parentheses represent the sample size. **F)** The relative proportion of TSRs in different annotated features in each tissue. **G)** The left panel shows the proportion of 3 TSR shapes within each gene feature; The right panel presents the abundance of TSRs, normalized by STRIPE-seq reads (TPM), illustrating the distribution of TSR abundance across different gene features. “B”, “N”, and “S” represent “Broad”-, “Narrow”-, and “Single-base”-shaped TSRs, respectively. The numbers in parentheses represent the sample size. The Kolmogorov–Smirnov (KS) test was performed to assess statistical significance (**P-value < 0.01). In each box plot across all panels, the box borders represent the first and third quartiles, the center line indicates the median, and the whiskers extend to 1.5 times the IQR beyond the quartiles. Dots represent outliers.

The identified 193,579 TSRs varied drastically in length, ranging from a single nucleotide to hundreds of base pairs (Fig. 2A; Supplementary Data Set 1). These TSRs were categorized into 3 types based on their shapes, as defined by interquartile range (IQR) (Fig. 2B): single-nucleotide TSRs (S), narrow TSRs (N, IQR ≤ 4), and broad TSRs (B, IQR > 4) (Thodberg et al. 2019), accounting for 64.8%, 16.3%, and 18.9%, respectively (Fig. 2A). Overall, the genes with “S”-shaped TSRs were expressed at lower levels than those with “N”-shaped TSRs and those with “B”-shaped TSRs according to the STRIPE-seq data (Fig. 2B). In addition, the genes with “S”-shaped TSRs exhibited higher nonsynonymous substitution (*K_a_*)/synonymous substitution (*K_s_*) ratios (i.e. *ω* values), which were estimated through comparison with their respective orthologous genes in common bean (*Phaseolus vulgaris*)—a species that diverged from soybean ∼17 MYA (Zhao et al. 2017), than those with “N”-shaped TSRs and those with “B”-shaped TSRs, suggesting that the former have undergone lower intensities of purifying selection than the latter (Fig. 2C; Supplementary Data Set 2).

To understand whether the association between the TSR shapes and the evolutionary rates of genes, as described above, is a shared pattern in plants, we annotated and classified TSRs in the maize (*Zea mays* cv. B73) reference genome (Schnable et al. 2009) using a set of CAGE data previously generated in maize (Mejía-Guerra et al. 2015) into “S”-shaped, “B”-shaped, and “N”-shaped TSRs, following the criteria used in the analysis of the soybean STRIPE-seq data. The evolutionary rates of the maize genes with defined TSRs were estimated previously through comparison with their respective orthologous genes in sorghum (*Sorghum bicolor*) (Zhao et al. 2017)—a species that diverged from maize ∼20 MYA (Ilic et al. 2003). The combination of these 2 sets of data reveals that the maize genes with “S”-shaped TSRs have experienced lower intensities of purifying selection than those with “B”- and “N”-shaped TSRs (Supplementary Fig. S3). Given >100 million years of divergence between the maize as a monocot crop and soybean as a dicot crop (Wolfe et al. 1989), this similarity between the 2 crops suggests an association between the TSR architecture and functional constraints of protein-coding genes in plants.

Of the 37,911 soybean genes with defined TSR by STRIPE-seq, 30,378 (80.1%) were found to contain multiple TSRs, either within the same tissue or across different tissues, and of these multi-TSR genes, 88.3% had fewer than 10 TSRs (Fig. 2D; Supplementary Fig. S4). When TSRs in individual tissues were analyzed separately, on average 57.8% of the 37,911 genes were found to have multiple TSRs. Furthermore, genes with multiple TSRs either within the same tissue or across different tissues exhibited lower *K_a_*/*K_s_* ratios than those with a single TSR (Fig. 2E; Supplementary Fig. S5A). When the 37,911 genes were divided into 4 quantiles based on their expression levels from the lowest (q1) to the highest (q4) in each of the 8 tissues, genes showing higher expression levels (in higher quantiles) exhibited lower *K_a_*/*K_s_* ratios compared with those showing lower expression levels (Supplementary Fig. S5B). As positive correlations between TSR numbers and levels of gene expression were detected in each of the 8 tissues (Supplementary Fig. S5C), it remains unclear whether higher expression levels, or higher TSR numbers, or both drive stronger purifying selection on these genes.

Approximately 67.7% of the 37,911 genes were found to possess more than 1 of the 3 TSR shapes either within the same tissue or across different tissues. When TSR shapes in individual tissues were analyzed separately, on average, 43.0% of these genes were found to have multiple TSR shapes (Supplementary Data Set 2). Thus, a large proportion of genes possessed tissue-specific TSRs and TSR shapes.

### Noncanonical TSRs in intragenic regions are widespread and have distinct features from canonical TSRs

In general, TSRs are expected to be located in regulatory regions such as proximal sequences, core promoters, or 5′ untranslated regions (UTRs), and are referred to as canonical TSRs (Fig. 1A). We found that 97,528 (50.4%) of the TSRs defined in this study fell into the category of canonical TSRs, of which 56.2% were situated in core promoter regions annotated in the reference genome (Fig. 2F). Interestingly, 84,312 (43.5%) of the TSRs were found in annotated introns, CDSs, or 3′ UTRs, dubbed intragenic regions. Of these intragenic TSRs, 82.5% were located in CDSs. These CDS-TSRs (C-TSRs) account for 18.9% of TSRs identified in roots to 28.6% of TSRs in leaves (Fig. 2F). In addition to the TSRs in canonical regulatory and intragenic regions, 6,907 (∼3.6%) of TSRs were positioned in intergenic regions (Fig. 2F), possibly corresponding to noncoding RNA genes, unannotated genes, or even regulatory sequences of nearby or distinct genes. We observed that the canonical regulatory regions had a greater proportion of “B”-shaped TSRs compared with intragenic regions, which harbor the highest proportion of “S”-shaped TSRs (Fig. 2G). Only in the core promoter regions did “S”-shaped TSRs account for <50% of all TSRs (Fig. 2G). In other words, the transcripts initiated at the core promoters were the least tissue-specific.

We then compared sequences surrounding TSRs in different regions. TATA-box elements were detected 30 bp upstream of TSRs in the canonical regulatory and antisense, and intergenic regions (Supplementary Fig. S6). In contrast, the TATA-box elements were barely seen around intron-TSRs and were not detected around C-TSRs and 3′ UTR-TSRs (Supplementary Fig. S6). The distribution of dinucleotide PyPu elements was considerably consistent among different categories of the TSR regions, with CA and TG predominating. However, slight variations were observed; for example, GG was most enriched in C-TSRs but least enriched in core promoter-TSRs (P-TSRs) (Supplementary Fig. S7). We also compared the relative abundance of P-TSRs and C-TSRs for specific genes with both categories of TSRs identified in the same tissue (Supplementary Fig. S8). Most of the genes exhibited more abundant P-TSRs than C-TSRs, whereas 4.5% to 9.8% of genes in the 8 tissues showed more abundant C-TSRs than P-TSRs. Together, these observations suggest that transcription in CDSs may be initiated via mechanisms distinct from those in the core promoters located within canonical regulatory regions.

### ATI was shaped by WGD and the subsequent subgenome fractionation

**Figure 3.**
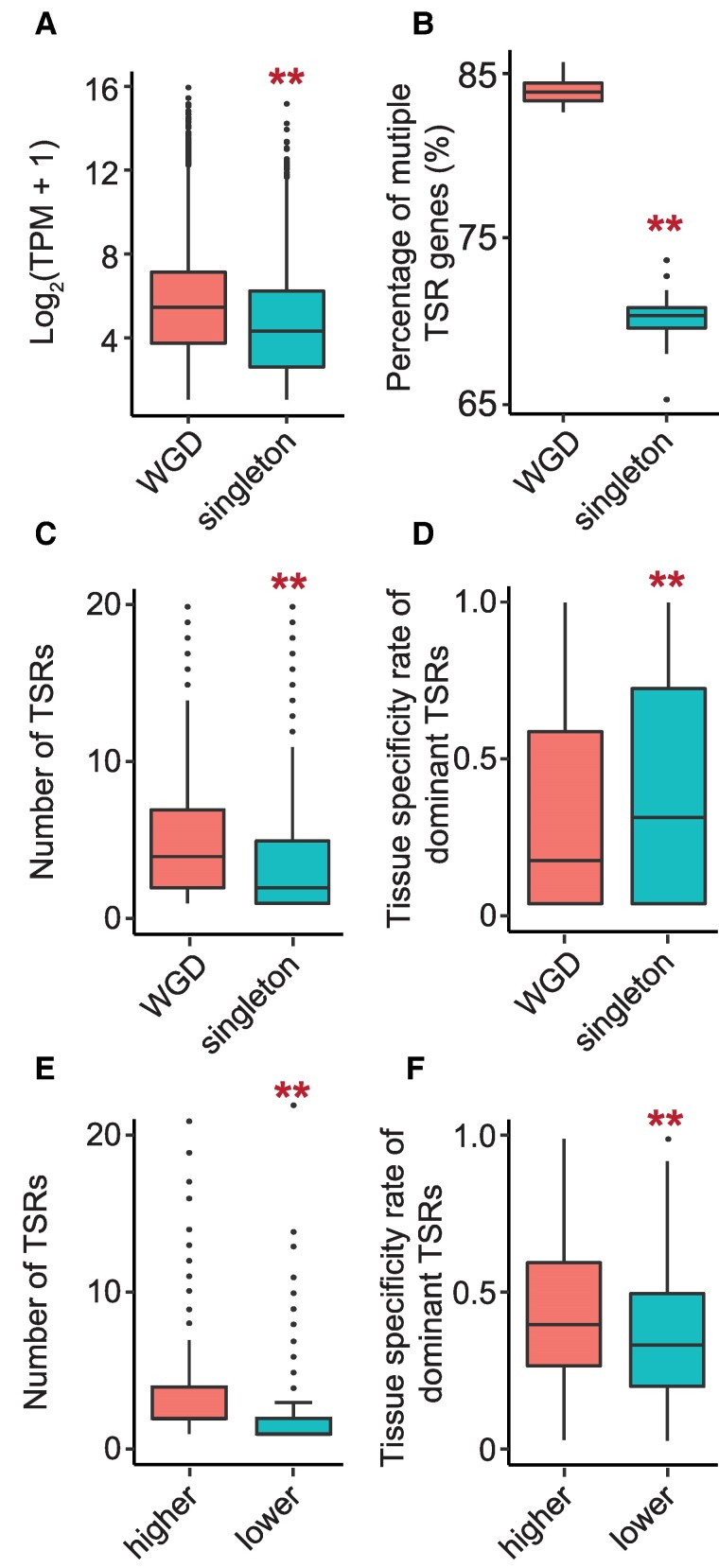
The characteristics of TSR divergence after WGD in soybean. **A)** The transcript abundance of the genes in each gene group. The transcript abundance was normalized by TPM and log2 transformed. **B)** The percentage of multiple TSR genes in each gene group. **C)** The number of TSRs in 1 gene in each gene group. **D)** The tissue-specific rate of dominant TSRs in each gene group. The numbers of WGD and singleton genes shown in the figures are 4,431 and 17,567, respectively. **E)** The number of TSRs in 1 gene in highly expressed WGD copies and lowly expressed WGD copies (*n* = 3,002). **F)** The tissue-specific rate of dominant TSRs in high-expressed WGD copies and lower-expressed WGD copies (*n* = 3,002). Only 2 copies of each of the WGD pairs showing a 2-fold difference in expression levels were included in this comparison. The *y*-axes showed the average values of all genes in the same category labeled on the *x*-axes per gene. The KS test was performed to assess statistical significance in the figure (**P-value < 0.01). In each box plot across all panels, the box borders represent the first and third quartiles, the center line indicates the median, and the whiskers extend to 1.5 times the IQR beyond the quartiles. Dots represent outliers.

Functional divergence of duplicated genes is often reflected by their diverged expression patterns (Zhao et al. 2017). To understand how TSRs of duplicated genes, retained after the recent WGD event, have evolved to contribute to their functional divergence, and how TSRs of duplicated genes and singletons have been shaped by the subgenome fractionation process, we analyzed expression patterns and ATI of WGDs and singletons across 8 tissues and found the following patterns: (i) Consistent with previous reports (Zhao et al. 2017), duplicated genes were generally expressed at higher levels than singletons (Fig. 3A; Supplementary Fig. S9); (ii) duplicated genes possessed more TSRs than singletons (Fig. 3, B and C; Supplementary Fig. S9); (iii) duplicated genes exhibited fewer tissue-specific TSRs than singletons (Fig. 3D; Supplementary Fig. S9). When comparing 2 members of each of the duplicated gene pairs with detected TSRs, we found that the members expressed at higher levels possessed more TSRs and fewer tissue-specific TSRs than their duplicates (Fig. 3, E and F; Supplementary Fig. S9).

Like soybean, maize is a paleopolyploid, proposed to originate from a WGD event ∼11 MYA. However, the consequences of the WGD events forming these polyploids and triggering subsequent subgenome differentiation were quite different. For example, approximately 15% of the WGD pairs are retained in maize after extensive, biased subgenome fractionation, whereas >50% of the WGD gene pairs are retained in soybean without obvious subgenome dominance (Du et al. 2012; Woodhouse et al. 2014; Zhao et al. 2017). To better understand the evolutionary consequences of WGD on ATI in plants, we compared the TSR architecture of WGD and singletons in the maize genome using the CAGE data (Mejía-Guerra et al. 2015), as well as the evolutionary rates of the 2 categories of genes (Zhao et al. 2017). We found that, similar to what was observed in soybean, overall, the duplicated genes possessed more TSRs and were expressed at higher levels than the singletons (Supplementary Fig. S10, A to C). In addition, the members of WGD pairs expressed at higher levels possessed more TSRs than their duplicates (Supplementary Fig. S10D). Our previous analyses in soybean and maize have demonstrated that WGD genes have undergone higher intensities of purifying selection than singletons and that the members of WGD pairs expressed at higher levels have undergone higher intensities of purifying selection than their duplicates in both soybean and maize (Du et al. 2012; Zhao et al. 2017). All these observations together suggest that the distribution patterns of TSRs are important indicators of the functional significance, and evolutionary conservation and divergence of duplicated genes in plant genomes.

### ATI in CDSs tend to be tissue-specific and likely execute tissue-specific functions

**Figure 4.**
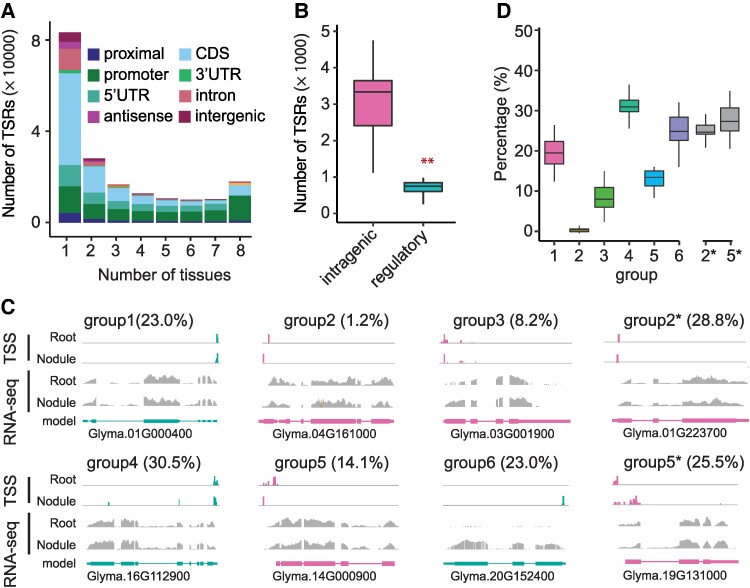
ATI among different tissues in soybean. **A)** The number of TSRs present in a given number of tissues. Different colors represent different annotated features. **B)** The number of tissue-specific TSRs in intragenic and regulatory regions (*n* = 32). The KS test was performed to assess statistical significance (**P-value <0.01). **C)** Examples of TSRs in root and nodule to illustrate the definition of each group. The red and green colors represent the sense and antisense TSRs relative to the reference genome. The gray bars represent the read coverage of RNA-seq in a given gene, whereas the bar at the bottom represents the structure of the gene models. The value inside the parentheses represents the percentage of genes in each group when comparing roots with nodules. **D)** The percentage of genes in each group when comparing between 2 tissues. Each percentage reflects one of the 28 possible pairwise tissue comparisons (*n* = 28). The number on the *x*-axis represents the group number. While percentages for groups 1 to 6 are calculated based on the total number of genes, the percentages for Group 2* and Group 5* are calculated relative to the total numbers of genes in Groups 2 and 5, respectively. In each box plot across all panels, the box borders represent the first and third quartiles, the center line indicates the median, and the whiskers extend to 1.5 times the IQR beyond the quartiles.

To understand the functional significance of ATI, we analyzed the distribution patterns of all TSRs across the 8 tissues. Of the 193,579 TSRs, 62.7% (121,353) were found to be tissue-specific, while 5.5% (10,712) were shared by all 8 tissues (Fig. 4A; Supplementary Table Data Set 1). Of the tissue-specific TSRs, 93.1% were “S”-shaped, while 3.7% were “B”-shaped (Supplementary Fig. S11). Interestingly, tissue-specific TSRs were least abundant in roots but most abundant in root nodules—a root-specific organ in most legumes (Supplementary Fig. S12A), suggesting that these TSRs plays an important role in maintaining tissue identity or executing tissue-specific biological functions such as symbiosis. Strikingly, 45.6% of the tissue-specific TSRs were found in CDSs and likely enable the production of new proteins. By contrast, only 17.7% of the tissue-specific TSRs were in annotated core promoter regions (Fig. 4A). These tissue-specific C-TSRs and tissue-specific TSRs located in introns and 3′ UTRs (together referred to as intragenic TSRs) were ∼4 times as many as the tissue-specific TSRs located within the canonical core promoter regions (P-TSRs) (Fig. 4B; Supplementary Fig. S12B), illustrating the magnitude of ATI for tissue-specific gene regulation.

To dive further into the tissue specificity of ATI, we performed pairwise comparisons of TSRs among the 8 tissues and then categorized genes into 6 groups based on the distribution patterns of TSRs between a tissue pair (Fig. 4C): Group 1—genes with a single TSR shared between 2 tissues compared; Group 2—genes with a single TSR in each tissue but not shared between the 2 tissues. Group 3—genes with multiple TSRs, all shared between 2 tissues compared. Group 4—genes with multiple TSRs, among which the dominant TSR was shared between 2 tissues compared but at least 1 TSR was not shared. Group 5—genes with multiple TSRs, of which the dominant TSRs in each of the 2 tissues were not shared. Group 6—genes with TSRs present in only 1 of the 2 tissues compared, with no TSR detected in the counterpart. The Group 2 genes were least abundant, and the Group 4 genes were most abundant. A total of 15.3% of expressed genes in roots and nodules exhibited ATI between the 2 tissues (Fig. 4D).

To shed light on potential functional consequences of ATI, we extracted subsets of the Group 2 and Group 5 genes (dubbed Group 2* and Group 5*), in which alternative C-TSRs were predicted to result in truncated proteins or frameshifts that potentially affect the genes’ functions. The group 2* and 5* genes account for 24.8% and 27.4% of all genes within Group 2 and Group 5, respectively (Supplementary Data Set 3). As exemplified in Fig. 4C, Glyma.01G223700, a putative USO1-like intracellular protein transport protein gene, was predicted to produce an N-terminus-truncated protein in the roots that is 67 amino acids shorter than the protein produced in the nodules. Glyma.19G131000, a putative ankyrin repeat family gene, was predicted to generate an N-terminus-truncated protein in the nodules that is 92 amino acids shorter than the protein generated in the roots (Fig. 4C). These tissue-specific protein truncations or frameshifts mediated by ATI are potentially associated with their tissue-specific functions.

### Antisense and sense TSRs exhibited similar distribution patterns along genic sequences, and so did bidirectional TSRs

**Figure 5.**
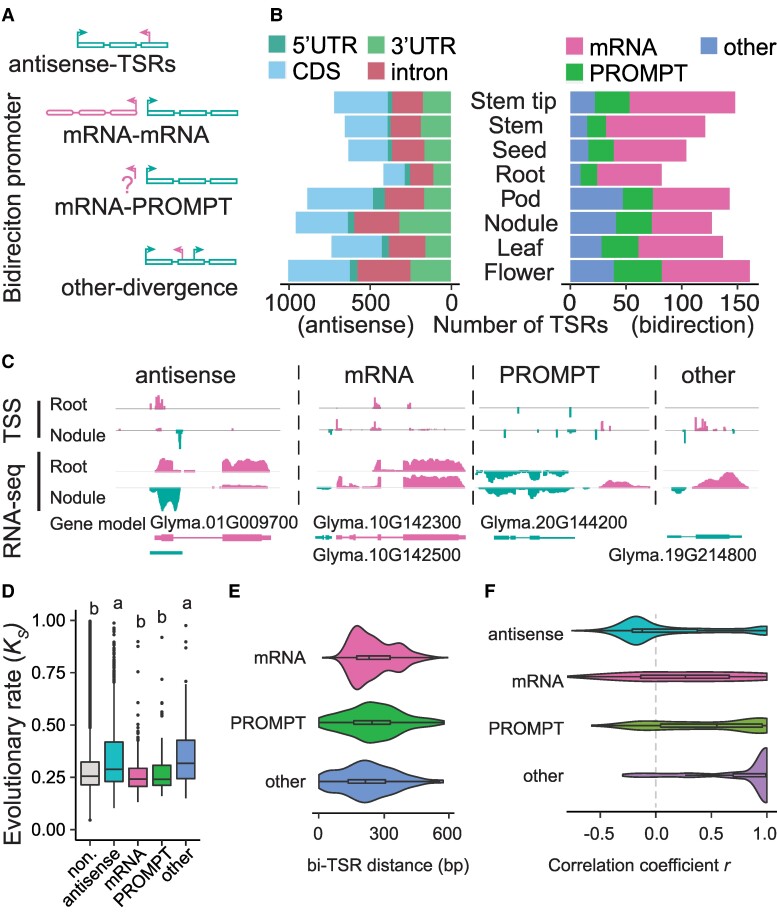
The characteristics of antisense- and bidirectional TSRs in soybean. **A)** Schematic representation of the categories of antisense- and bidirectional TSRs. The rectangle represents the exons of a given gene. **B)** The number of antisense-TSRs (left panel) in annotated gene features and the catteries of bidirectional TSRs (right panel) in each tissue. **C)** Examples of antisense- and bidirectional TSRs in the root and nodule tissues. **D)** The evolutionary rate (*K_s_*) in genes with different TSR categories. “non.” represents genes without antisense- and bidirectional TSRs. Statistical analysis was performed using one-way ANOVA with multiple comparisons, and significant differences are indicated by letters on the plot. **E)** The distance distribution of 3 types of bidirectional TSRs. The *x*-axis represents the longest distance between 2 directional TSRs in 1 bidirectional TSR. **F)** The correlation of antisense-TSR and the sense TSR abundance among tissues in each category. “antisense”, “mRNA”, “PROMPT”, and “other” represent bidirectional TSRs belonging to the antisense-TSR, mRNA-mRNA, mRNA-PROMPT, and other- divergence categories, respectively. In each box plot across all panels, the box borders represent the first and third quartiles, the center line indicates the median, and the whiskers extend to 1.5 times the IQR beyond the quartiles.

The strand-specific nature of STRIPE-seq allowed us to determine antisense and bidirectional TSRs and their relative abundance. An antisense TSR was defined when a gene with sense TSR was detected to have a TSR producing overlapping transcripts in the opposite direction (Fig. 5A). In the 8 soybean tissues, we identified 4,574 antisense TSRs, accounting for 2.36% of all TSRs identified in this study, which were assigned to 3,170 genes (Supplementary Data Set 4). These antisense TSRs were predominantly tissue-specific, comprising 85% of all the antisense TSRs. In addition, the antisense TSRs were most frequently found in the CDS regions, harboring 39.5%, on average across tissues. In contrast, only 5.2% of the antisense TSRs were found in the 5′-UTR regions (Fig. 5B). Antisense TSRs were also observed in intronic sequences. For example, in nodules, we detected an antisense TSR and corresponding transcripts in *Glyma.01G009700*, a gene putatively encoding a transmembrane protein. This antisense TSR originated within the intronic region of the annotated sense transcripts (Fig. 5C).

Bidirectional TSRs were defined when they were found within 600 bp and initiated transcription in opposite directions (Fig. 5A). They were further categorized into 3 subgroups based on their relationships with the annotated TSRs in the reference genome (Fig. 5A): (ⅰ) mRNA-mRNA: 2 bidirectional TSRs correspond to the promoters of 2 different genes; (ⅱ) mRNA-PROMPT: 1 of the 2 bidirectional TSRs corresponds to the promoter of 1 gene, but the other is located in the upstream region of the promoter of the same gene and does not correspond to a second gene. This upstream bidirectional TSR is referred to as a promoter upstream transcript (PROMPT); and (ⅲ) other-divergence: 2 bidirectional TSRs are both located within an intragenic region of a single gene. We identified 506 bidirectional TSRs in the 8 soybean tissues, including 156 mRNA-mRNA pairs, 162 mRNA-PROMPT pairs, and 184 other-divergence pairs, corresponding to 664 genes. Each direction accounting for 0.14% of all TSRs identified in this study. (Figure 5B and Supplementary Data Set 4). These bidirectional TSRs, particularly mRNA-PROMPT and other divergent forms, also showed a high degree of tissue specificity (Fig. 5B; Supplementary Data Set 5). We also observed an “mRNA-mRNA” type of TSR interaction between Glyma.10G142300, which generates a potential noncoding RNA, and Glyma.10G142500, which encodes a neurofilament-like protein (Fig. 5C). Additionally, an “mRNA-PROMPT” type of TSR was identified in Glyma.20G144200, a gene encoding a putative protein belonging to the major facilitator superfamily (Fig. 5C). An example of the “other-divergence” type of TSR was found in Glyma.19G214800, whose putative ortholog in cotton is involved in fiber expression. The antisense transcript of Glyma.19G214800 was initiated in its intronic region (Fig. 5C). These specific TSRs in nodules were supported by RNA-seq data, although the data could not precisely define the boundaries of the TSRs (Fig. 5C).

Our analysis also reveals that the genes producing antisense and “other-divergence” TSRs have evolved at an overall faster pace, as reflected by *K_s_*, than the genes without antisense or “other-divergence” TSRs (Fig. 5D; Supplementary Fig. S13A). Interestingly, the former has undergone stronger intensities of purifying selection than the latter (Supplementary Fig. S13B), suggesting the functional constraints of these alternative forms of transcripts. On average, the distance between bidirectional TSRs is ∼250 bp, close to the length of DNA between 2 nucleosomes (Fig. 5E). Notably, the expression of antisense TSRs was generally negatively correlated with the expression of corresponding sense TSRs, likely minimizing the production of sRNAs from paired transcripts of the same genes, as seen in the expression pattern of Glyma.01G009700 and its antisense transcripts in root and nodule tissues (Fig. 5, C and F). All categories of bidirectional TSR pairs showed positive correlations in transcript abundance, suggesting a lack of functional interference between the transcripts produced by bidirectional TSRs (Fig. 5F).

### TSRs in core promoters and coding regions exhibited distinct epigenomic features

Genetic and epigenetic features of canonical core promoter regions have been well documented. To understand how ATI in intragenic regions, particularly CDSs, is triggered, we examined key histone modifications (H3K4me3, H3K56ac, H3K36me3, H3K4me1, and H3K27me3) along with histone protein markers (H3), around TSRs with genome-wide chromatin profiles generated from soybean leaves (Lu et al. 2019). H3K4me3, H3K56ac, and H3K36me3 are active transcription-associated histone marks, whereas H3K27me3 is repressive transcription-associated histone mark (Guenther et al. 2007; Zhao et al. 2019). H3K4me1 exhibits diverse effects on transcriptional activity across different organisms. In animals, H3K4me1 is associated with marking poised enhancers, while in plants, it has been implicated in various processes including DNA repair, RNA polymerase II elongation, and facilitation of RNA-directed DNA methylation (Policastro et al. 2020; Niu et al. 2021; Quiroz et al. 2024). In addition, there is evidence suggesting the repressive role of H3K4me1 in intragenic transcription initiation (Nielsen et al. 2019). Furthermore, a study demonstrates that H3K4me1, while correlated with gene expression levels, is more strongly linked to a poised chromatin state, which as defined by the simultaneous presence of H3K4me3 and H3K27me3, rather than to transcriptional activity, suggesting a possible role for H3K4me1 in epigenetic memory (Bae and Lesch 2020).

Overall, the TSRs in core promoter regions (P-TSRs) of all expressed genes in the 8 tissues were characterized by typical promoter-like chromatin architecture, as represented by overlapping peaks of H3K4me3, H3K56ac, and H3K36me3 (Supplementary Fig. S14). Such peaks were also detected around TSRs identified in the intergenic intervals and around TSRs to produce antisense transcripts. By contrast, the intragenic regions overall exhibited a relatively even distribution of H3K4me3, H3K56ac, and H3K36me3, which were less abundant in these regions than at the peaks near the canonical core P-TSRs (Supplementary Fig. S14).

**Figure 6.**
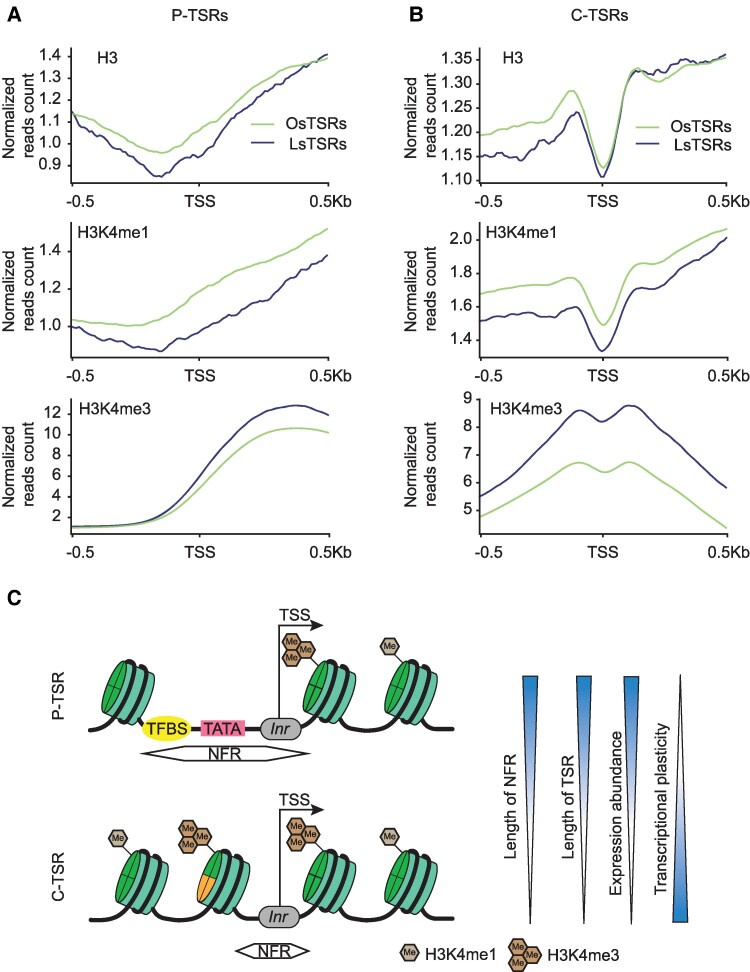
The distributions of histone modifications around the leaf-specific transcription start regions (LsTSRs) and the TSRs specific to each of the other 7 tissues (OsTSRs) located in given features in leaves. **A)** The distribution of markers around promoter (P)-TSRs. The numbers of OsTSRs and LsTSRs in promoter are 19,418 and 2,075, respectively. **B)** The distribution of markers around CDS-TSRs, C-TSRs. The numbers of OsTSRs and LsTSRs in CDS are 46,733 and 8,553, respectively. TSS was defined as the peak site of the TSR. **C)** Proposed model of nucleosome accessibility and histone modifications around P-TSRs and C-TSRs. The arrow represents the initial location and direction of transcription. C-TSRs and P-TSRs refer to TSRs located in CDS and promoter regions, respectively. *Inr* represents the initiator element.

Since the C-TSRs were the predominant intragenic TSRs identified in the 8 tissues and were highly tissue-specific, and the histone modification profiles were only generated from soybean leaf tissue (Lu et al. 2019), we subsequently focused on comparison between the leaf-specific C-TSRs (LsTSRs) and the C-TSRs specific to each of the other 7 tissues (OsTSRs) in the context of epigenomic features in leaves (Fig. 6, A to C; Supplementary Fig. S15). A relatively low abundance of the H3 curve (representing a nucleosome-free region or NFR) was observed at LsTSRs, suggesting that, like that in the canonical promoter regions, the chromatin accessibility is critical for the occurrence of transcription initiation in CDSs. Nevertheless, NFRs around C-TSRs were substantially shorter than those around P-TSRs (Fig. 6, A and B), potentially explaining why fewer TATA-box motifs were found in the former than in the latter Supplementary Fig. S6). Interestingly, a relatively low abundance H3K4me1 peak was observed around the C-TSRs, which was flanked by peaks of H3K4me3, H3K56ac, and H3K36me3 in leaves (Fig. 6B; Supplementary Fig. S14). When the epigenetic marks around P-TSRs and C-TSRs of genes defined in the 4 quantiles based on their expression levels in the leaf tissue were analyzed separately, we observed consistent distribution patterns among the 4 quantiles were observed (Supplementary Fig. S16), suggesting that the physical locations of P-TSRs vs. C-TSRs are more critical in determining enrichment than their expression levels.

More intriguingly, the C-TSRs in each of the 7 tissues were found to correspond to or be adjacent to the H3 curve and H3K4me3, H3K56ac, and H3K36me3 peaks detected in leaves (Fig. 6B; Supplementary Fig. S15). In addition, these active transcription-related markers detected in leaves were found to be more enriched around C-TSRs specifically present in leaves than those around C-TSRs present in the other 7 tissues. By contrast, H3K4me1 in leaves was found to be less enriched around C-TSRs specifically present in leaves than those around C-TSRs present in the other 7 tissues (Fig. 6, A and B; Supplementary Fig. S15). These observations suggest that H3K4me1 may indeed play a key role in epigenetic memory associated with transcription initiation and interplay with the H3K4me3, H3K56ac, and H3K36me3, etc. to determine whether a specific ATI in a CDS occurs in a particular tissue.

## Discussion

Our study provides a comprehensive soybean TSR atlas that complements current genome annotation and various transcriptomic data for the research community. STRIPE-seq can identify TSSs and simultaneously evaluate relative abundances of transcripts from individual TSSs using as little as 50 ng of total RNA, this enabled our study to effectively identify TSR distribution and ATI, as well as assess their tissue specificity and relative abundance with high precision at a low cost. Despite the advantages of the STRIPE-seq approach for ATI discovery, we would like to note that it lacks capability to reveal the full spectrum of isoforms resulting from specific ATI. As such, it was difficult to define specific protein changes mediated by ATIs. This disadvantage may be overcome by generating and integrating long-read or full-length transcripts processed for STRIPE-seq, or by optimizing the current STRIPE-seq protocol to enable enrichment and sequencing of the full-length transcripts from total RNA samples. We would like to note that the TSR numbers and shapes across genic sequences including CDSs were defined under relatively stricter criteria to ensure their authenticity; nevertheless, such criteria would unavoidably exclude TSRs that were expressed at low levels. A combination of STRIPE-seq with additional approaches to profiling TSSs may help to validate those lowly expressed TSRs.

Some of the genomic and epigenomic features of TSRs are shared among species. In both soybean and maize, the duplication status of genes was associated with TSR architecture, expression levels, evolutionary rates, and functional constraints of associated genes. Given that nearly all flowering plants have experienced one or multiple rounds of WGD (Jiao et al. 2011), these associations may reflect common mechanisms regulating gene expression and evolutionary forces shaping or reshaping plant genomes over evolutionary time. Regardless of the duplication status of the soybean genes, the “S”-shaped TSRs were linked to lower expression levels compared with “N”-shaped and “B”-shaped TSRs, while no difference in expression levels was seen between “N”-shaped and “B”-shaped TSRs. At first glance, this pattern seemed to be inconsistent with an earlier observation in maize, where genes with sharp TSRs were expressed at lower levels than those with broad TSRs (Mejía-Guerra et al. 2015). However, the category of sharp TSRs in maize included both “S”-shaped TSRs and sort of “N”-shaped TSRs according to the categorization criteria in soybean; thus, the detected lower expression of genes with sharp TSRs in maize can be explained by the lower expression of genes with “S”-shaped TSRs compared with those with “N”-shaped and “B”-shaped TSRs. Given that the maize genome possesses much more singletons compared with duplicated gene pairs than the soybean genome, a higher proportion of genes with “S”-shaped TSRs in the former than in the latter would be expected. Few studies have conducted comprehensive identification and characterization of intragenic TSRs; nevertheless, the genomic and epigenomic features of TSRs situated within the canonical regulatory regions of genes, such as the presence of TATA-box motifs, and relatively broader TSRs, longer NFRs, and lower degrees of tissue specificity compared with those within the intragenic regions are generally conserved among plant species as well as mammals (Morton et al. 2014; Mejía-Guerra et al. 2015; Wang et al. 2019; Le et al. 2020; Policastro et al. 2020; Thieffry et al. 2020). Together, these similar observations from diverse taxa underscore shared strategies, including ATI, for transcriptional regulation in eukaryotes.

Compared with other plant species, 1 striking observation in soybean is the prevalence of genes with multiple TSRs, which account for ∼80% of all genes with detected TSRs when the STRIPE-seq data from all 8 tissues were combined, or ∼58%, on average, when the STRIPE-seq data from individual tissues were analyzed separately. By contrast, only ∼38% of all genes with detected TSSs from 16 tissues of a diploid cotton species *Gossypium arboreum* have multiple TSRs (Wang et al. 2019). In Arabidopsis (*Arabidopsis thaliana*) leaves, this figure is even lower at ∼9% (Thieffry et al. 2020). The retention of more than 50% duplicated pairs of genes in the soybean genome, which were detected to have more TSRs than singletons, may partially explain the prevalence of genes with multiple TSRs. Additionally, several factors could have contributed to such a degree of interspecific difference. These include the sensitivity and effectiveness of the methods used for capturing transcripts with TSSs, the sequencing depth, and the criteria applied for calling of TSRs.

The proportion of divergent TSRs in soybean is considerably lower, a pattern that does not align with findings in vertebrates, where prevalent bidirectional TSRs were observed and were found to be closely associated with eRNAs (Andersson et al. 2015; Thieffry et al. 2020). However, this phenomenon is not exclusive to soybean. It has also been observed in both Arabidopsis and maize, suggesting that divergence in TSRs, while less frequent, is likely a common feature in the plant kingdom (Mejía-Guerra et al. 2015; Thieffry et al. 2020). Thieffry et al. (2020) postulated that the suppression of divergent TSRs in plant genomes may be linked to the unique small RNA landscape. For example, the accumulation of small RNAs around PROMPTs in exosome mutants, especially the 24 nt sRNAs, suggests the involvement of the RNA-directed DNA methylation pathway—a plant-specific DNA methylation pathway absent in mammals (Thieffry et al. 2020).

Another contrasting observation in soybean compared with other plant species is the abundance of ATI in intragenic regions, particularly those in CDSs (C-ATI), which potentially result in changes of amino acid sequences. Based on our data from soybean and previous observations from maize (Mejía-Guerra et al. 2015), the frequency of C-ATI with changes in amino acids in soybean is more than 20 times higher than that in maize. The reasons for such a significant difference between the 2 plant species are yet to be investigated, but the different numbers of tissues (8 in soybean vs. 2 in maize) and different methods (STRIPE vs. CAGE) used for identification of ATI may be partially responsible. C-ATI can have significant biological outcomes, such as changes in protein subcellular localization and function (Mejía-Guerra et al. 2015; Tokizawa et al. 2017). Some of the changes have been found to have phenotypic effects such as cancer development in humans (Davuluri et al. 2008) and nitrate uptake activity in plants (Wang et al. 2019). ATI in the UTRs can also be of biological significance, such as the gain or loss of regulatory motifs critical for binding transcription factors and other regulatory proteins, and alternation of gene expression patterns, mRNA stability, localization, or translational efficiency (Chen and Tarn 2019; Alfonso-Gonzalez and Hilgers 2024). The current knowledge about the functional consequences of ATI remains limited, partially due to the lack of well-characterized ATI atlas in most organisms. Hence, this study provides a much-needed ATI dataset, laying a foundation for future studies aimed at unraveling the roles of ATI in shaping the plasticity of plant phenotypes.

Our analysis revealed distinct genomic and epigenomic features such as nucleosome depletion lengths, histone occupancy patterns, and histone modification signals, distinguishing TSRs in canonical core promoters from those in intragenic regions. Currently, the precise mechanisms driving the widespread, tissue-specific ATI within CDSs remain largely unknown. Nevertheless, the presence of more confined NFRs with fewer TATA-box motifs in TSRs within CDSs compared with those in core promoters suggests the involvement of fewer general transcription factors to initiate transcription. This may partially explain the higher degree of tissue specificity of TSRs within CDSs compared with those in core promoters. Additionally, as the H3 curve, and the active and repressive transcription-associated histone modification signals around TSRs in CDSs in leaves are shared by other tissues where these TSRs are even not detected, it seems clear that the relative abundances of different histone modifications and their dynamic interplays as illustrated in Fig. 6C, as well as genetic regulators binding to the NFRs are largely responsible for tissue-specific ATI in CDSs. Further investigation is needed to understand the genetic and epigenetic factors underlying the complex regulatory landscapes in plants.

## Materials and methods

### Plant growth conditions and material collection

Soybean (*Glycine max*) cultivar Williams 82 was grown in the greenhouse under a 16 h light/8 h dark period. Eight tissues, including root, nodule, stem, stem tip, leaf, flower, pod, and seed, were collected at the development stages of trefoil, flowering, and seed development. *Bradyrhizobium diazoefficiens* USDA 110 was used for soybean root inoculation. The nodules on inoculated roots 20 d postinoculation and uninoculated roots at the same stage were prepared and harvested as previously described (Ren et al. 2019). Each sample was collected from at least 8 individual plants and quickly frozen in liquid nitrogen for RNA isolation.

### RNA isolation, DNA, and rRNA depletion

The total RNA was isolated using the TRIzol reagent (Invitrogen/Life Technologies, CA), according to the manufacturer's instructions. To remove the residual DNA, the extracted RNA was treated with TURBO DNA-free Kit (Invitrogen). The nodule-derived RNA was treated with NEBNext rRNA Depletion Kit (Bacteria) (NEW ENGLAND BioLabs) and RiboMinus Plant Kit for RNA-seq (Invitrogen) sequentially, while the RNA from 7 other tissues was treated with RiboMinus Plant Kit for RNA-seq (Invitrogen) to deplete the rRNAs.

### STRIPE-seq library construction and sequencing

STRIPE libraries were constructed as previously described, and a step-by-step protocol can be found at protocols.io (https://www.protocols.io/view/stripe-seq-library-construction-bdtri6m6) (Policastro et al. 2020). Briefly, to reduce the proportion of uncapped RNA, the DNA, and rRNA-depleted RNA samples were treated with terminator 5′-phosphate-dependent exonuclease (Lucigen). After a 1 h incubation, TSRT was performed using a unique barcoded RTO per sample, followed by the addition of a UMI-containing, 5′-biotin-modified TSO with 3 3′ riboguanosines. Library PCR was then performed using the TSRT product as input, which ensures that TruSeq adapters are present on both sides of the insert. According to the initial amount of the DNA and rRNA-depleted RNA samples, 16 to 20 cycles of PCR with the forward library oligo (FLO) and reverse library oligo (RLO) were performed in this step. Solid phase reversible immobilization bead size selection is used to remove fragments that are outside the ideal size. Final libraries distributed between 250 and 750 bp with a total amount of 25 to 100 ng were sequenced using the Illumina Novaseq 6000 platform to generate 150 bp paired-end reads at UC Davis Sequencing Center (Novogene Corporation Inc.). See Supplementary Table S2 for RTO, TSO, FLO, and RLO sequences.

### Processing of STRIPE-seq data

First, the raw read quality was estimated with the “fastQC” program (version 0.11.9, https://www.bioinformatics.babraham.ac.uk/projects/fastqc/). Only read one of each read pair was used for the following analysis because it represents the sense direction of RNA. The adapter sequences and poly(G) sequences were trimmed with the “cutadapt” program (version 2.5) (Martin 2011). Trimmed reads were removed if they were <50 nucleotides or lacked a TATAGGG sequence pattern. Then, the redundant reads amplified by PCR were removed by taking advantage of UMI sequences using the “fasx_collapser” function in the “fastx-toolkit” program (version 0.0.14, https://github.com/agordon/fastx_toolkit). The TATAGGG structure of selected reads was trimmed using the “cutadapt” program. To remove potential rRNA contamination in the data, we collected all the rRNA gene sequences from the soybean reference genome (version2.1, phytozome) and the NCBI database (nt. ftp.ncbi.nlm.nih.gov/blast/db/) (Schmutz et al. 2010). The cleaned reads were mapped to the rRNA sequences using “hisat2” with default parameters. We recalled the reads that did not map to rRNA genes. We further mapped all the recalled reads to the soybean reference genome using “hisat2” and kept the records with mapping quality >30 using the “SAMtools” program (version 1.8) (Li et al. 2009). For nodules, the symbiotic organ, we additionally removed the sequence from the *B. diazoefficiens* USDA 110 genome using the above programs (Kaneko et al. 2002).

### Identification, quantification, and annotation of TSRs

First, we converted the bam files into strand-specific BigWig files, which were used as input files for identification of TSSs using the “CAGEfightR” package (version 1.12.0) in Bioconductor (https://bioconductor.org/) (Thodberg et al. 2019). Initially, we normalized the TSS abundance using the TPM method and retained TSSs with TPM values >1 in at least 1 tissue. This threshold was chosen based on our analysis, which indicated that a TPM value >1 was suitable for retaining TSSs with sufficient read support in individual tissues. To assess the potential impact of varying TSS abundance thresholds on TSR width, we tested different TPM cutoffs (0, 1, 5, and 10) and evaluated their effects on TSR width (1, 10, 100, and 1,000) (Supplementary Table S3). Our analysis revealed that while the distribution of TSR widths did not significantly change between a cutoff of 0 and 1, using a TPM threshold of 1 (equaling to ∼7.3 nonredundant reads with unique UMI per TSS) effectively reduced background noise, such as large TSRs (>1,000 bp), without significantly affecting the distribution of TSR widths. Furthermore, we observed that employing stricter criteria resulted in a reduction in the total number of TSRs, particularly when using higher TSS thresholds. We believe that a TPM threshold of >1 balances between maintaining a clean background and preserving an adequate number of TSRs for analysis. Adjacent TSSs were clustered and merged to TSRs if the distance dropped to 20 bp or less. The abundance of TSRs was quantified by the sum of TPM values of all support TSSs in 1 tissue. The peak location was defined as the position of TSS with maximum abundance in TSRs. To annotate the TSRs in the soybean genome, the Williams 82 reference annotation file (gff3, Phytozome) was used to construct a database for the following analysis by using the “makeTxDbFromGFF” function in the “GenomicFeatures” (version 1.44.2) package in Bioconductor (Goodstein et al. 2012; Lawrence et al. 2013). The promoter region was defined as the region from 100 bp upstream to 100 bp downstream of the annotated TSS in the annotation file. The proximal regions were defined as the regions 400 bp upstream of the promoter regions. The antisense TSRs were annotated if a detected TSR was in the gene body region with reversed direction relative to the annotated transcriptional direction. The overlapping regions were annotated based on the priority annotation level as illustrated in Fig. 1A. The location of TSRs was defined as intergenic if no annotated features overlapped with them. TSRs were defined as bidirectional if the 2 reverse directions of the TSRs were located within the 600 bp region. All TSSs can be accessed through our genome browser portal. They can be visited and explored at http://xtlab.hzau.edu.cn/jbrowser/ (Supplementary Fig. S17). The portal is built using the Jbrowse2 framework (Diesh et al. 2023).

### Estimation of evolutionary distance and selection constraint

The syntenic homologous gene pairs between soybean (*Glycine max*) and common bean (*P. vulgaris*) were defined in previously published data sets (Zhao et al. 2017). To calculate accurate *K_a_*, *K_s_*, and *K_a_/K_s_*, we first performed CDS alignment with the “MUSCLE” program (version 3.8.31) using the default parameters (Edgar 2004). The “yn00” and “baseml” modules in the “PAML” program (version 4.8) were processed to calculate all 3 evolutionary parameters (Yang 2007).

### Sequencing and processing of RNA-seq

Total RNAs of the nodule and root samples were processed using the Epicentre Ribo-zero rRNA Removal Kit (Epicentre, USA) to deplete ribosomal RNAs, and the processed RNA samples were used to construct RNA-seq libraries using the NEBNext Ultra Directional RNA Library Prep Kit for Illumina (NEB, USA). Then, the RNA libraries were sequenced using the Illumina HiSeq 4,000 platform to generate 150 bp paired-end reads. The raw reads were processed with the “fastx-toolkit” program to remove low-quality reads and adaptor sequences. The processed reads from each library were subsequently mapped to the soybean reference genomes using “Hisat2” (version 2.1.0) with the default parameters (Kim et al. 2019). The reads with map quality >30 were extracted for the following analysis using “SAMtools” (version 1.8). The BAM files were visualized using the “IGV” software (version 2.8.13) (Thorvaldsdottir et al. 2013).

### Processing of ChIP-seq

The ChIP-seq of histone modifications of leaf tissue in soybean was collected from a previous study (Supplementary Table S4), which included H3, H3K4me1, H3K4me3, H3K27me3, H2A.Z, H3K56ac, and H3K36me3 (Lu et al. 2019). The reads were preprocessed with the “fastx-toolkit” program. The clean reads were mapped to the soybean reference genome using the “BWA” program with default parameters. We only kept the reads with map quality above 30 using the “SAMtools” program. Then, the BAM files were converted to BigWig files using “BamCoverage” with the parameters (—ignoreDuplicates —normalizeUsing RPGC —effectiveGenomeSize 990741540). The distribution of histone modification signals surrounding the given TSSs was visualized using the “computerMatrix” and “plotProfiles” programs.

### Accession numbers

Sequence data from this article can be found in the National Center for Biotechnology Information Sequence Read Archive (http://www.ncbi.nlm.nih.gov/sra/) under the accession numbers PRJNA757465 and PRJNA757638.

## Supplementary Material

koae288_Supplementary_Data

## Data Availability

Details of tissues and reprocessed public datasets, including methylC-seq and ChIP-seq for histone modifications, are listed in Supplementary Table S4. All scripts for read processing, mapping, and R analyses are available in a GitHub repository (https://github.com/Wanjie-Feng/STRIPE_seq.git).

## Supplementary data

The following materials are available in the online version of this article.

**Supplementary Figure S1.** Evaluation of the quality of sequencing and TSR detection using STRIPE-seq (supports Fig. 1).

**Supplementary Figure S2.** Sequence patterns of TSSs in different annotation datasets (supports Fig. 1).

**Supplementary Figure S3.** The nonsynonymous substitution (*K_a_*)/synonymous substitution (*K_s_*) ratios in genes with different dominant TSR shapes in maize (supports Fig. 2).

**Supplementary Figure S4.** Statistics of the number of genes with a given number of TSRs in each tissue (supports Fig. 2).

**Supplementary Figure S5.** Interplays between gene expression levels, TSR numbers, and intensities of purifying selection (supports Fig. 2).

**Supplementary Figure S6.** Sequence patterns around TSRs in different feature regions (supports Fig. 2).

**Supplementary Figure S7.** The dinucleotide pattern of TSRs in each feature region (supports Fig. 2).

**Supplementary Figure S8.** Comparison of expression levels of P-TSRs and CDS-TSRs for specific genes with both categories of TSRs identified in each of the 8 tissues (supports Fig. 2).

**Supplementary Figure S9.** The number of TSRs in WGD and singleton gene groups in each tissue (supports Fig. 3).

**Supplementary Figure S10.** The characteristics of TSR divergence after WGD in maize (supports Fig. 3).

**Supplementary Figure S11.** The relationship between TSR shape and the number of TSRs present in a given number of tissues (supports Fig. 4).

**Supplementary Figure S12.** The number of tissue-specific TSRs in each tissue and each genic region (supports Fig. 4).

**Supplementary Figure S13.** The evolutionary rate (*K_a_*) and *K_a_/K_s_* in genes with different TSR categories (supports Fig. 5).

**Supplementary Figure S14.** The distributions of histone modifications and variants around TSRs located in a given annotated feature in leaves (supports Fig. 6).

**Supplementary Figure S15.** The distributions of histone modifications around the leaf-specific TSRs (LsTSRs) and the TSRs specific to each of the other 7 tissues (OsTSRs) located in given features in leaves (supports Fig. 6).

**Supplementary Figure S16**. Distributions of histone modifications around TSRs expressed at different levels in the leaf tissue (supports Fig. 6).

**Supplementary Figure S17.** Screenshot showcasing the TSSs on the genome browser webpage (supports Fig. 1).

**Supplementary Table S1.** The statistical summary information of the read number in each process step.

**Supplementary Table S2.** Oligos used for constructing the STRIPE-seq libraries.

**Supplementary Table S3.** Impact of TSS abundance thresholds on TSR definition.

**Supplementary Table S4.** Sources of datasets used in this study.

**Supplementary Data Set 1.** Summary information of detected TSRs.

**Supplementary Data Set 2.** Summary information of genes with detected TSRs.

**Supplementary Data Set 3.** Genes with differential TSRs in CDS and 5′ UTR.

**Supplementary Data Set 4.** Antisense TSRs detected in soybean.

**Supplementary Data Set 5.** Bidirectional TSRs detected in soybean.

## Dive Curated Terms

The following phenotypic, genotypic, and functional terms are of significance to the work described in this paper:

group CHEBI: CHEBI:24433
