# Supplementary material for: Noncanonical transcription initiation is primarily tissue specific and epigenetically tuned in paleopolyploid plants
Source: Plant Cell. 2024 Nov 14;37(1):koae288. doi: 10.1093/plcell/koae288 (PMC11663555; doi:10.1093/plcell/koae288)
Supplement: koae288_Supplementary_Data [file koae288_supplementary_data.zip › TPC2024RA11198DR2_Supplementary_Figures_1_17_Supplementary_Tables_1_4.pdf]

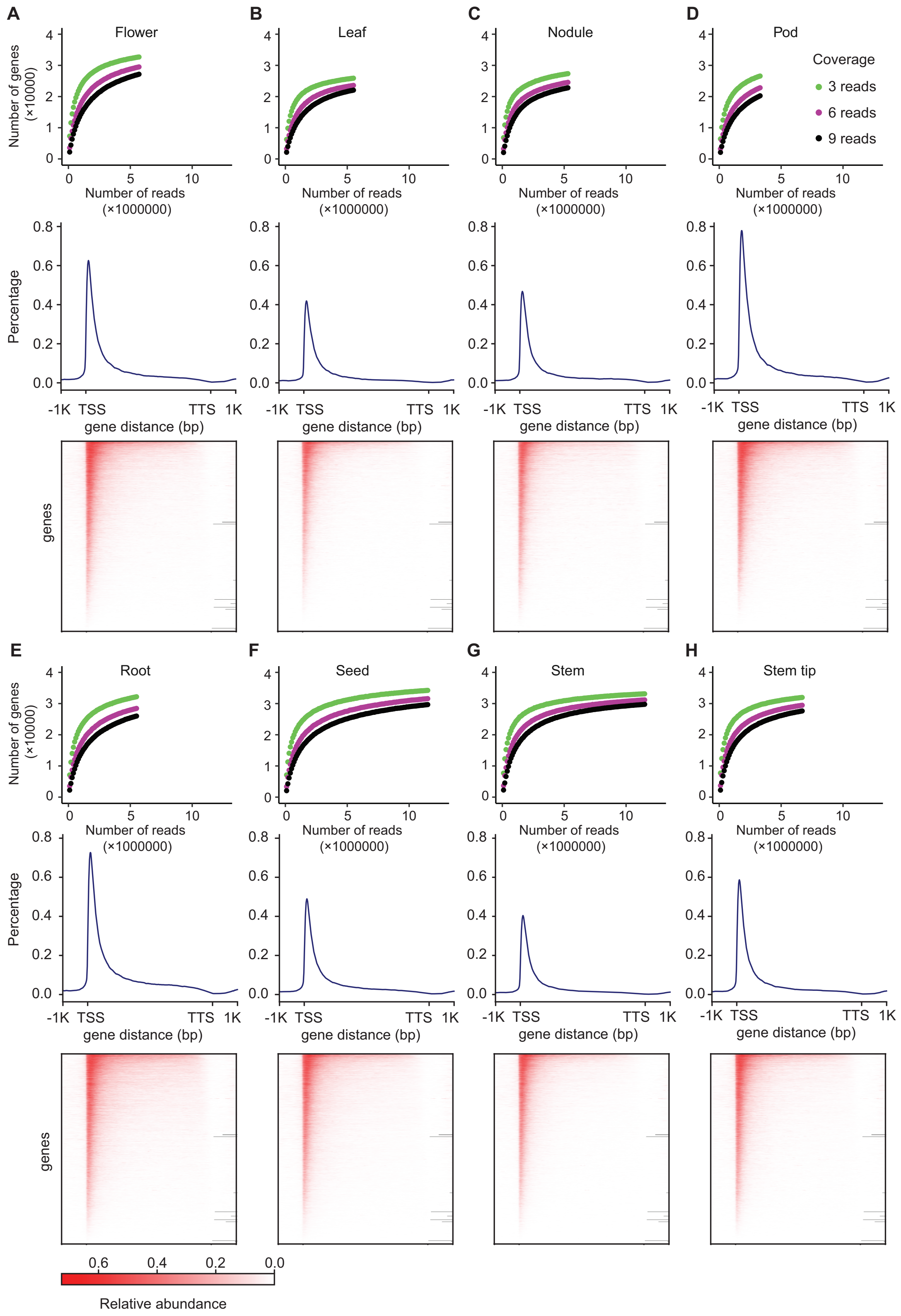

A
Flower
B
Leaf
C
Nodule
D
Pod
Coverage
3 reads
6 reads
9 reads
Number of genes (×10000)
Number of reads (×1000000)
Percentage
-1K TSS TTS 1K
gene distance (bp)
genes
E
Root
F
Seed
G
Stem
H
Stem tip
Relative abundance

Supplementary Figure S1. Evaluation of the quality of sequencing and transcription start region (TSR) detection using STRIPE-seq (supports Figure 1). A-H Upper panel, the sequencing saturation analysis of TSR mapping in each tissue. The *y*-axis represents the number of covered genes at a given number of reads (*x*-axis). Middle panel, TSR distribution in the regions from 1 kb upstream of the annotated TSS and 1 kb downstream of the transcription termination site (TTS). Bottom panel, heatmap of read distribution on separate genes in the above regions. The color bar represents the relative abundance of coverage. The tissues include Flower (**A**), Leaf (**B**), Nodule (**C**), Pod (**D**), Root (**E**), Seed (**F**), Stem (**G**), and Stem tip (**H**).

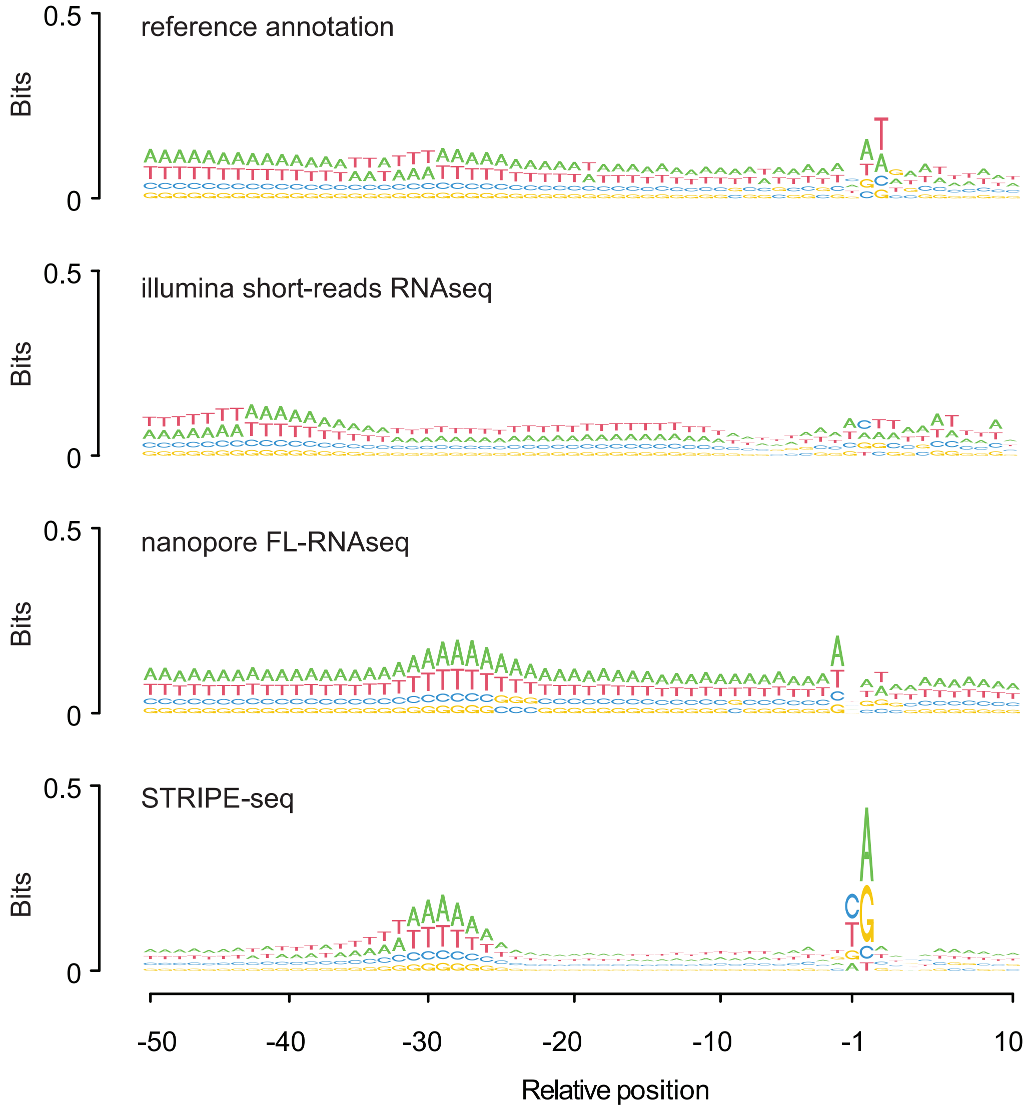

Supplementary Figure S2. Sequence patterns of transcription start sites (TSSs) in different annotation datasets (supports Figure 1). The *x*-axis represents 50 bp upstream and ten bp downstream of the annotated TSS from the reference annotation, *de novo* assembly RNAseq and Nanopore full length-RNAseq (FL-RNAseq), and detected in STRIPE-seq. The TSS was defined as the peak site of the TSR.

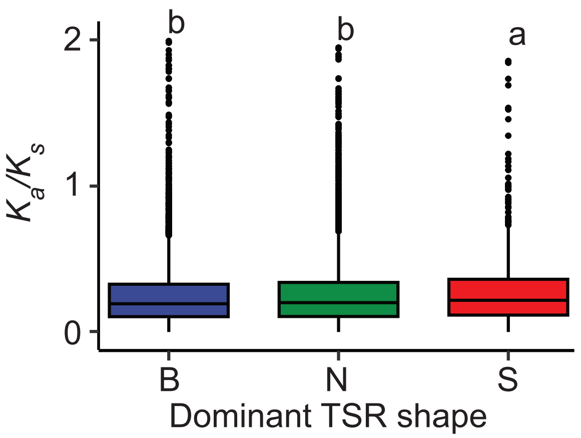

Supplementary Figure S3. The nonsynonymous substitution (K_a)/synonymous substitution (K_s) ratios in genes with different dominant transcription start region (TSR) shapes in maize (supports Figure 2). the letters on the x-axis represent the different TSR shapes. “B”, “N”, and “S” represent “Broad”-, “Narrow”-, and “Single-based”- shaped TSRs, respectively. Statistical analysis was performed using one-way ANOVA with multiple comparisons, and significant differences are indicated by letters on the plot. In each box plot across all panels, the box borders represent the first and third quartiles, the center line indicates the median, and the whiskers extend to 1.5 times the interquartile range beyond the quartiles. Dots represent outliers.

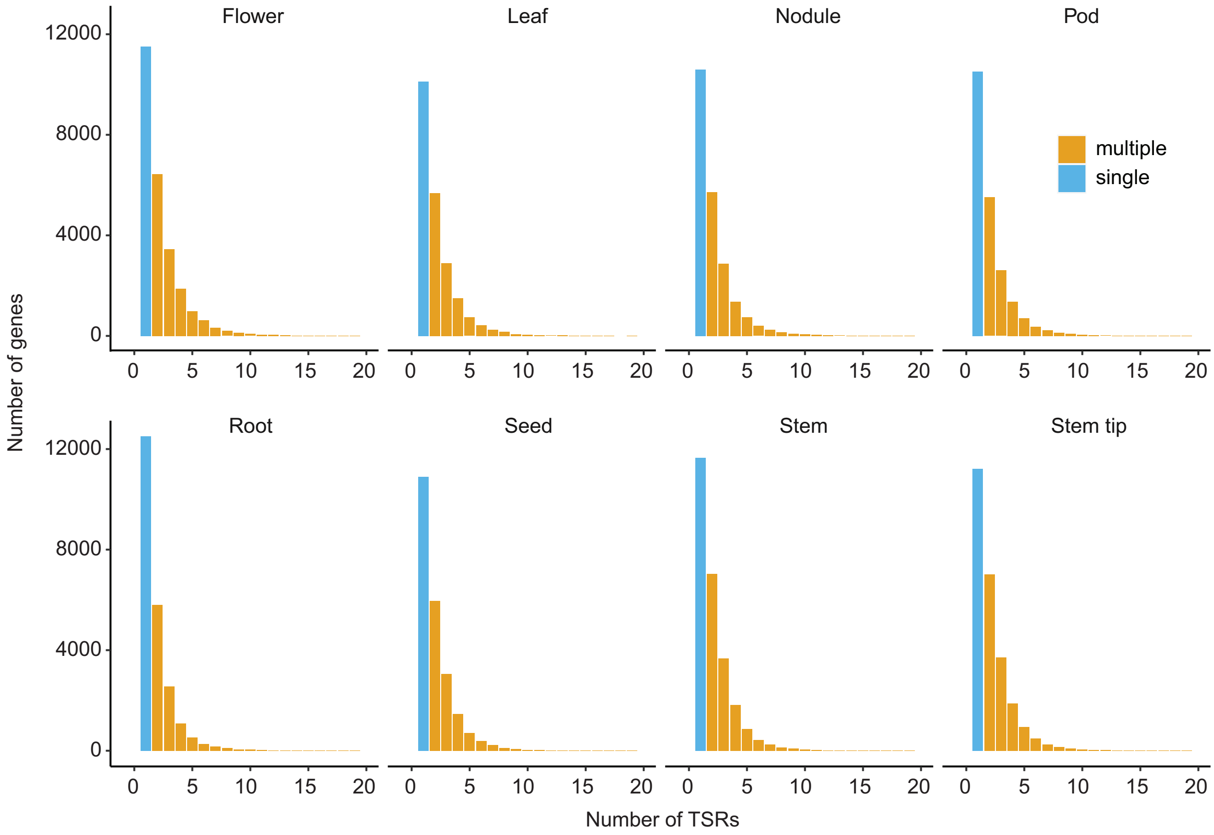

Supplementary Figure S4. Statistics of the number of genes with a given number of transcription start regions (TSRs) in each tissue (supports Figure 2). The *y*-axis represents the number of genes. The *x*-axis represents the number of TSRs. The blue bars represent single TSR genes. The orange bars represent multiple TSR genes.

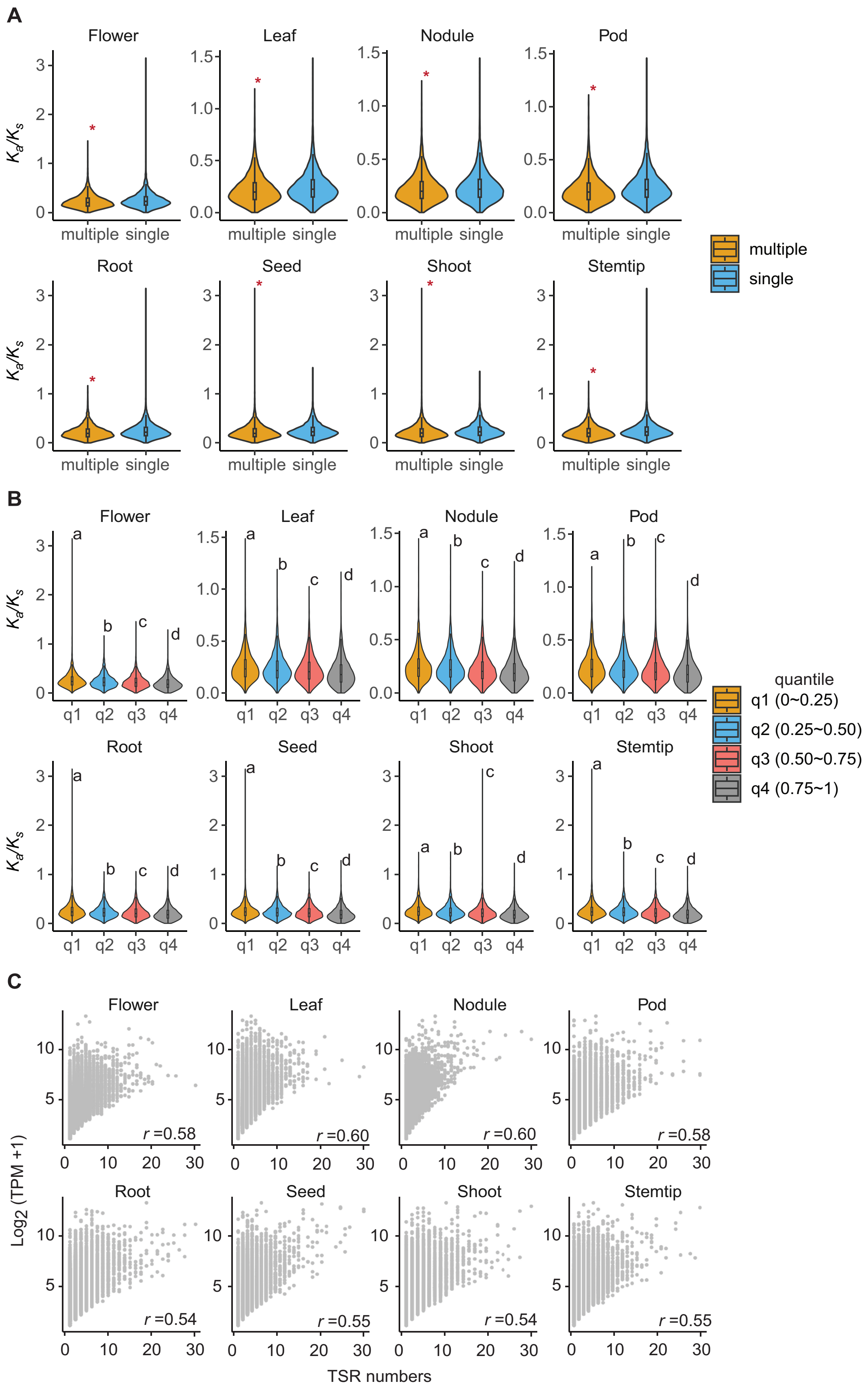

A
Flower
Leaf
Nodule
Pod
Root
Seed
Shoot
Stemtip
Ka/Ks
multiple single
multiple
single
B
q1 q2 q3 q4
quantile
q1 (0~0.25)
q2 (0.25~0.50)
q3 (0.50~0.75)
q4 (0.75~1)
C
r =0.58
r =0.60
r =0.60
r =0.58
r =0.54
r =0.55
r =0.54
r =0.55
Log2 (TPM +1)
TSR numbers

Supplementary Figure S5. Interplays between gene expression levels, transcription start region (TSR) numbers, and intensities of purifying selection (supports Figure 2). K_a/K_s ratios of genes with single TSR versus those with multiple TSRs (**A**) and at different levels of expression (**B**), and positive correlations between TSR numbers and levels of gene expression (**C**) in each of the eight tissues. "Single" and "multiple" represent genes with single TSR and multiple TSRs, respectively. Genes were divided into four categories (from q1-lowest expression to q4 -highest expression) based on the quartiles of expression levels in each tissue. Statistical analysis was conducted using one-way ANOVA with multiple comparisons, with significant differences indicated by different letters on the plot. KS test was used to assess statistical significance (*$p < 0.01$). In each box plot across all panels, the box borders represent the first and third quartiles, the center line indicates the median, and the whiskers extend to 1.5 times the interquartile range beyond the quartiles. Dots represent outliers.

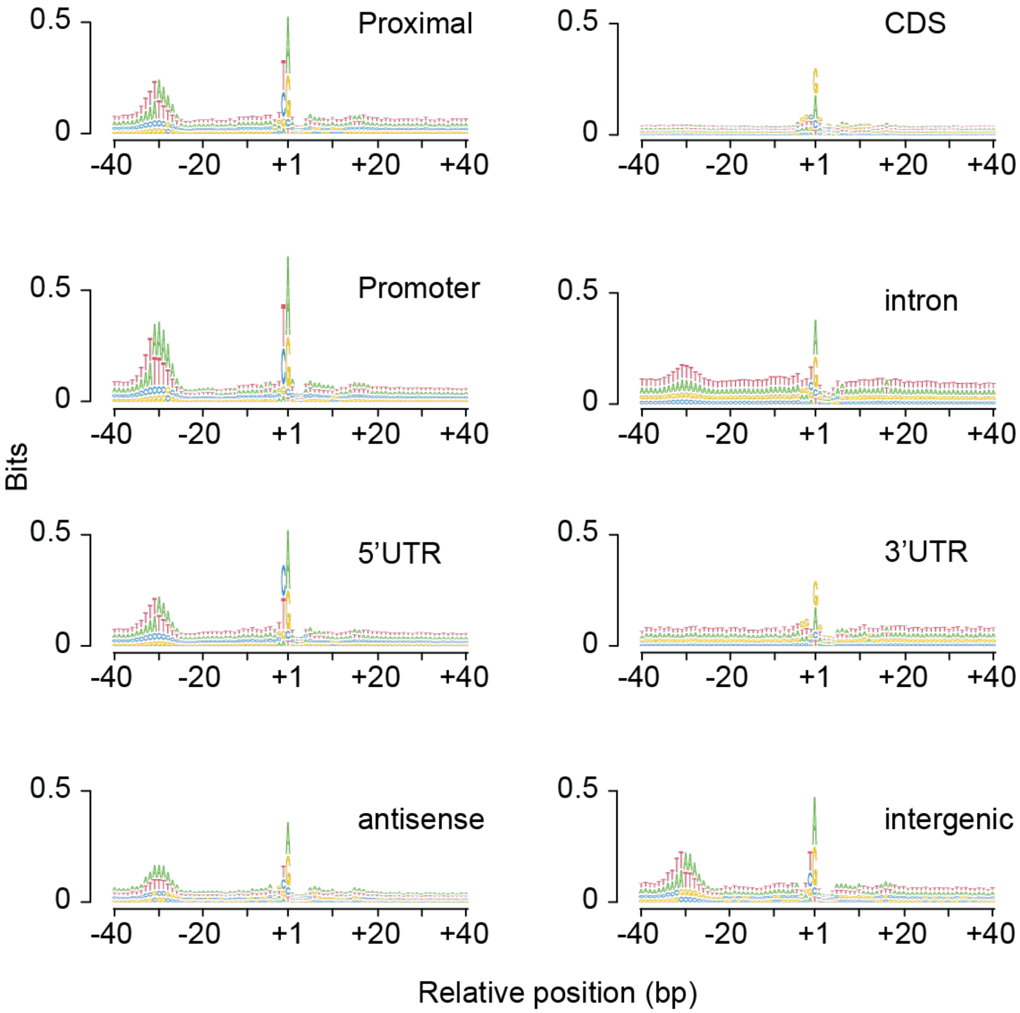

Supplementary Figure S6. Sequence patterns around transcription start regions (TSRs) in different feature regions (supports Figure 2). The TSS was defined as the peak site of the TSR. The feature regions were extracted from the reference annotation files. TATA-box elements were detected at 30 bp upstream of TSRs in the canonical regulatory and antisense, and intergenic regions. Different from the “TATA-box elements” detected in other regions, “TATA-box elements” in introns were barely discernible when the enriched ‘T"s in the surrounding were considered. The TATA-box elements were not detected around C-TSRs and 3'untranslated region (UTR)-TSRs.

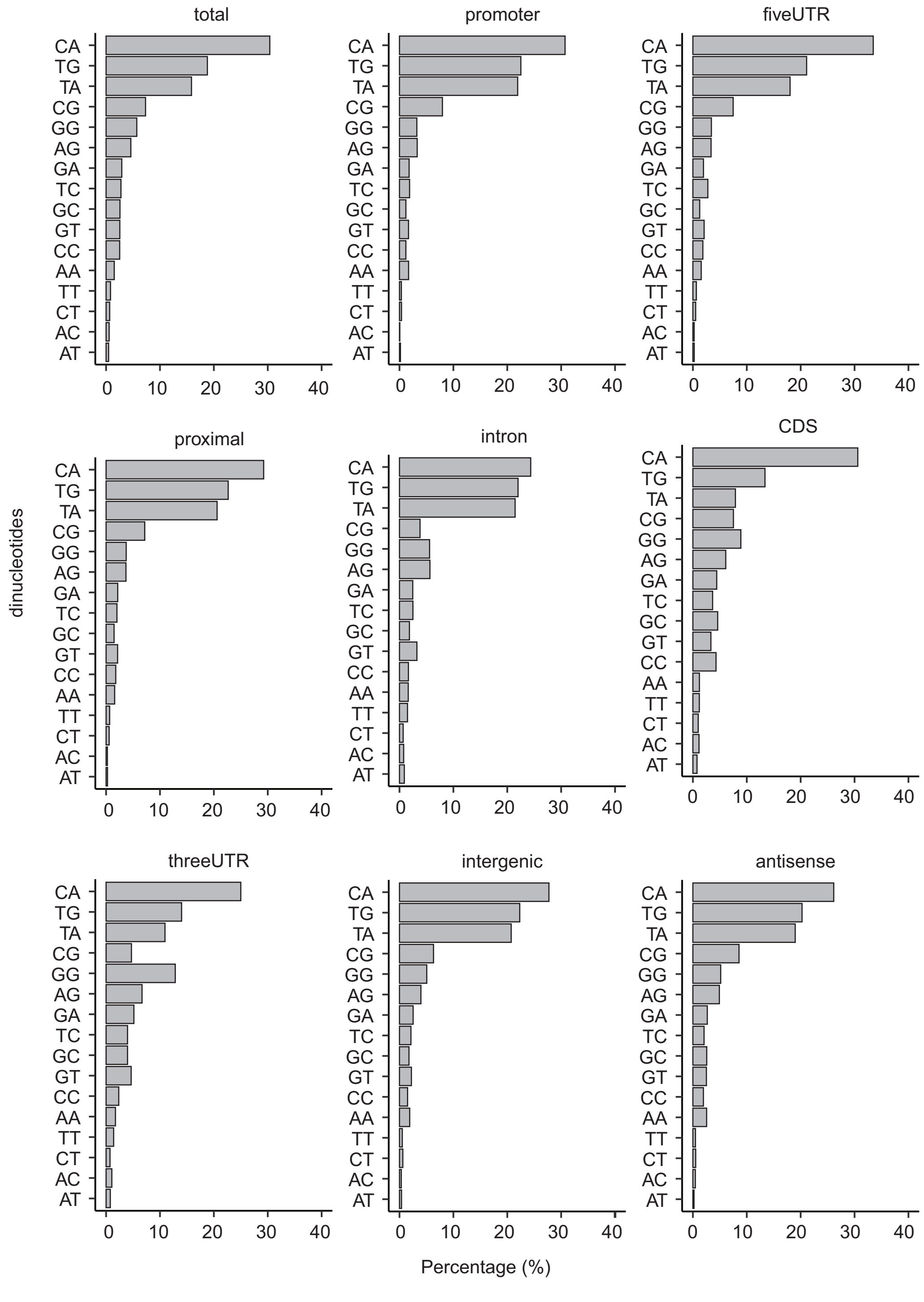

Supplementary Figure S7. The dinucleotide pattern of transcription start regions (TSRs) in each feature region (supports Figure 2). The *y*-axis represents the category of dinucleotides. The *x*-axis represents the percentage of TSRs in each category. The feature regions were extracted from the reference annotation files. UTR and CDS represent untranslated region and coding sequence, respectively.

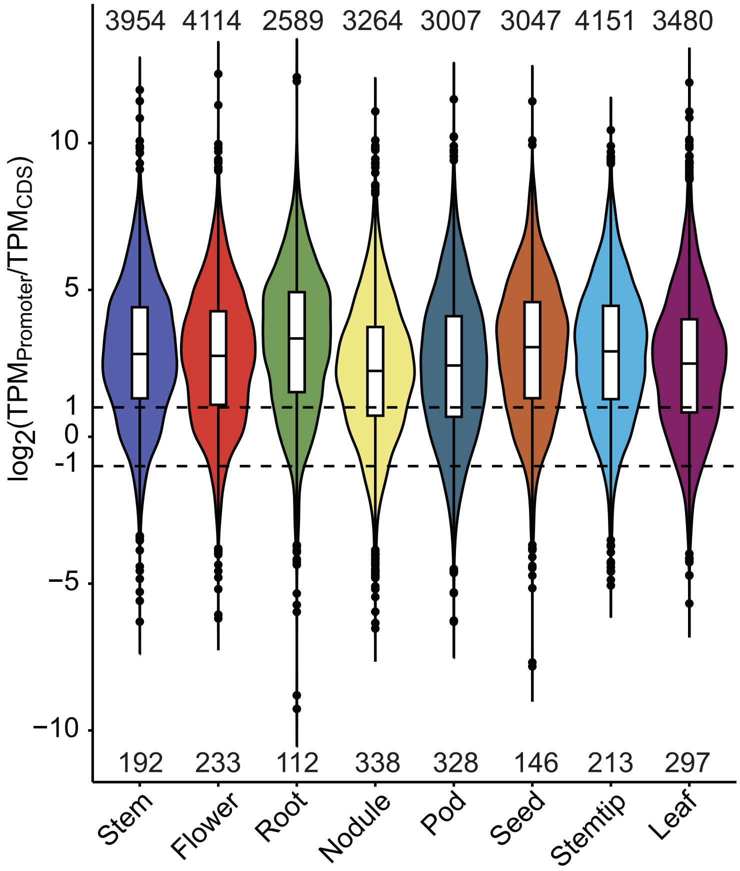

Supplementary Figure S8. Comparison of expression levels of promoter-TSRs and CDS-TSRs for specific genes with both categories of TSRs identified in each of the eight tissues (supports Figure 2). The *y*-axis represents the log2-transformed fold change in expression levels between P-TSRs and C-TSRs. Dotted lines at 1 and -1 indicate a two-fold change threshold. The numbers above and below the plot represent the number of genes with relatively higher expression of promoter (P)-TSRs and CDS (C)-TSRs, respectively. In each box plot across all panels, the box borders represent the first and third quartiles, the center line indicates the median, and the whiskers extend to 1.5 times the interquartile range beyond the quartiles. Dots represent outliers.

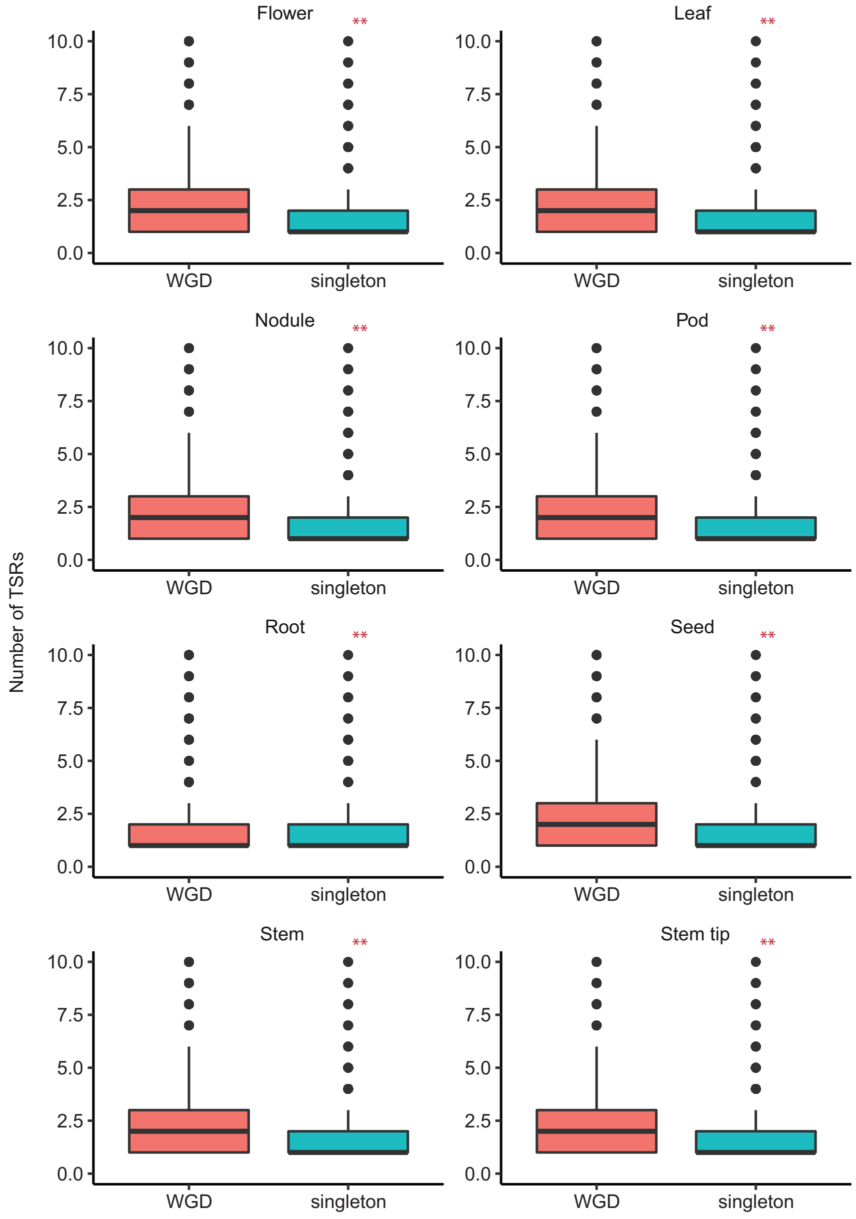

Supplementary Figure S9. The number of transcription start regions (TSRs) in whole genome duplication (WGD) and singleton gene groups in each tissue (supports Figure 3). The tissues include Flower, Leaf, Nodule, Pod, Root, Seed, Stem, and Stem tip. Kolmogorov–Smirnov (KS) test was performed to assess statistical significance (** p value < 0.01). In each box plot across all panels, the box borders represent the first and third quartiles, the center line indicates the median, and the whiskers extend to 1.5 times the interquartile range beyond the quartiles. Dots represent outliers.

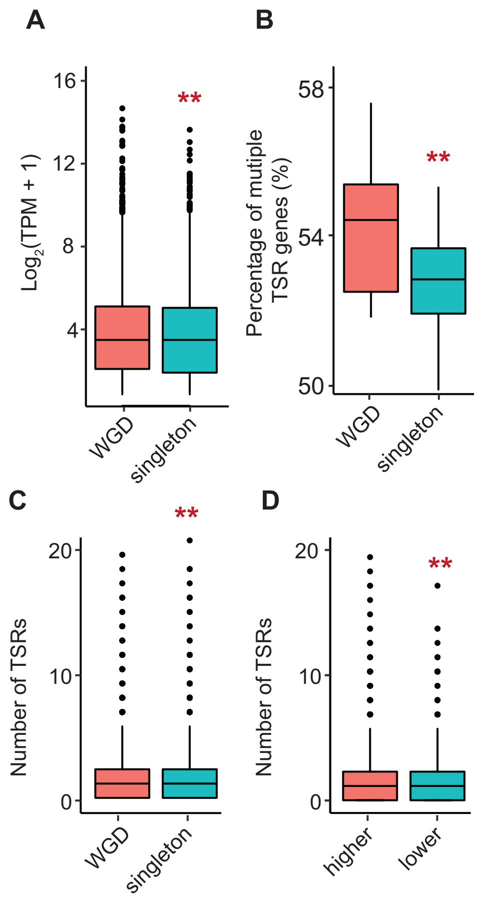

Supplementary Figure S10. The characteristics of transcription start region (TSR) divergence after whole-genome duplication in maize (supports Figure 3). (A) The expression abundance in each gene group. The expression abundances were normalized by transcripts per million (TPM) and log2 transformed. **(B)** The percentage of multiple TSR genes in each gene group. **(C)** The number of TSRs in one gene in each gene group. **(D)** The number of TSRs in one gene in high-expressed WGD copy and lower-expressed WGD copy. *KS* test was performed to assess statistical significance (***p* value < 0.01). In each box plot across all panels, the box borders represent the first and third quartiles, the center line indicates the median, and the whiskers extend to 1.5 times the interquartile range beyond the quartiles. Dots represent outliers.

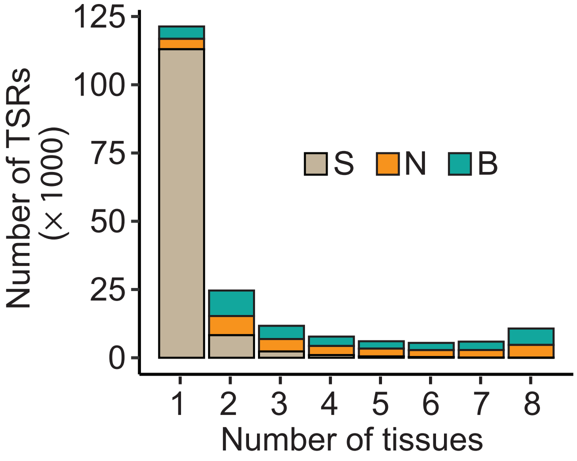

Supplementary Figure S11. The relationship between transcription start region (TSR) shape and the number of TSRs present in a given number of tissues (supports Figure 4). Different colors represent the different TSR shapes. “B”, “N”, and “S” represent “Broad”-, “Narrow”-, and “Single-based”- shaped TSRs, respectively. The number in the axis represent the TSRs detected in how many tissues.

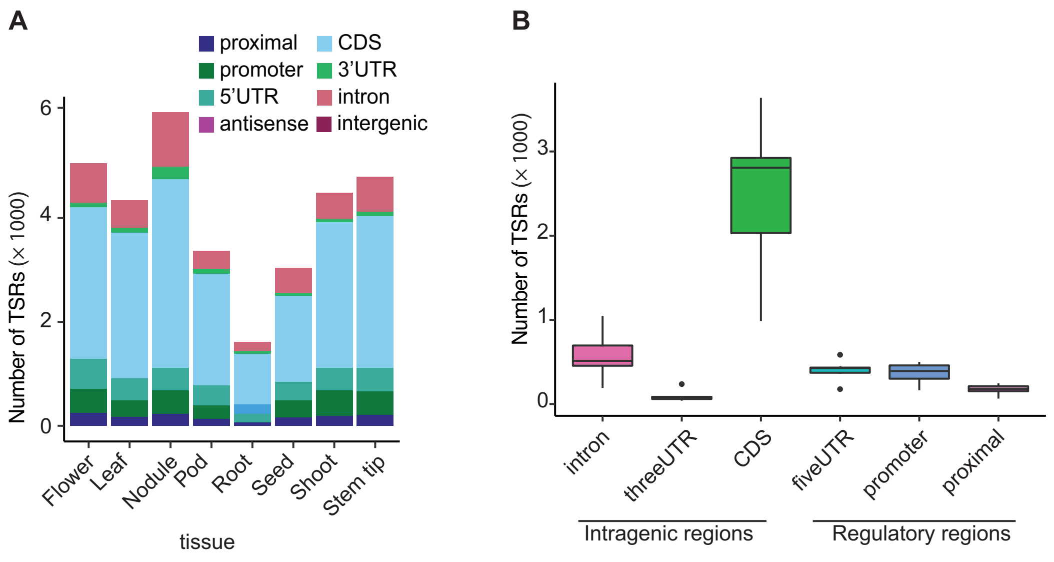

Supplementary Figure S12. The number of tissue-specific transcription start regions (TSRs) in each tissue and each genic region (supports Figure 4). (A) The number of tissue-specific TSRs present in a given tissue. Different colors represent different annotated features. (B) The number of tissue-specific TSRs present in each genomic feature. The intragenic regions included introns, 3'UTRs (3'untranslated regions) and coding sequences (CDSs). The regulatory regions included 5'UTR, promoter and proximal regions. In each box plot across all panels, the box borders represent the first and third quartiles, the center line indicates the median, and the whiskers extend to 1.5 times the interquartile range beyond the quartiles. Dots represent outliers.

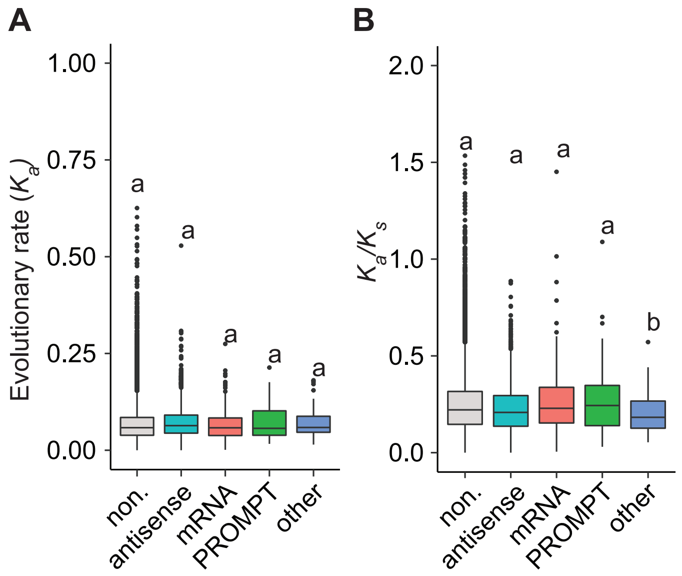

Supplementary Figure S13. The evolutionary rate (K_a) and K_a/K_s in genes with different transcription start region (TSR) categories (supports Figure 5). "non." represents the genes without antisense- and bidirectional TSRs. "antisense", "mRNA", "promoter upstream transcript (PROMPT)" and "other" represent bidirectional TSRs belonging to the "antisense-TSR", "mRNA-mRNA", "mRNA-PROMPT", and "other-divergent" categories, respectively. Statistical analysis was performed using one-way ANOVA with multiple comparisons, and significant differences are indicated by letters on the plot. In each box plot across all panels, the box borders represent the first and third quartiles, the center line indicates the median, and the whiskers extend to 1.5 times the interquartile range beyond the quartiles. Dots represent outliers.

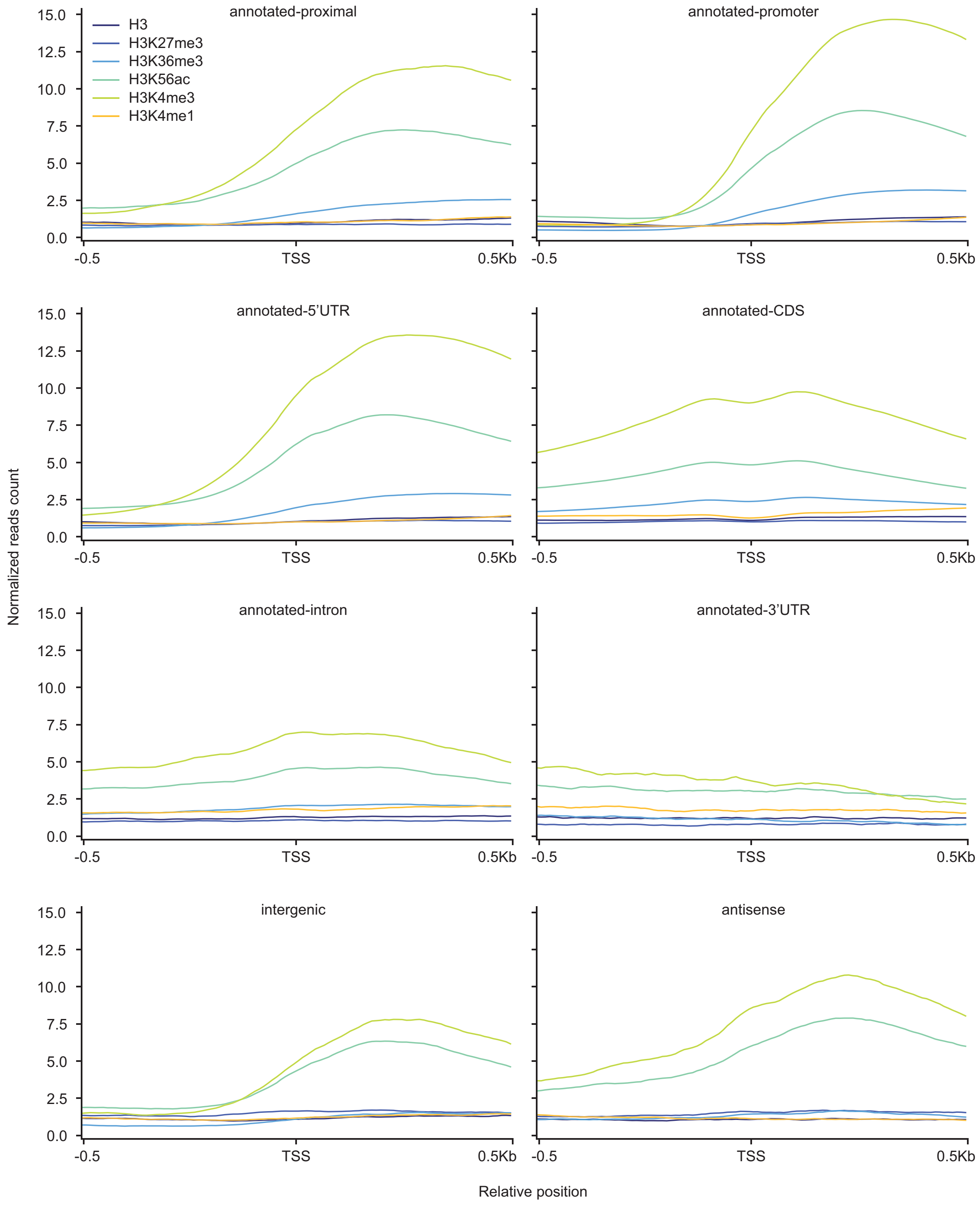

Supplementary Figure S14. The distributions of histone modifications and variants around transcription start regions (TSRs) located in a given annotated feature in leaves (supports Figure 6). The TSS was defined as the peak site of the TSR. The lines with different colors represent different markers. The feature regions were extracted from reference annotation files.

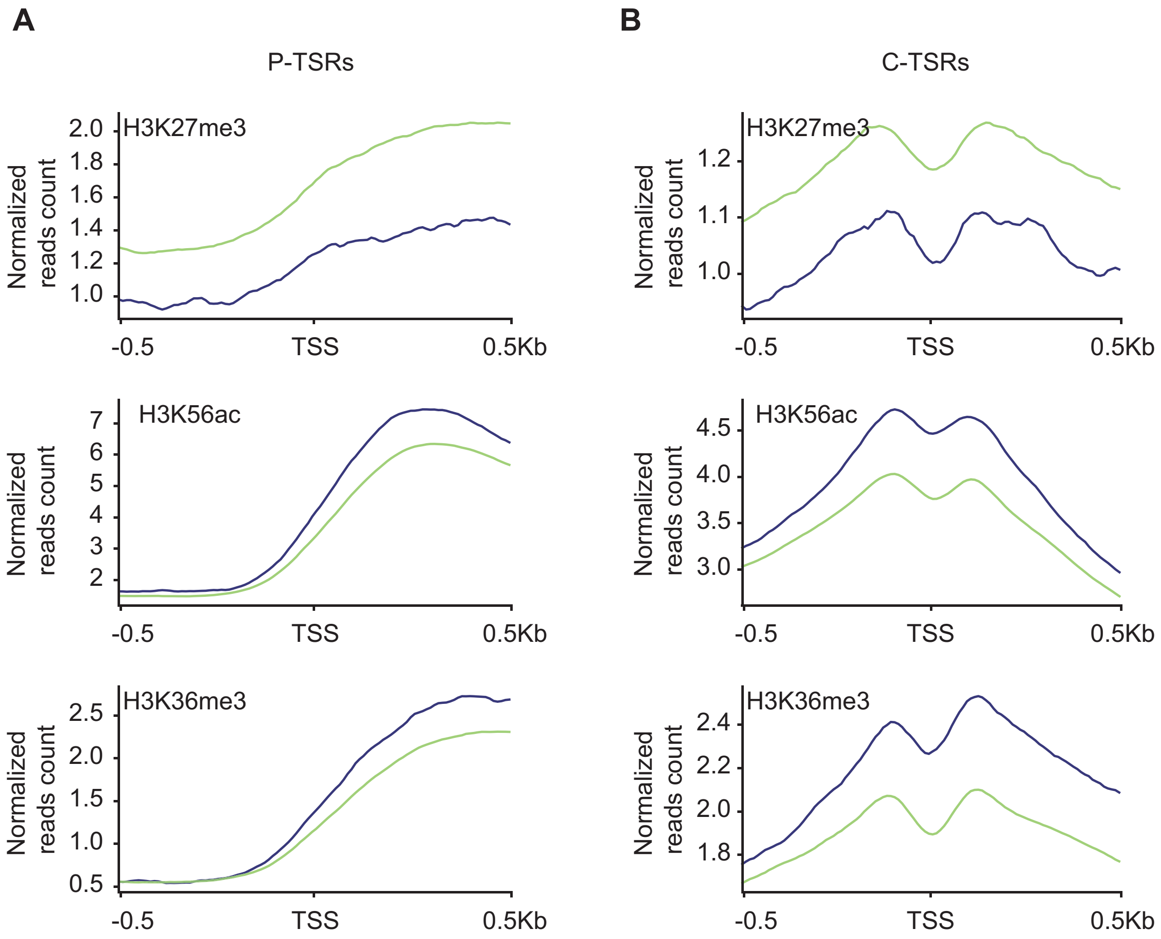

Supplementary Figure S15. The distributions of histone modifications around the leaf-specific transcription start regions (TSRs) (LsTSRs) and the TSRs specific to each of the other seven tissues (OsTSRs) located in given features in leaves (supports Figure 6). **(A)** The distribution of markers around TSSs located in annotated promoter regions. (**B**) The distribution of markers around TSSs located in annotated CDS regions. The TSS is defined as the peak of the TSR. The blue and green lines represent the LsTSRs and OsTSRs, respectively. CDS (C)-TSRs and promoter (P)-TSRs represent TSRs and the TSRs in CDS and promoter regions, respectively.

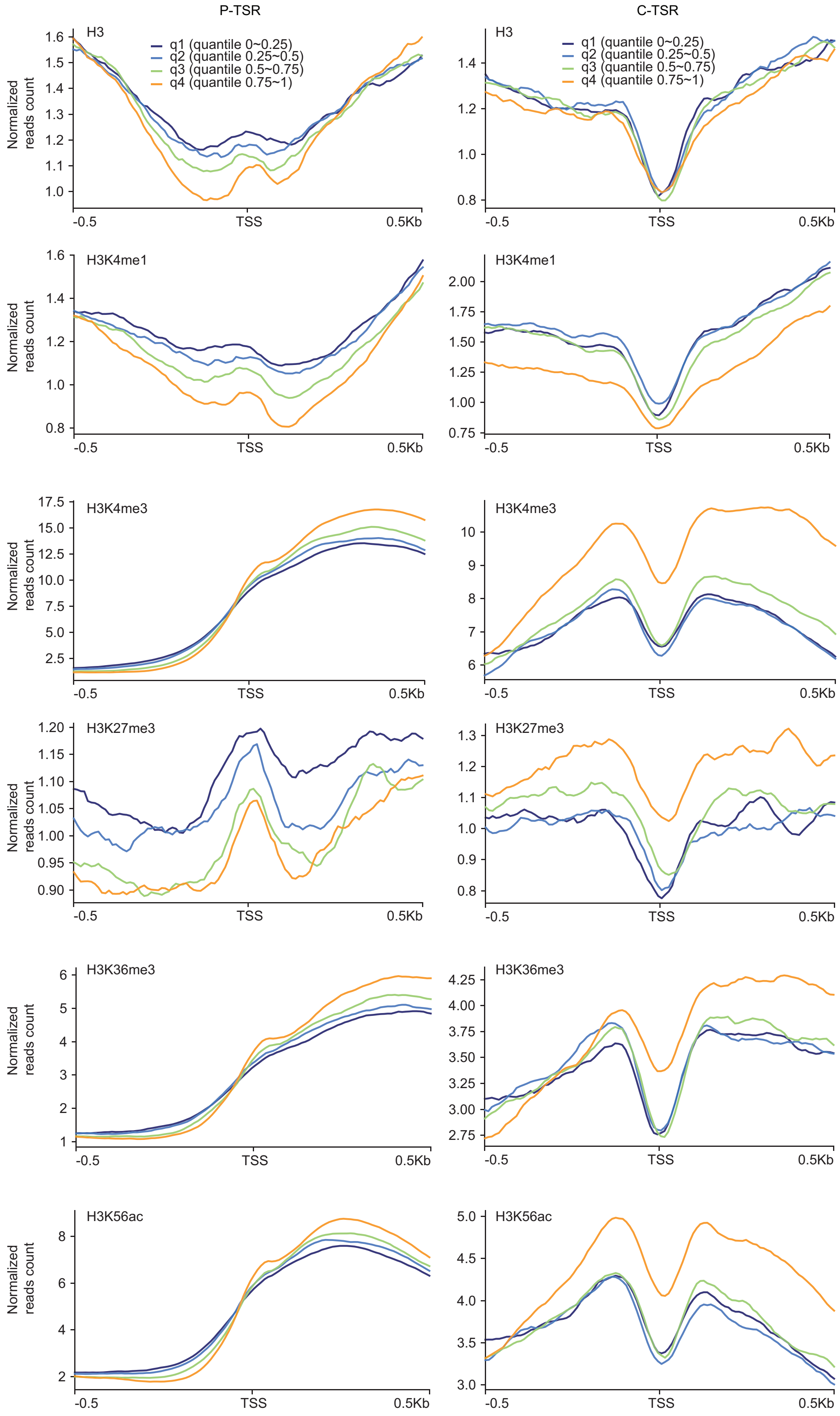

P-TSR
C-TSR
H3
H3K4me1
H3K4me3
H3K27me3
H3K36me3
H3K56ac
q1 (quantile 0~0.25)
q2 (quantile 0.25~0.5)
q3 (quantile 0.5~0.75)
q4 (quantile 0.75~1)
Normalized reads count
-0.5
TSS
0.5Kb

Supplementary Figure S16. Distributions of histone modifications around transcription start regions (TSRs) expressed at different levels in the leaf tissue (supports Figure 6). Gene were divided into four categories (from q1 to q4) based on quartiles of expression levels in each tissue. The left panels show the distribution of histone markers around promoter (P)-TSRs, while the right panels display the distribution of histone markers around CDS (C)-TSRs.

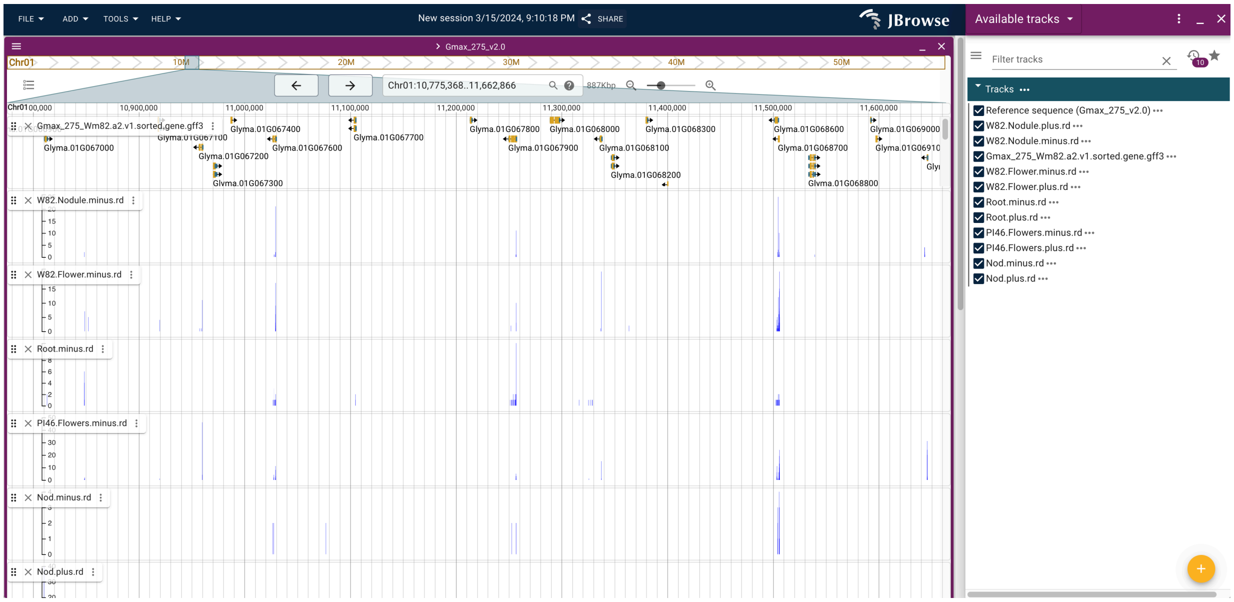

Supplementary Figure S17. Screenshot showcasing the transcription start sites (TSSs) on the genome browser webpage (supports Figure 1). The link is http://xtlab.hzau.edu.cn/jbrowser.

Supplementary Table S1. The statistical summary information of the read number in each process step.

Tissues	Number of cleaned reads	Number of reads with UMI structure[a]	Number of reads after removed PCR redundance[a]	Number of reads after rRNA removal[a]	Number of reads mapped to the reference genome[a]
Flower	64,195,334	59,856,957 (93.24%)	15,159,334 (23.61%)	7,773,195 (12.11%)	5,755,820 (8.97%)
Leaf	89,727,033	83,145,551 (92.66%)	12,810,163 (14.28%)	9,487,795 (10.57%)	5,501,584 (6.13%)
Nodule	75,827,628	68,909,667 (90.88%)	16,589,416 (21.88%)	9,088,735 (11.99%)	5,376,021 (7.09%)
Pod	90,403,425	83,889,035 (92.79%)	16,329,046 (18.06%)	5,490,927 (6.07%)	3,344,139 (3.70%)
Root	53,670,000	50,088,105 (93.33%)	16,987,584 (31.65%)	7,041,255 (13.12%)	5,510,135 (10.27%)
Seed	119,273,897	110,093,605 (92.30%)	26,559,094 (22.27%)	15,923,148 (13.35%)	11,539,813 (9.68%)
Stem	90,700,186	83,713,683 (92.30%)	19,862,120 (21.90%)	15,471,060 (17.06%)	11,516,039 (12.70%)

[a]Percentages of reads in cleaned reads are shown in parentheses.

Supplementary Table S2. Oligos used for constructing the STRIPE-seq libraries.

Name	Sequence[a]
RTO_2 (reverse transcription oligo_2)	CAAGCAGAAGACGGCATACGAGATACATCGGTGACTGGAGTTCAGACGTGTGCTCTTCCGATCTNNNNNN
RTO_4	CAAGCAGAAGACGGCATACGAGATTGGTCAGTGACTGGAGTTCAGACGTGTGCTCTTCCGATCTNNNNNN
RTO_5	CAAGCAGAAGACGGCATACGAGATCACTGTGTGACTGGAGTTCAGACGTGTGCTCTTCCGATCTNNNNNN
RTO_6	CAAGCAGAAGACGGCATACGAGATATTGGCGTGACTGGAGTTCAGACGTGTGCTCTTCCGATCTNNNNNN
RTO_7	CAAGCAGAAGACGGCATACGAGATGATCTGGTGACTGGAGTTCAGACGTGTGCTCTTCCGATCTNNNNNN
RTO_12	CAAGCAGAAGACGGCATACGAGATTACAAGGTGACTGGAGTTCAGACGTGTGCTCTTCCGATCTNNNNNN
TSO (template switching oligo)	/5Biosg/CCTACACGACGCTCTTCCGATCTNNNNNNNNTATArGrGrG
FLO (forward library oligo)	AATGATACGGCGACCACCGAGATCTACACTCTTTCCCTACACGACGCTCTTCCG

[a]/5Biosg/ is IDT code for 5' biotin, and rG is IDT code for ribo-G.

Supplementary Table S3. Impact of transcription start sites (TSS) abundance thresholds on transcription start region (TSR) definition.

TPM cutoff	Total TSR numbers	Width[a,b] ≥ 1bp	Width[a,b] ≥ 10bp	Width[a,b] ≥ 100bp	Width[a,b] ≥ 1000bp
0	656,075	492,084 (75.00%)	148,200 (22.59%)	15,781 (2.41%)	10 (0.00%)
1	193,579	151,172 (78.09%)	40,448 (20.89%)	1,959 (1.01%)	0 (0.00%)
5	42,410	34,941 (82.39%)	7,410 (17.47%)	59 (0.14%)	0 (0.00%)
10	20,015	17,269 (86.28%)	2,736 (13.67%)	10 (0.05%)	0 (0.00%)

[a]Lengths of TSRs.

[b]Pencentages pf total numbers of TSRs.

Supplementary Table S4. The sources of datasets used in this study.

Type	Tissue	SRAID	Sources
STRIPE-seq	Stem tip	SRR15601585	In this study (PRJNA757465 & PRJNA757638)
STRIPE-seq	Seed	SRR15601586	
STRIPE-seq	Leaf	SRR15601584	
STRIPE-seq	Pod	SRR15601587	
STRIPE-seq	Root	SRR15601589	
STRIPE-seq	Stem	SRR15601591	
STRIPE-seq	Nodule	SRR15601588	
STRIPE-seq	Flower	SRR15601590	
RNA-seq	Root	SRR15607172	
RNA-seq	Nodule	SRR15607173	
ChIP-seq for H3	Leaf	SRR8742348	Lu et al. 2019
ChIP-seq for H3K27me3	Leaf	SRR8742349	
ChIP-seq for H3K36me3	Leaf	SRR8742350	
ChIP-seq for H3K56ac	Leaf	SRR8742351	
ChIP-seq for H3K4me3	Leaf	SRR8742352	
ChIP-seq for H3K4me1	Leaf	SRR8742353	
