# Supplementary material for: Noncanonical transcription initiation is primarily tissue specific and epigenetically tuned in paleopolyploid plants
Source: Plant Cell. 2024 Nov 14;37(1):koae288. doi: 10.1093/plcell/koae288 (PMC11663555; doi:10.1093/plcell/koae288)
Supplement: koae288_Supplementary_Data [file tpc.11198.2024-s11.pdf › tpc.11198.2024-s11.pdf]

THE PLANT CELL

Non-canonical Alternative Transcription Initiation is Prominently Tissue-Specific, Evolutionarily Shaped, and Epigenetically Tuned in Paleopolyploid Plants

Xutong Wang, Jingbo Duan, Chancelor Clark, Wanjie Feng, and Jianxin Ma

Review Timeline:

Submission Date:	03-Apr-2024
Editorial Decision:	06-May-2024
Resubmission Received:	12-Aug-2024
Editorial Decision:	06-Sep-2024
Revision Received:	12-Sep-2024
Editorial Decision:	24-Sep-2024
Revision Received:	26-Sep-2024
Accepted:	30-Sep-2024

Dr. Jianxin Ma
Purdue University
West Lafayette, Indiana 47907

Dear Dr. Ma:

Thank you for choosing to send your manuscript entitled "Widespread transcription initiation within coding sequences marks tissue identity and accessible chromatin in soybean" for consideration at The Plant Cell. Your submission has been evaluated by members of the editorial board as well as expert reviewers in your field, and we regret to inform you that we are not able to recommend the publication of this manuscript in its current form. We have had input from multiple scientists and we have solicited post-review comments.

During the post-review consultation session, we also agreed that if you could address the major points raised by the reviewers by new experiments, we would welcome a resubmission. This may be treated as a new submission, but we would attempt to use at least some of the same reviewers. Nevertheless, reviewers will be asked to assess as a new manuscript (i.e. are the claims fully supported by the data and do the results presented move the field forward?), and not only whether previous reviewer comments have been addressed.

While the reviewers raised several important issues that must be considered in a resubmission, there was a general consensus that the manuscript is lacking key information necessary to support many of the conclusions. Examples include parameter selection for many of the analyses, quality control criteria, Ka/Ks values, gene models used when multiple transcripts are present, etc. The absence of code accessibility was also noted.

It will be important to convince the editors and reviewers that the study adds significant new understanding(s) of mechanisms or processes, or otherwise represents a significant advance in the field, and that the major claims made are fully justified by the data presented. This includes careful consideration and explanation of the various controls used in experiments, the extent and manner of replication, and the statistical analyses used. Sampling methods and the nature of "biological replicates" should be described precisely (i.e. different plants, parts of plants, pooled tissue, independent pools of tissue, sampled at different times, etc.), along with a clear description of and rationale for any statistical analyses conducted. The reader should know exactly what was sampled; what forms the basis of the calculation of any means and other statistical variables and parameters reported. This is also necessary to ensure that proper statistical analysis was conducted.

The Plant Cell now requires authors to complete and submit an author revisions checklist upon submission of a revised manuscript. The aim of the checklist is to aid authors in preparing a high-quality manuscript, facilitate the review and assessment of revised manuscripts, and help to ensure that journal standards are maintained across the board. If your manuscript is accepted, the completed checklist will be published as supplemental material attached to the article online. Please download a copy of the checklist (a fillable PDF form) at this link, for submission with your revised manuscript: https://tpc.msubmit.net/html/Author_Revisions_Checklist.pdf.

Note also that supplemental materials should be restricted to large datasets and tables, presentation of replicates, and validation of reagents, methods, or genotypes. Any data that are used to support the main claims must be in the main manuscript. Supplemental figure legends must indicate what figure in the main manuscript is supported by the supplemental data presented.

We thank you for your interest in and support of The Plant Cell. We hope we will be able to render a more positive decision on future work.

Sincerely,

The Plant Cell Editorial Board

(Please note that due to a potential conflict of interest, EIC Blake Meyers did not participate in evaluating this manuscript).

-- Reviewer comments:

Reviewer #1 (Comments for the Author):

I applaud the authors for a thorough and, in my view, much needed study of transcriptional initiation in plants.
There are a few points for consideration, however.

1) Reproducibility. From a bioinformatics perspective, it is always good to have code-based documentation of the data analysis. I would like to see this provided with the publication. A github repository, or equivalent, would do nicely. Just provide the scripts for read processing and mapping; Rscripts for the R analyses; etc., taking a user from raw data to analysis results.

2) Robustness. Comments on and consequences of parameter selection should be discussed. How do the results change for a higher threshold of TSSs kept or for clustering to define TSRs?

3) Accessibility. The data should make a valuable track on the SoyBase and Ensembl Plants genome browsers. If accepted for publication in TPC, I think this should be arranged.

4) Interpretation. The original STRIPE-seq publication noted a link of promoter-distal TSRs with enhancer RNAs in human samples. That publication stated " a fraction of these sites are highly enriched for H3K4me1 and H3K27ac but not H3K4me3" whereas in this study the finding is "H3K4me1, a marker of RNA polymerase II elongation, is underrepresented in TSRs within CDS." I think these associations warrant further inspection and comment.

Reviewer #2 (Comments for the Author):

In the submitted manuscript, Wang and colleagues describe a study on using Survey of TRanscription Initiation at Promoter Elements with high-throughput sequencing (STRIPE-seq) to annotate the Transcription Start Sites (TSSs) in the soybean reference genome. The STRIPE-seq as a technique was described in a previous publication (Policastro et al., 2020) applied to yeast and the human genome. In the manuscript it is applied to a plant genome for the first time.
Characterization of transcription initiation using high-throughput sequencing methods has been performed for plant genomes including Arabidopsis thaliana, and Zea maize. The main barrier for the adoption of regular TSSs profiling for genome annotation is that the different protocols available are quite laborious and in need of large amounts (nanograms) of starting material, when compared to STRIPE-seq. Modifications to the method to be used in plants will be appreciated by the community.
For this study the authors generated TSSs annotations from Williams 84, the Soybean cultivar used as reference plant, from eight different tissues. Identified TSSs were clustered in regions (TSRs - groups of TSSs) and assigned to annotated genes based on distance and strand information. In addition to comparison against the reference genome the authors compared against a companion dataset of RNA-seq that was generated from the same tissues and used published ChIP-seq data from different histone marks (Lue et al., 2019) generated from leaf tissue for further contextualization of the data.

1. QC of the data, the authors presented a saturation analysis, but was only mentioned and never properly described nor discussed. Some further claims about method sensitivity, accuracy, sufficiency, etc., are mentioned often with proper description/discussion of the methods or the results. For instance, the authors evaluated accuracy, but we don't know what they found as results aren't shared with the reader, at least not explicitly.
2. The study identified TSSs for ~72% of the current annotated genes with variation across the tissues. The authors share a distribution, but how many genes in the current genome were wrongly annotated? The authors explore the data at the gene level, however the identification of multiple TSSs and TSRs should be correlated with isoforms/transcripts annotated instead of being collapsed at the gene level. At the very least the authors should include this information in the paper and focus on gene features when more relevant to the analysis
3. Characterization of the TSRs according to how spread is the transcription initiation across different TSSs for a given gene - i.e., the promoter shape. The authors found that single nucleotide (S-shaped) was the prevalent shape of TSRs in Soybean and that more than half of the genes have more than one TSR. Did you report the number of genes with more than one type of shaped TSR?
4. The authors studied if different TSRs shapes were correlated with different genic features, such as expression as has been previously reported. They identified genes with S-shaped TSRs to be low expressed when contrasting the distribution, however beyond the plotting of the box-plot there is not statistically evaluation of the differences between distributions neither explanation of which dataset was used to derive the gene expression values (STRIPE-seq TPM? RNA-seq TPM?).
5. In the discussion, the statement that claims that predominant S-shaped TSRs in the maize genome are not associated with low expressed genes is wrong. The Mejia-Guerra et al., 2015 paper specifically evaluated the correlation between shape and expression levels and found that "the median expression of genes with broad TCs is higher than the median expression of genes with sharp TCs". The figures 2C and 2D (Mejia-Guerra et al., 2015) show CAGE expression and RNA-seq expression.
6. The authors explored differences between the Ka/Ks ratio between Soybean and common bean orthologs genes across the different TSRs shapes. The authors claimed that S-shaped TSRs have undergone lower intensities of purifying selection in the Soybean genome. The hypothesis is interesting, however there is no test to support the claim that the Ka/Ks ratio is `actually different` from what is observed in genes with a different shape. Some type of measurement of the extent of this difference is missing to support the claim. There is a lot of data in plants genome duplication, exploring this hypothesis beyond Soybean using some of the TSRs profiles for other plant genomes might be beyond the scope of this project but is an interesting avenue to explore.
7. An "strikingly" observation was the identification of alternative transcription initiation overlapping with intragenic regions, in particular with annotated coding sequences (CDS) for a large number of genes. I did not understand why this seems to be

unexpected? The authors mentioned that the intragenic TSRs were more frequent than the ones located within annotated canonical promoter regions. With what we know about the genome and transcriptions, the parsimonious hypothesis should be that the current annotation is missing a good portion of the shortest isoforms and that is why the TSRs overlap with regions other than the already annotated promoter and 5'UTRs.
8. The histone marks discussion is another half baked idea, the intention is good but the data is not sufficient to answer the question as the colocalization of the signals is not really enough for any conclusion. Some of the claimed mechanisms such as the "exhibit threshold effects on tissue-specific gene expression" don't have any support in the analysis so far.
9. What happened with the miRNA-seq data? It is not clear how it was used for the analysis.
10. As a large analysis paper that relies on bioinformatics analysis of many types, code availability is missing as GEO links and description of the methods is not sufficient to ensure reproducibility.

Reviewer #3 (Comments for the Author):

This manuscript is extensively analysing alternative initiation of transcription in soybean, in different tissues. They show quite a high % of the ATI is tissue-specific and that there are relatively many TSRs in coding sequences. These seem present in narrow NFRs. The MS provides many interesting correlations. It is a pity that none of the examples is investigated functionally. Now the data reported stays very descriptive. For example, what would the overexpression of a particular antisense RNA mean for the sense expression of a gene.
In the abstract they say there is little known how ATI evolves. This is however also not elaborately investigated in the paper, nor discussed. Similarly, the way ATI is regulated is not discussed.
The manuscript could be improved by discussing the aspects reported in the results also in the discussion. For example, what may be the relevance of WGD genes to be higher expressed and have more TSRs?
There are quite some typos here and there. In addition, the grammar and clarity of the text needs to be improved (I did not specify the locations of typos/unclarities).
Please realize that the histone modifications and H3 profile you have looked at is an integral part of gene regulation, including transcription initiation and elongation. It is not necessarily a major cause for ATI (which is suggested at various places in the text). One needs sequence-specific TFs and and/or general TFs to initiate transcription.

3-4. The title can be improved to cover the content of the paper better. Accessible chromatin data is for example taken from another paper and only represents one of the tissues examined. So I would not place this in the title.
49. you mention that canonical TSRs are located in the 5' upstream regulatory regions. I would say they are located in the core promoter. The 5' upstream regulatory regions can be located far away from the core promoter.
66. Soybean is also a valuable source of protein when thinking of the foreseen/needed transition from an animal-based to a plant-based diet to mitigate the climate crisis.
79. You mention epigenomic differentiation. I wonder if there is evidence showing that within one species duplicated genes are epigenetically regulated in a different manner. If not, I would leave this out. You also do not show this in this manuscript.
110. Figure 1c does not show that the nodule tissue exhibits the most unique TSRs compared with other tissue. is there another figure you can refer to?
122-124. To me it is unclear what is shown in figure 2b if I only read the legend. According to the legend the figure shows abundance, but give there are more S shaped TSRs, I would then expect the S shaped box to be higher on the Y-axis. The main text indicates you mean expression levels (RPM?? something else?), which clarifies the figure. Please clarify the text of the legend.
126-128. In Zhao et al they examine substitutions in the genes instead of the TSR, which you examine. Therefore the comparison you make is not entirely valid.
132-133: The text is unclear. if 80.1% contain multiple TSRs, the nr with fewer than 10 should be less than 80.1%.
142-143. It would be best to refer to Fig 1a if mentioning promoter and proximal. To be honest, to me proximal would logically refer to the proximal promoter, which would be the core promoter (called promoter by you), and the region upstream of this I would define as regulatory sequences. The term Canonical TSRs I would use for TSRs at the core promoter/5'UTR. Further upstream I would not refer to as canonical.
150-151. The intergenic regions can also contain regulatory sequences.
142-150. When adding up the % (50.4; 43.5; 3.6) they do not add up to 100%, where is the about 2.5% missing TSRs?
155-158. You mention TATA box elements were barely seen around intron TSRs, while they are around antisense TSRs. Both however show a similar sequence pattern in Fig S5, with T's being more prominent in introns and A's in antisense.
161. I See TA also enriched in introns (also intragenic). GG seems enriched in CDS and 3'UTR. What do you consider the 3'UTR to be? It could very well be also regulatory, like the 5'UTR.
172-173. Can you clarify if the higher expression you describe, is the added up expression of both members of a duplicated pair or are both members individually higher expressed (and do they both have more TSRs than singletons)?
178. Unlike what is written in this sentence, Fig 3f indicates that members expressed at high levels show higher tissue-specificity. Please clarify.
180. Why are the distribution pattern of TSRs an important indictor of functional conservation? Can you clarify?
184. Can you clarify the difference between antisense and bidirectional at this point in the text? If an antisense RNA initiates

within 600 bp of the canonical TSS, is this also called bidirectional? Please clarify your definitions.
198. Can you clarify in the text what you mean with the 'other' category?
205. Why is Glyma 10G142300 called an mRNA pair and not an mRNA-Prompt? Can you clarify how you make the distinction between the two categories? It seems Glyma 10G144200 produces longer RNAs than the 'prompt'of ..2300.
209. You say that genes with antisense or divergent TSRs evolved faster than the non category. The non category however seem to have evolved at similar pace as the mRNA & promts divergent categories. Please clarify your statement.
235. Please clarify why you consider the 5'UTR as regulatory while the 3'UTR is considered intragenic. I assume S10b is also about tissue-specific TSRs. If so, please mention in the legend.
238. To me figure S11 seems unnecessary. It is obvious that tissue-specific TSRs are linked to genes that play roles in tissue-specific biological roles.
221-265. In this section you look at tissue-specific TSRs, in the previous section you discussed antisense/bidirectional TSRs. I wonder why you do not indicate how many of the tissue-specific TSRs are antisense/bidirectional. It may make sense to swap (and rearrange) the two sections.
240-251. I miss a categories with antisense TSRs and bidirectional TSRs.
252-256. Group 2* and 5* were predicted to encode distinct proteins. Are there also genes within these (and the other) groups with a frameshift? How big of a difference in protein sequence would they have? And based on what feature did you extract these specific subsets of genes?
255-259. You shortly refer to GO analyses (S12 and S13), but do not come back to it and do not place it in a proper context. In other words is this analysis relevant for this paper? You may want to leave it out.
257. Reference to Fig5d is on the wrong spot in the text.
265. I would add 'potentially' before functional innovation. Or do you have clear indications there is a difference in protein functionality?
270. H3K4me1 is not a clear cut repressive mark in all organisms. In animals it marks poised enhancers. In plants it apparently recruits DNA repair proteins (Plant Cell doi: 10.1093/plcell/koae089) and plays a role in RdDM at a subset of loci (Niu et al Nat Comm 2021). Ricci et al 2019 Nat Plants, found H3K4me1 at transcribed genes, but that could be linked to the repression of transcription initiation reported by Nielsen et al (at 295 you suggest it is possitively linked to initiation, but Nielsen et al indicates the opposite).
288-289. The dip in H3 signal is also seen for the TSRs in other tissues. Can you hypothesize what this means? Are the C-TRSs that show a dip in H3 without being a C-TRS in leaves (while being a C-TSR in other tissues)?
294-297. H3K36me3 is the mark that is much better known to be linked to transcription elongation than H3K4me1. In animals/yeast it is know to be involved in hampering cryptic initiation of transcription.
298-299. I am not convinced that the presence of histone marks indicates thes ATIs are epigenetically regulated. Commonly you need a tissue-specific & general transcription factors to initiate transcription. The histone marks are part of the transcription (initiation) process (H3K4me3,H3K56ac, H3K36me3). My feedback holds for similar remarks elsewhere in the text.
324-325. Can you elablorate on what you mean with the divergence across species? You once mention common bean, but not more.
328. I miss references at the end of the sentence. In addition I wonder why you refer to a low degrees of tissue-specificity in soybean. At line 224 you mention that 62.7% of the TSRs were tissue-specific. I would not consider this low.
331. Divergence relative to what do you refer to?
353. You probably refer to another paper. Stroud et al 2013 is not about PROMPTs as far as I can see.
359. Can you elaborate in the text why ATI in UTRs would lead to loss or gain of regulatory motifs? Now a reader has to fill this in him/herself.
360. Have you investigated if ATI resulted in frameshifts? In other words do you in most cases produce a truncated protein rather than a different protein? Are there cases where different proteins are made?
379. What type of distinct patterns do you refer to?
387. What do you mean exactly with transcription specificity in the context of the sentence?
393-395. This sentence is not explicit enough and is a bit confusing to me. When talking about elongation marks, I interpret this as marks that promote elongation. Here you refer to the association between H3K4me1 and repression of transcription initiation.
398-404. there is an interplay between sequence-specific transcription factors, the transcription machinery and histone modifications. Histone modifications are not the major 'door/lock'. Histone acetylation and H3K36me3 are in addition clearly playing a positive role in (proper) transcription elongation. Sequence specificity comes from sequence-specific transcription factors.
It would be useful to spell out more of the abbreviations when used first in a legend.
Fig2. To me it is unclear what you show in the panel at the right in Figure 2g; the abundance of what is shown? and how does this graph refer to the graph on the left? ; Legend Fig 2: it would be useful to define S, N and B in the legend.
Figure 4b. Please indicate in the legend of 4b that the antisense graph is on the left and the bidirectional on the right.
Figure 4c. there are red and turquoise bars instead of blue ones. Same for fig 5c; In addition, is the antisense RNA anti-correlated with the sense expression of the gene? It seems like that for these two tissues, but is this also true for the other tissues? Would be interesting to look at and mention. In such case the level of antisense transcription may play a role in regulating the expression of the gene.
Figure 5. I miss a group with sense TSRs in one tissue and antisense TSRs in another tissue. According to figure 4 this exists.
Figure 5d which tissues are compared here? I do not understand the explanation in the legend of what is shown in d. % are out if the total number...
Fg 6. would be useful to spell out the abbreviations used in the legend, e.g. P-TSR & C-TSR, or Inr.

Suppl Fig 4. In the legend blue and orange are swapped.
Table S1. Personally I would find it more logic to keep track of the remaining % of reads relative to the number of clean reads. So the % would drop with each column to the right.
Table S2. You may want to add an extra tab that contains explanation of some of the headings. For example, is abundance expressed per million reads? What do you mean with number of support tissue (nr of replicates or nr of different type of tissues samples)? For example, how can you have reads in >2 tissues if the nr of support tissues is 2?
Tables S3, S4 The same would hold for other tables (an extra tab with explanations). For example Table S3, what does the NA mean? Not analysed? something else? What does a dominant TSR rate mean? For S4, what does Tissue number mean? Sample number?

Responses to the Reviewer Comments

Dear reviewers,

We would like to express our sincere gratitude for your thoughtful and constructive comments, which have significantly helped us improve the quality and scientific merit of our manuscript. To clarify the changes made in the revision as clearly as possible, we have highlighted major edits in the main text and provided a Response Table below, which precedes our detailed, point-by-point RESPONSES to your comments. This table summarizes changes related to all figures and tables, particularly those newly added (labeled in yellow). We hope that these highlights and the table will facilitate your evaluation process and underscore our appreciation for your commitment to improving our work. Additionally, for each response, if there are associated changes made in the manuscript text, we have cited corresponding page and line numbers based on our lined Word document. However, we would like to mention that the TPC submission requires authors to remove the line numbers from the manuscript, and the system automatically generates line numbers. However, there are slight discrepancies between the cited line numbers and those generated by the system. **Please see the manuscript copy with highlights and line numbers for accurate citations.** Given the extensive additions and modifications made to the Results section and the rewriting of the Discussion, we hope these details will help alleviate your workload.

Sincerely, Jianxin Ma and co-authors

Response Table. Summary of the changes made in/though figures and tables in the revision.

Categories	Original version	Revised version	Notes
Figure	Figures 1 and 2	Figures 1 and 2	Same
Figure	Figure 3	Figure 3	Fig 3F was replaced
Figure	Figure 5	Figure 4	Re-numbered, modified
Figure	Figure 4	Figure 5	Re-numbered
Figure	Figure 6	Figure 6	Same
Supplementary Figure	Fig S1-S7	Fig S1-S7	Same
Supplementary Figure	--	Fig S8	Newly added
Supplementary Figure	Fig S8	Fig S11	Re-numbered
Supplementary Figure	Fig S9, S10	Fig S9, S10	Same
Supplementary Figure	Fig S11-S13	--	Removed
Supplementary Figure	Fig S14-S16	Fig S12-S14	Re-numbered
Supplementary Table	Table S1-S3	Table S1-S3	Same
Supplementary Table	Table S4	Table S5	Re-numbered
Supplementary Table	Table S5	Table S6	Re-numbered
Supplementary Table	Table S6	Table S4	Re-numbered
Supplementary Table	Table S7	Table S7	Same
Supplementary Table	Table S8	Table S9	Re-numbered
Supplementary Table	--	Table S8	Newly added

Reviewer #1 (Comments for the Author):

I applaud the authors for a thorough and, in my view, much needed study of transcriptional initiation in plants.

Response #1.0: Thanks for the encouraging comments on our work.

There are a few points for consideration, however.

1) Reproducibility. From a bioinformatics perspective, it is always good to have code-based documentation of the data analysis. I would like to see this provided with the publication. A github repository, or equivalent, would do nicely. Just provide the scripts for read processing and mapping; Rscripts for the R analyses; etc., taking a user from raw data to analysis results.

Response #1.1: Thanks for the suggestion. We have now made all scripts for read processing, mapping, and R analyses available in a GitHub repository at https://github.com/Wanjie-Feng/STRIPE_seq.git, and provided this information on Page 20, Lines 581-583.

2) Robustness. Comments on and consequences of parameter selection should be discussed. How do the results change for a higher threshold of TSSs kept or for clustering to define TSRs?

Response #1.2: We have now provided detailed information on the parameters used to define TSRs using the CAGEfightR program. Initially, we normalized the TSS abundance using the TPM method and retained TSSs with TPM values greater than 1 in at least one tissue. This threshold was chosen based on our analysis, which indicated that a TPM value greater than 1 was suitable for retaining TSSs with sufficient read support (>3 reads, see the table below) in individual tissues. To assess the potential impact of varying TSS abundance thresholds on TSR width, we have now tested different TPM cutoffs (0, 1, 5, and 10) and evaluated their effects on TSR width (1, 10, 100, and 1000). Our analysis revealed that while the distribution of TSR widths do not significantly change between a cutoff of 0 and 1, a TPM threshold of 1 can reduced background noises such as large TSRs (>1000 bp), without significant influence on the distribution of TSR widths (see added/revised description on Page 4, Lines 102-109 updated Supplementary Tables S1 and S8), as well as and Page 18, Lines 515-527. Stricter criteria resulted in a reduction in the total number of TSRs, particularly when higher TSS thresholds were used, but the TPM threshold of "greater than 1" worked well for a clean background and for reliable TSR identification. We have now updated the methods section to include the rationales for parameter selection and provided a pipeline and demo R script at GitHub for readers to generate suitable TSR files using our approach in their own studies.

TPM > 1	Leaf	Stem tip	Seed	Pod	Nodule	Root	Flower	Shoot
Reads >	6	7	12	4	6	6	6	12

3) Accessibility. The data should make a valuable track on the SoyBase and Ensembl Plants genome browsers. If accepted for publication in TPC, I think this should be arranged.

Response #1.3: Thanks for the great suggestion. Given that only a small percentage (2.81%) of soybean genes in the Williams 82 reference genome had correctly annotated TSSs in SoyBase, we totally agree that it would be very valuable to the research community if our data are integrated into genome browser in SoyBase. Dr. Rex Nelson at SoyBase has generously agreed to offer this support, and his team has started the process of the integration of the data. Please see a screenshot of the latest email message from Dr. Nelson. We anticipate the browser track will be implemented during the review process. They indicated that their support is peripheral to the experiment and felt an acknowledgment of their participation and a reference to SoyBase.org would be appropriate. We have added a sentence in Acknowledgements on Page 20, Lines 592-593. Additionally, the TSS information can also be accessed via the website at http://xtlab.hzau.edu.cn/jbrowser/.

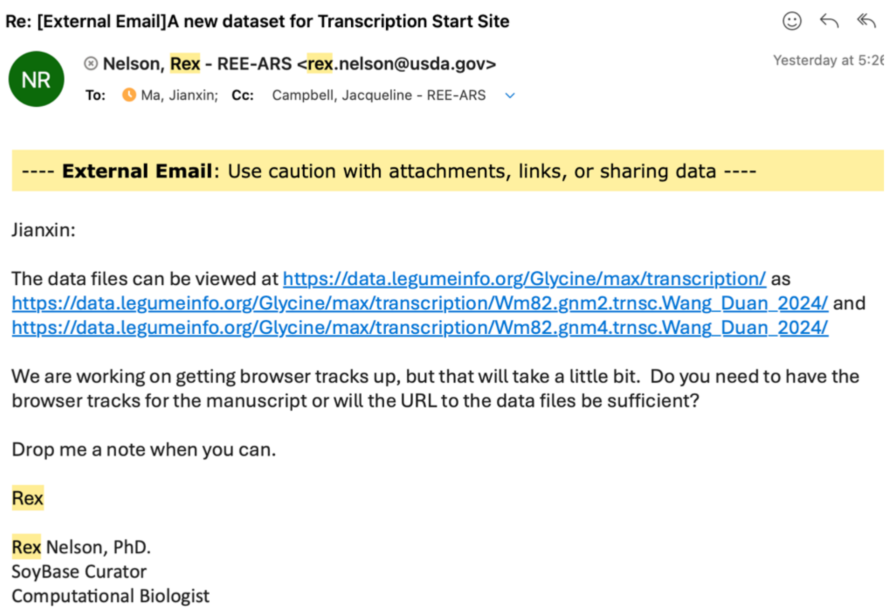
Re: [External Email]A new dataset for Transcription Start Site

NR Nelson, Rex - REE-ARS <rex.nelson@usda.gov> Yesterday at 5:26

To: Ma, Jianxin; Cc: Campbell, Jacqueline - REE-ARS

---- **External Email**: Use caution with attachments, links, or sharing data ----

Jianxin:

The data files can be viewed at https://data.legumeinfo.org/Glycine/max/transcription/ as https://data.legumeinfo.org/Glycine/max/transcription/Wm82.gnm2.trnsc.Wang_Duan_2024/ and https://data.legumeinfo.org/Glycine/max/transcription/Wm82.gnm4.trnsc.Wang_Duan_2024/

We are working on getting browser tracks up, but that will take a little bit. Do you need to have the browser tracks for the manuscript or will the URL to the data files be sufficient?

Drop me a note when you can.

Rex

Rex Nelson, PhD.
SoyBase Curator
Computational Biologist

4) Interpretation. The original STRIPE-seq publication noted a link of promoter-distal TSRs with enhancer RNAs in human samples. That publication stated " a fraction of these sites are highly enriched for H3K4me1 and H3K27ac but not H3K4me3" whereas in this study the finding is "H3K4me1, a marker of RNA polymerase II elongation, is underrepresented in TSRs within CDS." I think these associations warrant further inspection and comment.

Response #1.4: We confess that we made an inaccurate interpretation. We have now cited relevant publications and discussed diverse effects of H3K4me1 on transcription activity among organisms (see Page 12, Lines 324-333). We have particularly articulated our observations and proposed a model of ATI in CDSs (see Page 13, Lines 352-363).

Reviewer #2 (Comments for the Author):

In the submitted manuscript, Wang and colleagues describe a study on using Survey of TRanscription Initiation at Promoter Elements with high-throughput sequencing (STRIPE-seq) to annotate the Transcription Start Sites (TSSs) in the soybean reference genome. The STRIPE-seq as a technique was described in a previous publication (Policastro et al., 2020) applied to yeast and the human genome. In the manuscript it is applied to a plant genome for the first time.
Characterization of transcription initiation using high-throughput sequencing methods has been performed for plant genomes including Arabidopsis thaliana, and Zea maize. The main barrier for the adoption of regular TSSs profiling for genome annotation is that the different protocols

available are quite laborious and in need of large amounts (nanograms) of starting material, when compared to STRIPE-seq. Modifications to the method to be used in plants will be appreciated by the community.
For this study the authors generated TSSs annotations from Williams 84, the Soybean cultivar used as reference plant, from eight different tissues. Identified TSSs were clustered in regions (TSRs - groups of TSSs) and assigned to annotated genes based on distance and strand information. In addition to comparison against the reference genome the authors compared against a companion dataset of RNA-seq that was generated from the same tissues and used published ChIP-seq data from different histone marks (Lue et al., 2019) generated from leaf tissue for further contextualization of the data.

Response #2.0: Thanks for the encouraging comments on our work.

1. QC of the data, the authors presented a saturation analysis, but was only mentioned and never properly described nor discussed. Some further claims about method sensitivity, accuracy, sufficiency, etc., are mentioned often with proper description/discussion of the methods or the results. For instance, the authors evaluated accuracy, but we don't know what they found as results aren't shared with the reader, at least not explicitly.

Response #2.1: Thanks for pointing this out. We have now extended and discussed relevant QC analysis in the revised manuscript in details as advised by the reviewer and feel the added/modified description are now sufficient and easier to follow. Please see details on Pages 4-5, Lines 102-109, and Page 18, Lines 515-527.

2. The study identified TSSs for ~72% of the current annotated genes with variation across the tissues. The authors share a distribution, but how many genes in the current genome were wrongly annotated? The authors explore the data at the gene level, however the identification of multiple TSSs and TSRs should be correlated with isoforms/transcripts annotated instead of being collapsed at the gene level. At the very least the authors should include this information in the paper and focus on gene features when more relevant to the analysis.

Response #2.2: Thank you for these comments. We have now articulated that only 2.81% (1,067) of 37,911 genes have correctly annotated/predicted TSSs in the soybean reference genome. Please see Page 5, Lines 113-115. Because only 5' ends shorter than 300 base pairs were sequenced in our study, specific isoforms caused by ATIs cannot be determined by STRIPE-seq. We have now discussed this limitation and our perspectives on Page 13, Lines 371-376.

3. Characterization of the TSRs according to how spread is the transcription initiation across different TSSs for a given gene - i.e., the promoter shape. The authors found that single nucleotide (S-shaped) was the prevalent shape of TSRs in Soybean and that more than half of the genes have more than one TSR. Did you report the number of genes with more than one type of shaped TSR?

Response #2.3: Thank you for the suggestion. “the number of genes with more than one type of shaped TSRs” and relevant information has now been provided on Pages 6-7, Lines 163-167, Supplementary Table S.

4. The authors studied if different TSRs shapes were correlated with different genic features, such as expression as has been previously reported. They identified genes with S-shaped TSRs to be low expressed when contrasting the distribution, however beyond the plotting of the box-plot there is not statistically evaluation of the differences between distributions neither explanation of which dataset was used to derive the gene expression values (STRIPE-seq TPM? RNA-seq TPM?).

Response #2.4: We have now included statistics. Please see Fig. 2B and legends. In addition, we have clarified that in this study, the expression levels of TSRs were determined using STRIPE-seq TPM, which represents one of the advantages STRIPE-seq possesses compared with other approaches. Please see Page 6, Line 136 and Page 13, Lines 369-371.

5. In the discussion, the statement that claims that predominant S-shaped TSRs in the maize genome are not associated with low expressed genes is wrong. The Mejia-Guerra et al., 2015 paper specifically evaluated the correlation between shape and expression levels and found that "the median expression of genes with broad TCs is higher than the median expression of genes with sharp TCs". The figures 2C and 2D (Mejia-Guerra et al., 2015) show CAGE expression and RNA-seq expression.

Response #2.5: Thank you for pointing this out. We have now read the detailed description of the Mejia-Guerra et al., 2015 paper more carefully, and corrected our misstatement about relevant observations in maize by Mejia-Guerra et al., 2015. Please see Page 14, Lines 386-387.

6. The authors explored differences between the Ka/Ks ratio between Soybean and common bean orthologs genes across the different TSRs shapes. The authors claimed that S-shaped TSRs have undergone lower intensities of purifying selection in the Soybean genome. The hypothesis is interesting, however there is no test to support the claim that the Ka/Ks ratio is `actually different` from what is observed in genes with a different shape. Some type of measurement of the extent of this difference is missing to support the claim. There is a lot of data in plants genome duplication, exploring this hypothesis beyond Soybean using some of the TSRs profiles for other plant genomes might be beyond the scope of this project but is an interesting avenue to explore.

Response #2.6: Thanks for the valuable feedback. We have now included statistics to support our statement. In addition, we have reanalyzed the maize data reported by Mejia-Guerra et al., 2015 for identification of TSRs, calculated Ka/Ks ratios for the maize genes through comparison with their corresponding sorghum orthologs defined in our previous study as well as the gene expression data (Zhao et al. 2017), and then conducted similar analysis, including duplicated genes versus singletons, as done for soybean, and we observed similar patterns in maize (see added description on Page 6, Lines 143-156 and Page 8, Lines 209-224). Some interspecific data and observations have also been further discussed in the Discussion section (see added discussions on Pages 13-14, Lines 377-412). Together these new additions further solidified our statement in terms of evolutionary patterns of genes and their expression with distinct TSR shapes.

7. An "strikingly" observation was the identification of alternative transcription initiation overlapping with intragenic regions, in particular with annotated CDS for a large number of genes. I did not understand why this seems to be unexpected? The authors mentioned that the intragenic TSRs were more frequent than the ones located within annotated canonical promoter regions. With

what we know about the genome and transcriptions, the parsimonious hypothesis should be that the current annotation is missing a good portion of the shortest isoforms and that is why the TSRs overlap with regions other than the already annotated promoter and 5'UTRs.

Response #2.7: We intended to emphasize the fact that ATI in CDSs were largely uncharacterized or were not identified at this scale, partly due to technical challenges or costs. On the other hand, the level of "widespread" of ATI seen in soybean CDS has not been seen in other plant species yet. So, widespread ATI in CDS is not that "striking" (so the title of the manuscript has been changed); nevertheless, we now feel the different frequencies of ATI in CDS among different plant species was unexpected and deserves discussion. We have now discussed this point and provided our perspectives. Please see Pages 14-15, Lines 402-412.

8. The histone marks discussion is another half baked idea, the intention is good but the data is not sufficient to answer the question as the colocalization of the signals is not really enough for any conclusion. Some of the claimed mechanisms such as the "exhibit threshold effects on tissue-specific gene expression" don't have any support in the analysis so far.

Response #2.8: We agree with the reviewer and have toned down our statements on this point. We have articulated what are observations and what are interpretations. In addition, we have cited more relevant publications and integrated findings towards mechanistic insights into ATI in CDSs and their tissue specificity. In addition, we have removed unsupported interpretations, re-written a large portion of the Discussion section to focus on important points raised from our study. Please see revised description on Page 12, Lines 324-333 and Page 13, Lines 353-363, and newly added discussion on Pages 15-16, Lines 423-455.

9. What happened with the miRNA-seq data? It is not clear how it was used for the analysis.

Response #2.9: We generated and submitted a set of small RNA-seq data from these same tissues, but the data were designed for a different study. Sorry for the confusion, and the relevant words have been removed.

10. As a large analysis paper that relies on bioinformatics analysis of many types, code availability is missing as GEO links and description of the methods is not sufficient to ensure reproducibility.

Response #2.10: Thanks for being so mindful. We have now made all scripts for read processing, mapping, and R analyses available in a GitHub repository via the following link: https://github.com/Wanjie-Feng/STRIPE_seq.git. Please see Page 20, Lines 581-583.

Reviewer #3 (Comments for the Author):

1. This manuscript is extensively analysing alternative initiation of transcription in soybean, in different tissues. They show quite a high % of the ATI is tissue-specific and that there are relatively many TSRs in coding sequences. These seem present in narrow NFRs. The MS provides many interesting correlations. It is a pity that none of the examples is investigated functionally. Now the

data reported stays very descriptive. For example, what would the overexpression of a particular antisense RNA mean for the sense expression of a gene.

Response #3.1: We appreciate the many constructive comments from this reviewer here and below, which has helped us improve our manuscript. In response to this specific comment here, we have been working on functional characterization of a few genes producing antisense RNAs. One of those genes we'd exemplify here is *GmHEN1*, the ortholog of the Arabidopsis *Hen1* that encodes a miRNA methyltransferase. This gene was expressed in all tissues and detected to have an antisense TSS specifically expressed in nodules by STRIPE-seq. Then, the full length of the antisense transcript was obtained by 5'RACE and qRT-PCR and annotated as a putative PP2C gene, which was not annotated in the soybean reference genome. We have obtained knockout lines and overexpression lines for the antisense RNA and found it affects nodule numbers and plant productivity, but still need one more year for getting stable transgenic and gene-editing lines for more accurate phenotype evaluation and molecular dissection. This has become a project for another lab member and we feel it would be more appropriate to leave it off from this this large-scale biology article. In addition, given that functional roles of antisense RNAs in plants have been widely reported, we felt the functional significance of the antisense RNAs may not need to be further emphasized or exemplified in this article. Nevertheless, being inspired/suggested by this and another reviewer, we have added analysis of TSRs in the maize genome, which also experienced a whole genome duplication event but exhibited distinct subgenome fractionation pattern from soybean. We think the newly added comparison between these two model crops, together with other suggested changes, have provided novel evolutionary insights and substantially enhanced the quality of this manuscript.

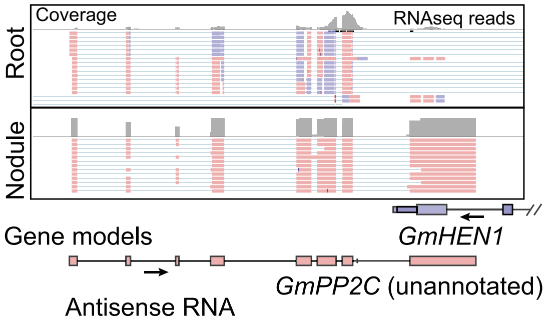

2. In the abstract they say there is little known how ATI evolves. This is however also not elaborately investigated in the paper, nor discussed. Similarly, the way ATI is regulated is not discussed. The manuscript could be improved by discussing the aspects reported in the results also in the discussion. For example, what may be the relevance of WGD genes to be higher expressed and have more TSRs?

Response #3.2: Thanks. We have now added analysis of ATI in maize using previously published CAGE data from Mejia-Guerra et al., 2015, cited our previous investigation of WGD genes versus singletons, and discussed the relevance of WGD genes to be higher expressed and have more TSRs as suggested by the reviewer. Please see the additions on Page 6, Lines 143-156; Page 8, Lines 209-224; Pages 13-14, 377-382.

3. There are quite some typos here and there. In addition, the grammar and clarity of the text needs to be improved (I did not specify the locations of typos/unclarities).

Response #3.3: Our apologies for typos and grammatical errors. We have now re-written a large portion of the manuscript and believe the clarity, logic, and coherence of the manuscript have been substantially improved.

4. Please realize that the histone modifications and H3 profile you have looked at is an integral part of gene regulation, including transcription initiation and elongation. It is not necessarily a major cause for ATI (which is suggested at various places in the text). One needs sequence-specific TFs and and/or general TFs to initiate transcription.

Response #3.4: Thanks for the advice. We have clarified the relevant statements and further discussed this important point. Please see the modified description on Pages 12-13, Lines 324-333 and 351-363, and added discussion on Page 16, Lines 442-455.

5. 3-4. The title can be improved to cover the content of the paper better. Accessible chromatin data is for example taken from another paper and only represents one of the tissues examined. So I would not place this in the title.

Response #3.5: We have modified the title and hope it more accurately reflects the key points described in this manuscript.

6. 49. you mention that canonical TSRs are located in the 5' upstream regulatory regions. I would say they are located in the core promoter. The 5' upstream regulatory regions can be located far away from the core promoter.

Response #3.6: Thanks, and suggested revision has been made through the whole manuscripts.

7. 66. Soybean is also a valuable source of protein when thinking of the foreseen/needed transition from an animal-based to a plant-based diet to mitigate the climate crisis.

Response #3.7: Suggested revision has been made. See Page 3, Line 67.

8. 79. You mention epigenomic differentiation. I wonder if there is evidence showing that within one species duplicated genes are epigenetically regulated in a different manner. If not, I would 6. leave this out. You also do not show this in this manuscript.

Response #3.8: Thanks. We have removed “epigenomic” from the sentence. See Page 4, Line 78.

9. 110. Figure 1c does not show that the nodule tissue exhibits the most unique TSRs compared with other tissue. is there another figure you can refer to?

Response #3.9: Based on the TSR expression levels, nodules exhibited the most distinct TSR expression profile from all other tissues, as reflected by the cladogram tree shown in Fig. 1C. supporting our statement that "the nodule tissue exhibits the most unique TSRs compared with other tissues". We have now articulated this in Fig. 1C legend.

10. 122-124. To me it is unclear what is shown in figure 2b if I only read the legend. According to the legend the figure shows abundance, but give there are more S shaped TSRs, I would then expect the S shaped box to be higher on the Y-axis. The main text indicates you mean expression levels (RPM?? something else?), which clarifies the figure. Please clarify the text of the legend.

Response #3.10: Sorry for the insufficient description in the legend. Figure 2B shows the relative expression levels of the three categories of genes classified based on their TSR shapes, rather than the number of TSRs in different TSR shapes. The values represent transcripts per million (TPM), which is normalized expression levels calculated using the reads from STRIPE-seq. Since each read in STRIPE-seq represents a transcript, TPM is more appropriate than RPM. We have now articulated this in the legend of Figure 2B.

11. 126-128. In Zhao et al they examine substitutions in the genes instead of the TSR, which you examine. Therefore the comparison you make is not entirely valid.

Response #3.11: Zhao et al. compared evolutionary rates of the coding sequences annotated in the reference genomes, which can be different from some isoforms from the genes produced with the different or even same TSRs, e.g., alternative splicing. Nevertheless, the similar pattern was also observed in maize as illustrated in our newly added analysis on Page 6, Lines 143-156, further supporting the credibility of the described pattern. We agree with the reviewer about the limitation of the current analysis.

12. 132-133: The text is unclear. if 80.1% contain multiple TSRs, the nr with fewer than 10 should be less than 80.1%.

Response #3.12: Our original description can easily cause confusion. We have clarified it by stating: "Of the 37,911 soybean genes with defined TSR by STRIPE-seq, 30,378 (80.1%) were found to contain multiple TSRs, either within the same tissue or across different tissues, and of these multi-TSR genes, 88.3% had fewer than ten TSRs". Please see Page 6, Lines 158-159.

13. 142-143. It would be best to refer to Fig 1a if mentioning promoter and proximal. To be honest, to me proximal would logically refer to the proximal promoter, which would be the core promoter (called promoter by you), and the region upstream of this I would define as regulatory sequences. The term Canonical TSRs I would use for TSRs at the core promoter/5'UTR. Further upstream I would not refer to as canonical.

Response #3.13: We agree with the reviewer about the definition of these terms, but we'd prefer to stick to the current definition/classification given by the CAGEfightR program that has been widely used for this type of analyses. According to this program, "proximal" refers to a promoter within 1K rather than specifically the core promoter, so to be consistent with quite a few publications in which the CAGEfightR program was used. We have also realized potential confusion by the current classification and have added "core" to our "promoters" as suggested by the reviewer through the manuscript to avoid/reduce such confusion.

14. 150-151. The intergenic regions can also contain regulatory sequences.

Response #3.14: We have modified the sentence to include this possibility. Please see Page 7, Line 178.

15. 142-150. When adding up the % (50.4; 43.5; 3.6) they do not add up to 100%, where is the about 2.5% missing TSRs?

Response #3.15: The 2.5% includes antisense (4574, 2.36%) and bidirectional (half of 506, 0.14%) TSRs. These percentages have now been added to the section "Antisense and sense TSRs exhibited similar distribution patterns along genic sequences, and so did bidirectional TSRs." Please see Page 10, Lines 272-273; Page 11, Line 291.

16. 155-158. You mention TATA box elements were barely seen around intron TSRs, while they are around antisense TSRs. Both however show a similar sequence pattern in Fig S5, with T's being more prominent in introns and A's in antisense.

Response #3.16: As shown in Fig S5, TATA-box elements were barely discernible in introns in the context of surrounding sequences that are enriched with "T", as the background. By contrast, TATA-box elements in antisense sequences were clearer contrasting with their flanking sequences. We have pointed this out in the Fig S5 legend.

17. 161. I See TA also enriched in introns (also intragenic). GG seems enriched in CDS and 3'UTR. What do you consider the 3'UTR to be? It could very well be also regulatory, like the 5'UTR.

Response #3.17: Yes, the relatively enriched dinucleotides are illustrated in Fig S6. Since few conserved regulatory elements in intragenic regions have been identified to date, we decided not to further interpret the dinucleotide patterns. In terms of 3'UTR TSRs, first, they are the least abundant, accounting for <0.5% of all TSRs identified, and second, they are thought to use different mechanisms to regulate mRNA-based processes that are different from the function of the 5'UTR, so we didn't combine this category with 5'UTR or leave it separately for pattern analysis.

18. 172-173. Can you clarify if the higher expression you describe, is the added up expression of both members of a duplicated pair or are both members individually higher expressed (and do they both have more TSRs than singletons)?

Response #3.18: We analyzed the two copies of each duplicated pair as two separate genes. More specifically, we compared genes in the duplicated status to those in the singleton status here. The values represent averages per gene in a same category. We have clarified this in the legend of Fig 3A.

19. 178. Unlike what is written in this sentence, Fig 3f indicates that members expressed at high levels show higher tissue-specificity. Please clarify.

Response #3.19: Our apologies for mistaken use of a wrong chat for Fig 3f. Now it has been replaced by the right one. The legend has also been expanded to be clearer. Nevertheless, the original writing in the sentence was based on the right dataset and thus on changes are needed. Taking this opportunity, we have also re-checked the datasets used for all other figures for accuracy and no additional errors have been found.

20. 180. Why are the distribution pattern of TSRs an important indictor of functional conservation? Can you clarify?

Response #3.20: We inappropriately used "functional conservation". Our data reveals that genes with more TSRs are less tissue specific and evolutionarily more conserved. This may be due to their fundamental significance for the organism, whereas genes with less or single TSRs are more tissue specific, and thus with more functionally specific. We have now modified the statement as: "All these observations together suggest that the distribution patterns of TSRs are important indicators of functional significance, and evolutionary conservation and divergence of duplicated genes in plant genomes." Please see Pages 8-9, Lines 225-227.

21. 184. Can you clarify the difference between antisense and bidirectional at this point in the text? If an antisense RNA initiates within 600 bp of the canonical TSS, is this also called bidirectional? Please clarify your definitions.

Response #3.21: Either antisense or bidirectional TSRs possesses opposite transcription orientation compared with the canonical reference TSRs. antisense TSRs are located downstream of canonical TSRs but within the gene, whereas bidirectional TSRs are located upstream of the reference TSRs within 600 bp from the canonical reference TSS. We have articulated the definition of antisense TSRs, now clearly distinguishing it from the bidirectional TSR category. Please see Page 10, Lines 270-271.

22. 198. Can you clarify in the text what you mean with the 'other' category?

Response #3.22: We categorized bidirectional TSRs into three distinct groups based on their relationship with the reference TSRs: 1) mRNA-mRNA: two TSRs within 600bp defining the core promoters of two adjacent genes. 2) mRNA-PROMPT: One is the canonical reference TSR for a gene and the other is located upstream of the reference TSR (so called promoter upstream transcript, PROMPT), but it doesn't seem to be part of the regulatory sequence of another gene. 3) "Other-divergence": two non-overlapping TSRs in opposite transcription orientations located downstream of the 5' UTR of a gene. We have explained a bit more about the categorization of these bidirectional TSRs. Please see Pages10-11, Lines 282-289.

23. 205. Why is Glyma 10G142300 called an mRNA pair and not an mRNA-Prompt? Can you clarify how you make the distinction between the two categories? It seems Glyma 10G144200 produces longer RNAs than the 'prompt'of ..2300.

Response #3.23: As responded in #3.22 above, the non-overlapping TSRs in opposite orientations belong to two different genes (Glyma.10G142300 and Glyma.10G142500) and were thus called an mRNA-mRNA pair.

24. 209. You say that genes with antisense or divergent TSRs evolved faster than the non category. The non category however seem to have evolved at similar pace as the mRNA & promts divergent categories. Please clarify your statement.

Response #3.24: We intended to summarize a general pattern but have now articulated specific types.

25. 235. Please clarify why you consider the 5'UTR as regulatory while the 3'UTR is considered intragenic. I assume S10b is also about tissue-specific TSRs. If so, please mention in the legend.

Response #3.25: We have responded this in "Response #3.17". We have mentioned the tissue-specific TSRs in the legend of Fig S 10B.

26. 238. To me figure S11 seems unnecessary. It is obvious that tissue-specific TSRs are linked to genes that play roles in tissue-specific biological roles.

Response #3.26: The original Fig. S11 and related GO term analysis and description have been removed.

27. 221-265. In this section you look at tissue-specific TSRs, in the previous section you discussed antisense/bidirectional TSRs. I wonder why you do not indicate how many of the tissue-specific TSRs are antisense/bidirectional. It may make sense to swap (and rearrange) the two sections.

Response #3.27: We have rearranged the two sections as suggested. Supplementary Table S5 includes the information about the proportions of tissue-specific antisense and bidirectional TSRs.

28. 240-251. I miss a categories with antisense TSRs and bidirectional TSRs.

Response #3.28: We intended to focus on the category of CDS-TSRs, as the most abundant, highly tissue-specific intragenic TSRs in this section. Antisense TSRs and bidirectional TSRs are much less abundant, so we didn't further categorize them.

29. 252-256. Group 2* and 5* were predicted to encode distinct proteins. Are there also genes within these (and the other) groups with a frameshift? How big of a difference in protein sequence would they have? And based on what feature did you extract these specific subsets of genes?

Response #3.29: Subgroups group 2* and group 5* belong to group 2 and group 5, respectively. The alternative TSRs of group 2* and group 5* are located within the coding sequences, resulting in truncated proteins or frameshift mutations that potentially affect the function of the genes based on prediction (Please see Page 10, Lines 256-257 and 264-265. We have clarified this. As we discussed, STRIPE-seq couldn't determine specific isoforms, it was difficult to define specific protein changes in most cases (Page 13, Lines 373-376).

30. 255-259. You shortly refer to GO analyses (S12 and S13), but do not come back to it and do not place it in a proper context. In other words is this analysis relevant for this paper? You may want to leave it out.

Response #3.30: As responded in #3.26, GO-term analysis description (including original S12 and S13) have been removed.

31. 257. Reference to Fig5d is on the wrong spot in the text.

Response #3.31: Sorry for the mistake. This-GO related description has been removed.

32. 265. I would add 'potentially' before functional innovation. Or do you have clear indications there is a difference in protein functionality?

Response #3.32: We have added "potentially" here as suggested. Please see Page 10, Line 265.

33. 270. H3K4me1 is not a clear cut repressive mark in all organisms. In animals it marks poised enhancers. In plants it apparently recruits DNA repair proteins (Plant Cell doi: 10.1093/plcell/koae089) and plays a role in RdDM at a subset of loci (Niu et al Nat Comm 2021). Ricci et al 2019 Nat Plants, found H3K4me1 at transcribed genes, but that could be linked to the repression of transcription initiation reported by Nielsen et al (at 295 you suggest it is possitively linked to initiation, but Nielsen et al indicates the opposite).

Response #3.33: Our apologies for the confusion caused by our misinterpretation of Fig 6B, the middle panel from the top to the bottom. Actually, our data aligned well with the findings of Nielsen et al., which suggest repressive role of H3K4me1 in intragenic transcription initiation. Our data also echo the finding from the Bae and Lesch 2020 paper, which demonstrates that while it is correlated with gene expression level, H3K4me1 is more strongly linked to a poised chromatin state, defined by the simultaneous presence of H3K4me3 and H3K27me3, than to transcriptional activity, suggesting the possible role of H3K4me1 in epigenetic memory. We have cited this paper and the ones mentioned by the reviewer (see Page 12, Lines 324-333) and articulated our observations towards a model of ATI in CDSs (see Page 13, Lines 351-363; Page 16, Lines 442-455).

34. 288-289. The dip in H3 signal is also seen for the TSRs in other tissues. Can you hypothesize what this means? Are the C-TRSs that show a dip in H3 without being a C-TRS in leaves (while being a C-TSR in other tissues)?

Response #3.34: We have now articulated our observations on Page 13, Lines 324-333, and made discussion on Page 16, Lines 442-455.

35. 294-297. H3K36me3 is the mark that is much better known to be linked to transcription elongation than H3K4me1. In animals/yeast it is know to be involved in hampering cryptic initiation of transcription.

Response #3.35: We have addressed this point in Response #3.33.

36. 298-299. I am not convinced that the presence of histone marks indicates thes ATIs are epigenetically regulated. Commonly you need a tissue-specific & general transcription factors to initiate transcription. The histone marks are part of the transcription (initiation) process (H3K4me3,H3K56ac, H3K36me3). My feedback holds for similar remarks elsewhere in the text.

Response #3.36: Also as responded in # 3.33, We agree with the reviewer on this point and have toned down our interpretations related in the Discussion section "In addition, as the H3 curve, and the active and repressive transcription-associated histone modification signals around the TSRs in CDSs in leaves are shared by other tissues in which those TSRs were even not detected, it seems clear that the relative abundances of different histone modifications and the dynamic interplays among these epigenetic modifications as illustrated in Figure 6C, as well as genetic regulators binding to the NFRs are largely responsible for tissue-specific ATI in CDSs" (see Page 16, Lines 442-455).

37. 324-325. Can you elablorate on what you mean with the divergence across species? You once mention common bean, but not more.

Response #3.37: We meant similarities and differences. We have re-written >80% of the Discussion section in this revision and tried to use the terms more accurately. For example, now we state "Some of the genomic and epigenomic features of TSRs are shared among species…"; "One of the striking observations in soybean compared with those reported in other plant species is…"; "Another contrasting observation in soybean compared with other plant species is …" etc.

38. 328. I miss references at the end of the sentence. In addition I wonder why you refer to a low degrees of tissue-specificity in soybean. At line 224 you mention that 62.7% of the TSRs were tissue-specific. I would not consider this low.

Response #3.38: Sorry for writing this incomplete sentence. We meant the TSRs in canonical regulatory regions showed relatively lower degree of tissue specificity compared with those within the intragenic regions. We have added this missing phrase. Please see Page 14, Line 397.

39. 331. Divergence relative to what do you refer to?

Response #3.39: This has been addressed as described in Response #3.37.

40. 353. You probably refer to another paper. Stroud et al 2013 is not about PROMPTs as far as I can see.

Response #3.40: Thanks for capturing this. The right reference paper was Thieffry et al. 2020. By the way, we have realized the discussion point was quite irrelevant to the focus of our study and has been removed from the Discussion section.

41. 359. Can you elaborate in the text why ATI in UTRs would lead to loss or gain of regulatory motifs? Now a reader has to fill this in him/herself.

Response #3.41: We have now elaborated the sentence and added references to support the statement: "ATI in the UTRs also can be of biological significance such as gain or loss of regulatory motifs critical for binding transcription factors and other regulatory proteins, and alternation of gene expression patterns, mRNA stability, localization, or translational efficiency (Chen and Tarn 2019; Alfonso-Gonzalez and Hilgers, 2024). Please see Page 15, Lines 434-437

42. 360. Have you investigated if ATI resulted in frameshifts? In other words do you in most cases produce a truncated protein rather than a different protein? Are there cases where different proteins are made?

Response #3.42: As we explained in Response #3.29, STRIPLE-seq has its technical limitations and in most cases cannot define protein truncation or frameshift, so added “potentially” to reflect the possibilities. Please see Page 15, Line 424.

43. 379. What type of distinct patterns do you refer to?

Response #3.43: As rephrased on Page 14, Lines 398-400, “the genomic and epigenomic features of TSRs situated within the canonical regulatory regions of genes such as the presence of TATA-box motifs, and relatively broader TSRs, longer NFRs, and lower degrees of tissue specificity compared with those within the intragenic regions”.

44. 387. What do you mean exactly with transcription specificity in the context of the sentence?

Response #3.44: We meant 'tissue specificity. The original sentence has been deleted, and we have now mentioned “tissue-specific” on Page 16, Line 454.

45. 393-395. This sentence is not explicit enough and is a bit confusing to me. When talking about elongation marks, I interpret this as marks that promote elongation. Here you refer to the association between H3K4me1 and repression of transcription initiation.

Response #3.45: The relevant paragraphs in both Results and Discission sections have been substantially modified or re-written, and this sentence has been removed. We think our description of the potential role of H3K4me1 and our interpterion are clearer now (Please see Page 12, Lines 324-333).

46. 398-404. there is an interplay between sequence-specific transcription factors, the transcription machinery and histone modifications. Histone modifications are not the major 'door/lock'. Histone acetylation and H3K36me3 are in addition clearly playing a positive role in (proper) transcription elongation. Sequence specificity comes from sequence-specific transcription factors.

Response #3.46: Thanks. We confess that our interpretation here was not well supported by either our data or published work, so we have re-written the discussion on this point on Page 16, Lines 442-455.

47. It would be useful to spell out more of the abbreviations when used first in a legend.

Response #3.47: Thank you for your suggestion. We have revised the manuscript to ensure that all abbreviations are spelled out in full upon their first use in a legend.

48. Fig2. To me it is unclear what you show in the panel at the right in Figure 2g; the abundance of what is shown? and how does this graph refer to the graph on the left? ; Legend Fig 2: it would be useful to define S, N and B in the legend.

Response #3.48: The right panel of Figure 2G presents the abundance of TSRs, normalized by STRIPE-seq reads (TPM), illustrating the distribution of TSR abundances across different gene features. The left panel shows the proportion of three TSR shapes (broad, narrow, and single-base TSRs) within each gene feature. We have updated the figure legend to explain these more clearly.

49. Figure 4b. Please indicate in the legend of 4b that the antisense graph is on the left and the bidirectional on the right.

Response #3.49: Thank you for your suggestion. We have updated the figure 4b (i.e., Fig 5B in this revised version) and its legend in the revised manuscript.

50. Figure 4c. there are red and turquoise bars instead of blue ones. Same for fig 5c; In addition, is the antisense RNA anti-correlated with the sense expression of the gene? It seems like that for these two tissues, but is this also true for the other tissues? Would be interesting to look at and mention. In such case the level of antisense transcription may play a role in regulating the expression of the gene.

Response #3.50: Thanks for the insightful comments. We have corrected the description about the bar colors in the legends to accurately represent transcriptional directions: red bars now indicate sense transcription (left to right), and green bars represent antisense transcription (right to left). We have also conducted comparison between antisense-TSRs with sense TSRs across multiple tissues, and observed an overall negative correlation as shown in newly added Fig. 5F. In addition, we have mentioned the regulatory role of antisense transcription as the reviewer mentioned. Please see Page 11, Lines 311-312.

51. Figure 5. I miss a group with sense TSRs in one tissue and antisense TSRs in another tissue. According to figure 4 this exists.

Response #3.51: Yes, this exists. Nevertheless, we intended not to further categorize these TSRs here, given that the small number of this particular group of TSRs generally cannot reveal a clear pattern.

52. Figure 5d which tissues are compared here? I do not understand the explanation in the legend of what is shown in d. % are out if the total number...

Response #3.52: The percentages represent the proportions of genes in each category when comparing every possible combination of two tissues among the eight tissues studied. Each percentage reflects one of 28 possible pairwise tissue comparisons. Additionally, we clarified that while percentages for groups 1-6 are calculated based on the total number of genes, the percentages for groups 2* and 5* are calculated relative to the total numbers of genes in groups 2 and 5, respectively. We have now described these details in the legend.

53. Fg 6. would be useful to spell out the abbreviations used in the legend, e.g. P-TSR & C-TSR, or lnr.

Response #3.53: We have now spelled out the abbreviations when first shown in a legend.

54. Suppl Fig 4. In the legend blue and orange are swapped.

Response #3.54: We apologize for the carelessness. Correction has been made.

55. Table S1. Personally I would find it more logic to keep track of the remaining % of reads relative to the number of clean reads. So the % would drop with each column to the right.

Response #3.55: We have updated the table as suggested.

56. Table S2. You may want to add an extra tab that contains explanation of some of the headings. For example, is abundance expressed per million reads? What do you mean with number of support tissue (nr of replicates or nr of different type of tissues samples)? For example, how can you have reads in >2 tissues if the nr of support tissues is 2?

Response #3.56: We have revised the column annotations in the table to provide more detailed descriptions as suggested.

57. Tables S3, S4 The same would hold for other tables (an extra tab with explanations). For example Table S3, what does the NA mean? Not analysed? something else? What does a dominant TSR rate mean? For S4, what does Tissue number mean? Sample number?

Response #3.57: Suggested modifications have been made.

We would like to extend our heartfelt gratitude to this reviewer for this set of comprehensive and constructive comments, which have significantly enhanced the quality of our manuscript.

Dr. Jianxin Ma
Purdue University
West Lafayette, Indiana 47907

Dear Jianxin:

We have received two reviews of your revised manuscript entitled "Non-canonical Alternative Transcription Initiation is Prominently Tissue-Specific, Evolutionarily Shaped, and Epigenetically Tuned in Paleopolyploid Plants." One of the reviewers evaluated your initial submission, and this reviewer acknowledges the excellent job done in addressing all the concerns raised.

Unfortunately, the other two reviewers that evaluated your initial submission were not available, hence we sought the comments of a new reviewer.

As you will see below, this reviewer raised a concern that is central to one of the main conclusions of the study, and that is to what extent the internal TSS that STRIPE-seq identified represent artifacts of the technique, rather that true TSSs. I find this a valid concern that unfortunately can only be addressed with some additional experimentation. Unbiased experimental validation of a subset of TSSs (including some ATIs in CDSs) by primer extension or RACE-PCR could be one way to address this major concern, although you might be able to find another orthogonal method to validate these potentially interesting findings. In any case, it is indispensable that the concern of the reviewer is addressed before we can consider this manuscript further.

Please contact us if there are ambiguous comments or if you wish to discuss the revision.

Given the nature of the comments, we are offering you 60 days from when we have issued this decision to complete the revision. If a revision is not returned within this time frame, and if you have not been granted an extension, we will withdraw the manuscript, which will leave you free to submit the work elsewhere. If you need an extension, we encourage you to contact us at any point before the 60 days have passed.

When you are ready to submit the revised version, please upload a highlighted copy that indicates all changes made in response to the editor and reviewer recommendations. Include an itemized list of all changes made in response to each of the reviewer's suggestions in the "Response to Reviewers" section; please note that reviewers do not have access to your cover letter, nor was this decision letter shared with them.

Thank you for the privilege of reviewing your work. We look forward to receiving your revised manuscript.

Sincerely,

The Plant Cell Editorial Board

(Please note that due to a potential conflict of interest, EIC Blake Meyers did not participate in evaluating this manuscript).

Please note the following:

-The Plant Cell now requires authors to complete and submit an author revisions checklist upon submission of a revised manuscript. The aim of the checklist is to aid authors in preparing a high-quality manuscript, facilitate the review and assessment of revised manuscripts, and help to ensure that journal standards are maintained across the board. If your manuscript is accepted, the completed checklist will be published as supplemental material attached to the article online. Please download a copy of the checklist (pdf fillable form) at this link, for submission with your revised manuscript: https://tpc.msubmit.net/html/Author_Revisions_Checklist.pdf.

-Supplemental materials should be restricted to large datasets and tables, presentation of replicates, and validation of reagents, methods, or genotypes. Any data that are used to support the major claims must be in the main manuscript. Supplemental figure legends must indicate what figure in the main manuscript is supported by the supplemental data presented. Please justify how each of the supplemental figures meet the criteria.

-Sampling methods and nature of "biological replicates" should be described precisely (i.e. different plants, parts of plants, pooled tissue, independent pools of tissue, sampled at different times, etc), along with a clear description of and rationale for any statistical analyses conducted. The reader should know exactly what was sampled; what forms the basis of the calculation of any means and statistical parameters reported. This is also necessary to ensure that proper statistical analysis was conducted.

-Want to add this revision deadline to your calendar? Click below!

-- Reviewer comments:

Reviewer #1 (Comments for the Author):

You have done a great job with the revision, and I have no doubt this will be a well-received paper that will stimulate others in the community to follow up.

Reviewer #4 (Comments for the Author):

I will clarify that I was not a reviewer of the initial submission of this manuscript. I do think that the authors largely did provide a clear set of responses to the majority of the concerned raised by the first round of reviewers that address specific points. However, in some cases the key fundamental concerns of the reviewers were not necessarily addressed.

Major concerns:
That being said, I initially simply read the manuscript and considered the evidence fresh. I have a fundamental concern about the data used for this manuscript. I know it can be frustrating to have new issues raised in the second evaluation of the manuscript but I feel that there is a critical issue not raised in the initial reviews.

Fundamentally, I am not convinced that the methodology used in this manuscript is identifying TSRs robustly. My hypothesis is as follows: A substantial proportion of the "TSRs" identified by STRIPE-seq represent incomplete digestion of products rather than true capped started sites. This could represent partial digestion due to primary or secondary features of the mRNAs that result in some undigested remnant transcripts being sequenced using this method. Indeed - the methods section of the manuscript states that "to REDUCE the proportion of uncapped RNA...:" which implies this is a reduction of the uncapped molecules rather than a full removal. If this were true it would account for many of the observations in this manuscript as a technical issue rather than providing novel biological insights. If my hypothesis is true it would suggest that additional TSRs / TSSs would (1) often be present within the transcribed region downstream of the true TSS, (2) be enriched in genes that are highly expressed as they would simply represent a sampling issue, (3) that novel TSRs within coding regions would often be lower expressed as they would simply represent a small subset of the transcripts from any locus and (4) that internal (C-TSRs) would have chromatin patterns similar to coding regions rather than promoters.

As far as I can tell from the presented data all of these are true. The TSRs are highly enriched 3' of the annotated TSR position with very few being 5' of the annotated TSR - which would be expected if these were largely revealing partially digested transcripts. It was hard to see a really clear comparison of the relative expression level of the P=TSR and the C-TSR for the same gene. However, bulk analyses of TSRs suggest that S-TSRs are much lower expressed (Figure 1B) and these are much more common when present within the expected transcript. (Figure 1G). The fact that these are much more lowly expressed highly confounds the evolutionary analysis as lower expressed genes often exhibit higher Ka/Ks which is what is observed for S shaped TSRs. It is not clear if it is the shape that is associated with the altered Ka/Ks or simply the expression level. I would also argue that any gene with fewer STRIPE-seq reads is more likely to get classified as S-shaped due to fewer reads, not necessarily because there is only a simple start site but just due to sampling of reads from the TSR.

I will clarify that I am not suggesting that my concern would account for all findings in this manuscript. However, without evidence that the majority of the internal TSRs represent actual capped transcripts I would have a difficult time trusting the other reported findings and interpretations in this work. I would want to see validation of these transcripts using a secondary approach (such as CAGE or other approaches) that provides robust evidence that many of these represent actual transcriptional start sites rather than potential artifacts.

RESPONSES TO REVIEWERS' COMMENTS

Reviewer #1 (Comments for the Author):
You have done a great job with the revision, and I have no doubt this will be a well-received paper that will stimulate others in the community to follow up.

Reviewer #4 (Comments for the Author):
I will clarify that I was not a reviewer of the initial submission of this manuscript. I do think that the authors largely did provide a clear set of responses to the majority of the concerned raised by the first round of reviewers that address specific points. However, in some cases the key fundamental concerns of the reviewers were not necessarily addressed.

Major concerns:

That being said, I initially simply read the manuscript and considered the evidence fresh. I have a fundamental concern about the data used for this manuscript. I know it can be frustrating to have new issues raised in the second evaluation of the manuscript but I feel that there is a critical issue not raised in the initial reviews.

Fundamentally, I am not convinced that the methodology used in this manuscript is identifying TSRs robustly. My hypothesis is as follows: A substantial proportion of the "TSRs" identified by STRIPE-seq represent incomplete digestion of products rather than true capped started sites. This could represent partial digestion due to primary or secondary features of the mRNAs that result in some undigested remnant transcripts being sequenced using this method. Indeed - the methods section of the manuscript states that "to REDUCE the proportion of uncapped RNA...:" which implies this is a reduction of the uncapped molecules rather than a full removal. If this were true it would account for many of the observations in this manuscript as a technical issue rather than providing novel biological insights. If my hypothesis is true it would suggest that additional TSRs / TSSs would (1) often be present within the transcribed region downstream of the true TSS, (2) be enriched in genes that are highly expressed as they would simply represent a sampling issue, (3) that novel TSRs within coding regions would often be lower expressed as they would simply represent a small subset of the transcripts from any locus and (4) that internal (C-TSRs) would have chromatin patterns similar to coding regions rather than promoters.

RESPONSE: We are very grateful to the reviewer for providing a set of thoughtful comments. These comments have helped us realize a real issue with the last version of our manuscript: our insufficient description of the STRIPE-seq method and some criteria used for processing our data, rather than the reliability of the data itself. Although the method was previously described by the developer Policastro et al. (2020, *Genome Research*), we should have also described it with enough details to enable readers to comprehend its capability in identifying capped transcripts. That being said, we completely agree with the reviewer that the TEX digestion cannot remove uncapped transcripts entirely; thus, we used the word "REDUCE" to reflect this fact. To address this issue, the STRIPE-seq library construction involves two additional enzymatic steps after the TEX treatment: template-switching reverse transcription (TSRT) and library PCR. These steps help deplete uncapped RNA from total RNA, as illustrated in the **Response Fig. 1** on page 4 (cited from Policastro et al. 2020). TSRT is performed using a barcoded reverse transcription oligo (RTO)

primed with a random pentamer, followed by the addition of a unique molecular identifier (UMI)-containing, 5′-biotin-modified template-switching oligo (TSO) with three 3′ riboguanosines that permit the annealing of the oligo to the untemplated triplet Cs that are generated by reverse transcriptase when it reached the m7G cap. Library PCR was then performed using the cleaned TSRT product as input, ensuring that TruSeq adapters were present on both sides of the insert (blue line). The cap-adjacent base, which identifies the transcription start site (TSS), was identified using the signature sequence [N8]-TATAGGG in the R1 read from the 5' end (**Response Fig. 1**).

As indicated by our observation that, on average, ~92% reads contain a unique molecular identifier (UMI) in each tissue, even these three enzymatic steps could not fully remove uncapped transcripts. Therefore, we excluded all reads without UMI. In addition, all reads from a same site with a same UMI were considered as products from PCR in library construction and were combined and counted as a single unique read, and the copy number of this type of unique reads with different UMIs was used to evaluate relative abundance of each TSS. Furthermore, we filtered the data with criteria that required a TSS to have transcripts per million (TPM) > 1 in at least one tissue, resulting in an average unique read count of 7.3 per tissue, this filtering process would effectively remove TSSs from degraded RNAs.

We have now added these details of the method to the INTRODUCTION session, as highlighted in yellow on **Pages 3 and 4**, and articulated the criteria used in our analysis, as highlighted in yellow on **Pages 5 and 6**, in the revised manuscript.

As far as I can tell from the presented data all of these are true. The TSRs are highly enriched 3' of the annotated TSR position with very few being 5' of the annotated TSR - which would be expected if these were largely revealing partially digested transcripts. It was hard to see a really clear comparison of the relative expression level of the P=TSR and the C-TSR for the same gene. However, bulk analyses of TSRs suggest that S-TSRs are much lower expressed (Figure 1B) and these are much more common when present within the expected transcript. (Figure 1G). The fact that these are much more lowly expressed highly confounds the evolutionary analysis as lower expressed genes often exhibit higher Ka/Ks which is what is observed for S shaped TSRs. It is not clear if it is the shape that is associated with the altered Ka/Ks or simply the expression level. I would also argue that any gene with fewer STRIPE-seq reads is more likely to get classified as S-shaped due to fewer reads, not necessarily because there is only a simple start site but just due to sampling of reads from the TSR.

RESPONSE: We believe the reviewer assumed that annotated TSSs in the soybean reference genome are mostly correct, however, the opposite is true: most were wrongly annotated without any experimental evidence. This judgment is based on the fact that the annotated TSS positions in current soybean reference genome do not show a clear signature of either typical TATA-box or the Pyrimidine-Purine (PyPu) motifs in the Initiator (Inr) elements (Supplementary Fig. 2). In contrast, as we stated in the manuscript, "only the TSRs defined by STRIPE-seq showed typical TATA-box and Pyrimidine-Purine (PyPu) motifs in the Initiator (Inr) elements (Supplementary Fig. S2), which were enriched at +30 bp and +1 bp upstream of the identified TSRs, respectively (Fig. 1D)" (See newly added and highlighted sentences on **Page 6**. This difference between annotated TSSs and identified by STRIPE-seq just reveals how poorly the TSSs in the reference genome were annotated, rather than evidence of partial digestion of transcripts.

For an individual gene, more reads could potentially reveal additional TSSs and the TSR shapes may also be slightly different. In fact, even if we simply change the cutoff of the TSS

counts from the cutoff of >7 unique reads in each tissue with different UMIs to a lower number such as 6, some TSR shapes could be slightly changed. However, this would also affect even highly expressed genes, many of which contain individual TSSs vary drastically in relative abundance, as shown in the **Response Fig. 2** (on page 5), which exemplifies 10 randomly selected genes with CDS-TSRs. As our goal was to define a set of reliable TSRs and to reveal biological insights, we decided to use a strict cutoff—a TSS was called when unique reads meet TPM > 1 in at least one tissue. The pattern of lower expression of genes showing higher Ka/Ks, as revealed by unique reads with UMIs from the STRIPE-seq data is consistent to that revealed using RNA-seq data, further solidifying our observations in this study. Furthermore, similar associations between TSR shapes as identified by CAGE, gene expression levels, and Ka/Ks values were also observed maize, suggesting the authenticity of such patterns in plants. Regarding the TSR shapes, for sure they can be slightly changed with more or less sequencing data and with different cutoffs for calling a single TSS, but the general pattern would not be changed, as illustrated in the maize study (Mejía-Guerra et al. 2015), which only defined TSR shapes into two categories: narrow and broad and also observed an association between the shapes and gene expression levels.

Additionally, we'd like to highlight our detected association between open chromatin regions revealed by distribution of epigenetic marks from an independent study (Lu et al. 2019) and CDS-TSRs detected by STRIPE-seq in this study, which would have not been detected if the identified TSSs in our study were from degraded RNA fragments.

I will clarify that I am not suggesting that my concern would account for all findings in this manuscript. However, without evidence that the majority of the internal TSRs represent actual capped transcripts I would have a difficult time trusting the other reported findings and interpretations in this work. I would want to see validation of these transcripts using a secondary approach (such as CAGE or other approaches) that provides robust evidence that many of these represent actual transcriptional start sites rather than potential artifacts.

RESPONSES: We hope that, with the added details in the description of the method and data analysis as described above, the reviewer is convinced that our data identified a set of genuine TSRs including internal ones, and these updates in the manuscript will also potentially erase a similar concern from future audience.

In case the reviewer still has concerns about our data, we would like to share that we have been conducting two follow-up research projects with this set of data: The first is functional characterization of a few CDS-TSR transcripts. Over a year ago, we selected a few genes showing nodule-specific CDS-TSRs and did 5'RACE-PCR to validate the CDS-TSRs detected by STRIPE-seq—all were validated (**Response Fig. 3** on page 6). We have obtained stable transgenic lines overexpressing CDS-ATI-induced transcripts and are in the process of conducting further molecular and phenotypic analyses. The second is comparative analysis of TSRs and ATIs detected in cultivated soybean (demonstrated in this study) and a separate set of STRIPE-seq data more recently generated from eight tissues of a wild soybean accession, as exemplified in **Response Fig. 2**—snapshots of our ongoing analyses. Most of the CDS-TSRs detected in the two genomes are shared, demonstrating their replicability.

Once again, we'd express our appreciation to the reviewer for the opportunity to enhance the level of the clarity and robustness of our data and analysis.

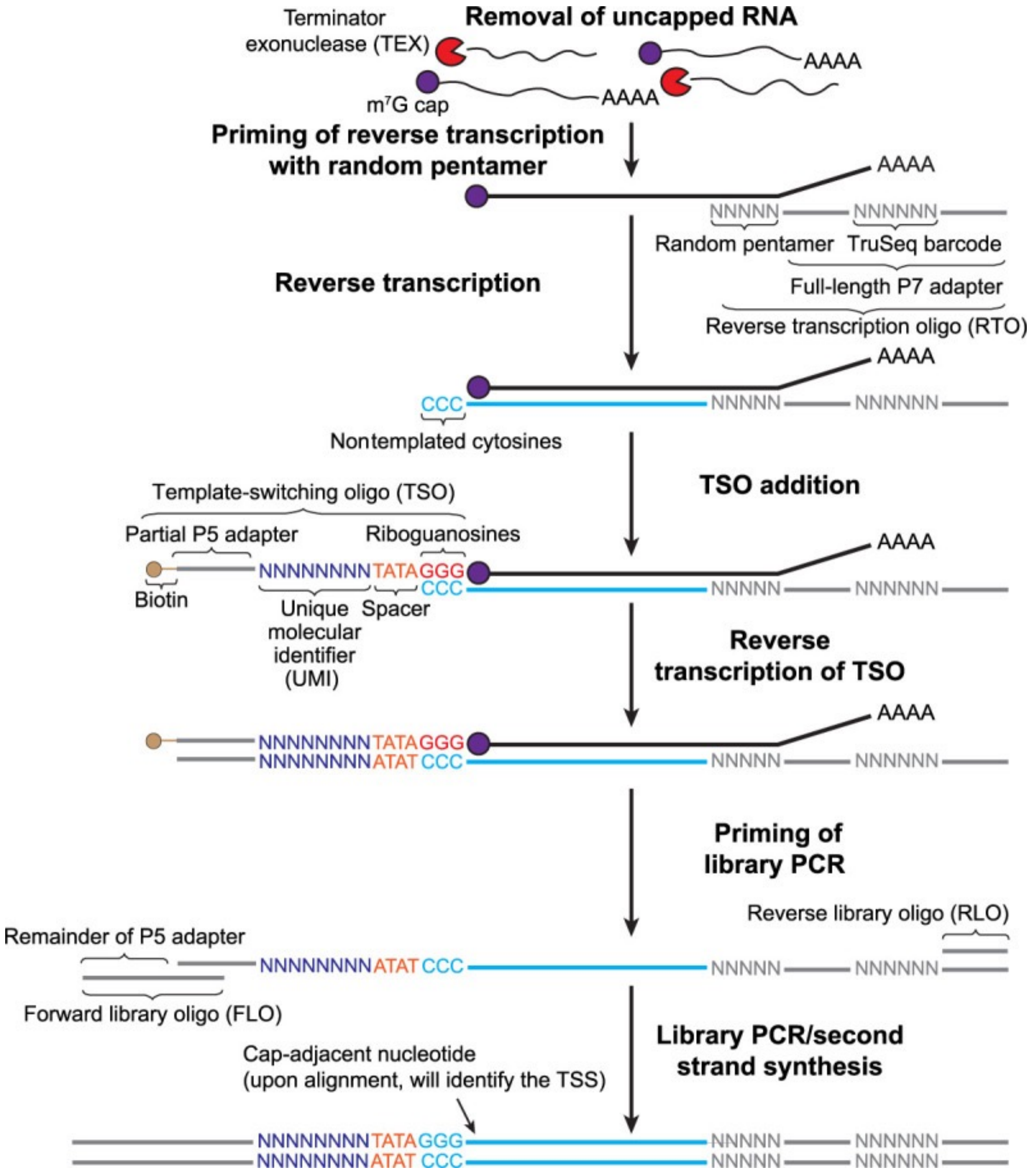

Response Fig. 1. STRIPE-seq library construction involves three enzymatic steps to deplete uncapped transcripts.

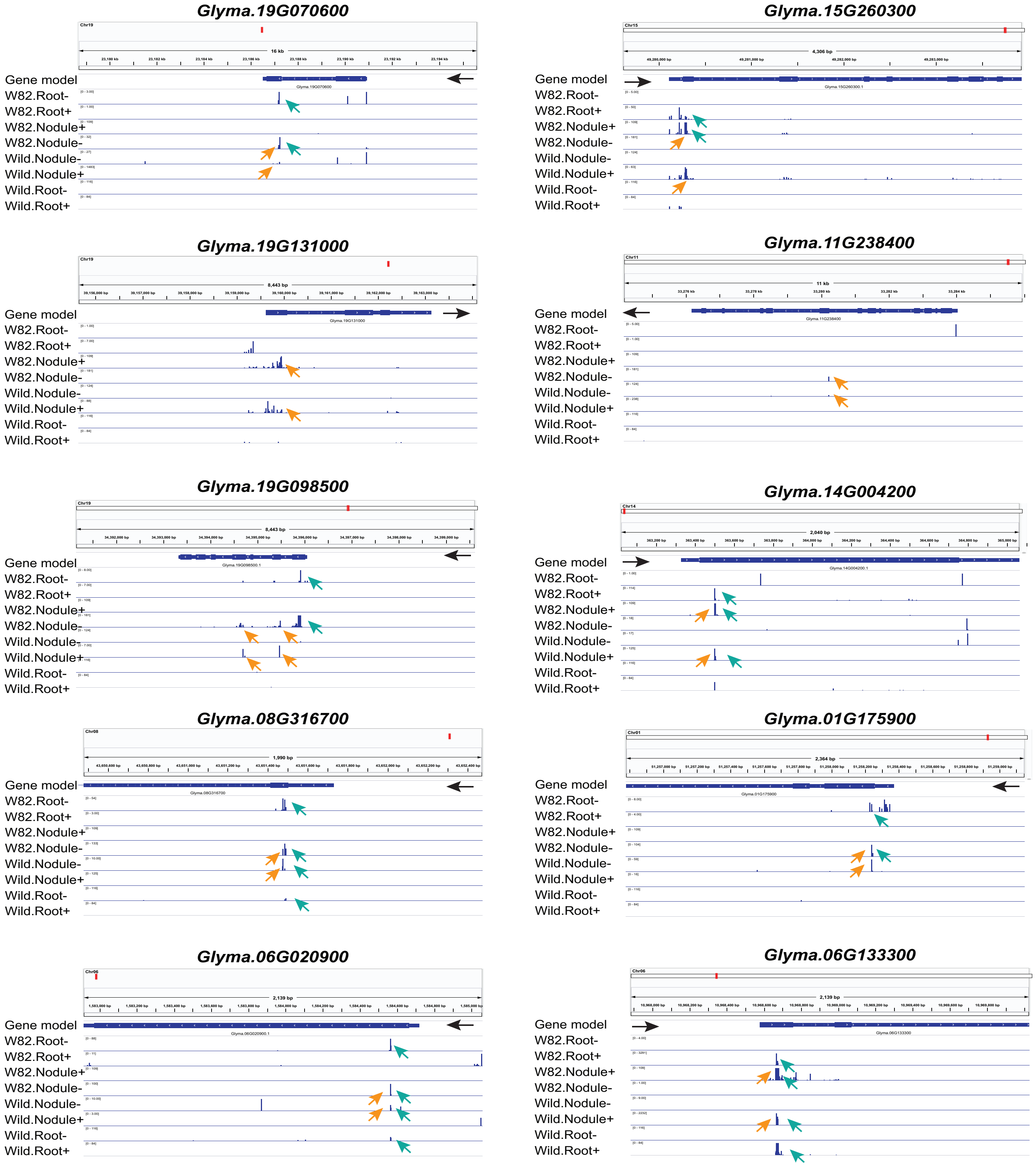

Response Fig . 2. TSS distribution and abundance of TSSs of randomly selected soybean gene expressed in root and/or nodule with CDS-TSRs. Wild, a wild soybean; W82, cultivated soybean Williams 82 used for the reference genome. Orange arrows indicate TSSs shared between roots and nodules of Williams 82; Green arrows indicate TSSs shared between roots and nodules of the wild soybean accession. Blue bars represent gene structures with thicker portions as exons; Black arrows indicate transcription orientations. Numbers within brackets indicate ranges of copy numbers of all TSSs identified in individual genes.

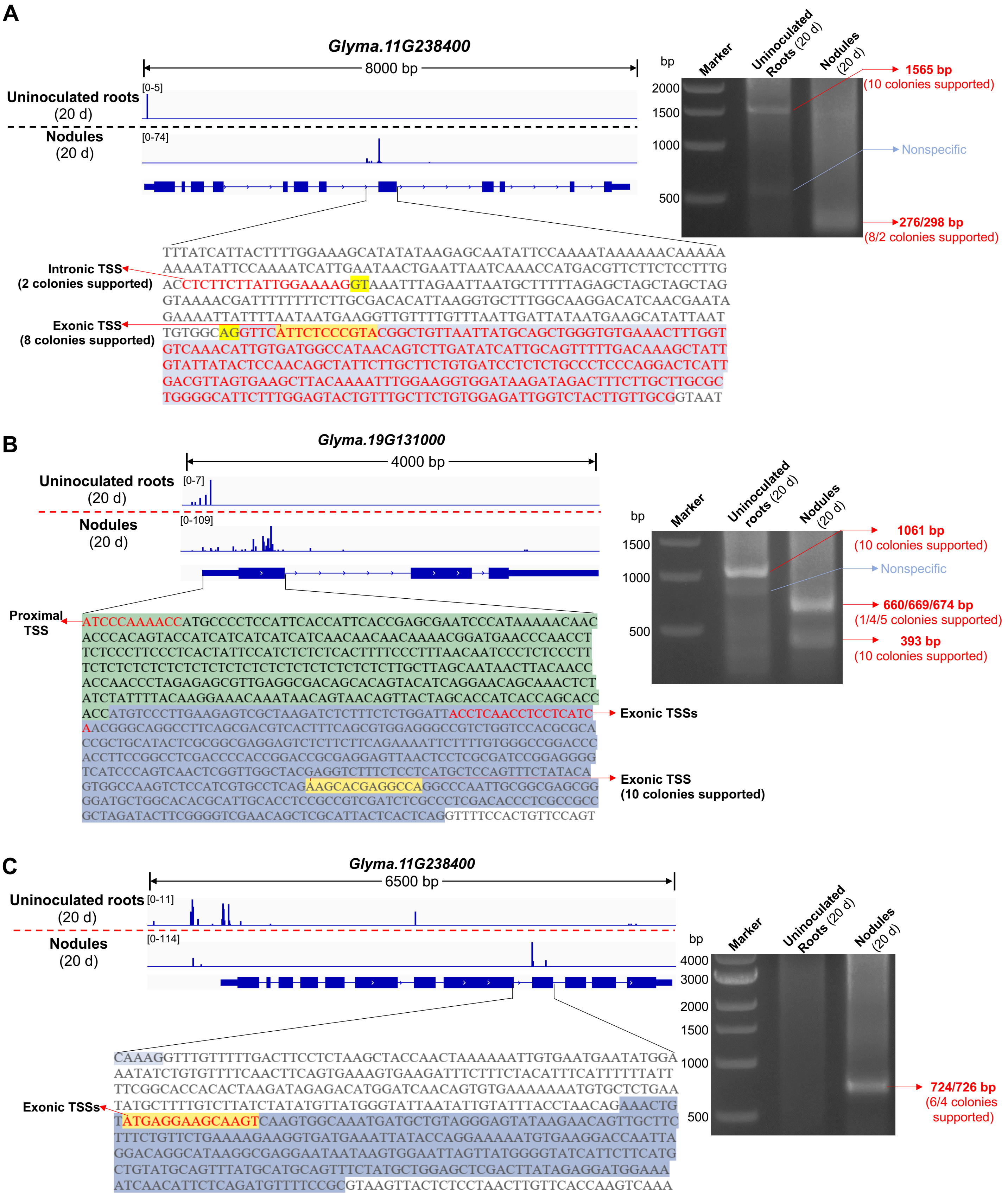

Response Fig 3. 5' rapid amplification of cDNA end (RACE) sequencing validated CDS-TSRs from three soybean genes chosen for functional characterization. RACE PCR fragments (shown on agarose gels) were cloned into a plasmid and transformed into E. coli, and subsequently sequenced by Sanger approach.

Dear Jianxin:

We have received a review of your revised manuscript entitled "Non-canonical Alternative Transcription Initiation is Prominently Tissue-Specific, Evolutionarily Shaped, and Epigenetically Tuned in Paleopolyploid Plants." On the basis of the advice received, the board of reviewing editors would like to accept your manuscript for publication in The Plant Cell. This acceptance is contingent on revision based on the comments of the reviewer. The reviewer is requesting a few more simple analyses that would best go as supplementary figures that would add confidence to the results.

Please highlight all changes and include a detailed annotation of changes of the text, with line numbers, and noting your responses to the comments.

To submit your revised manuscript, click:

Link Not Available

If you have any questions about the revision submission procedures, please contact the Editorial Office Staff (tpc-submissions@aspb.org). If you cannot return the revised manuscript within 30 days, please let us know. Otherwise, we will assume that you have elected not to revise the manuscript and withdraw it.

Thank you very much for the privilege of reviewing this work. I look forward to receiving the next version.

Sincerely,

The Plant Cell Editorial Board

(Please note that due to a potential conflict of interest, EIC Blake Meyers did not participate in evaluating this manuscript).

Reviewer #5 (Comments for the Author):

The authors provide additional detail in their response to my previous review that is helpful. I appreciate the response figure 1 provided by the authors to clarify the additional steps. I do agree that these steps will all reduce the chance of making false TSR assignments for transcripts that are partially reverse transcribed prior to a pause. I also agree that having a requirement of >1TPM reduces false-positive calls.

The authors responses to some of the questions / assertions I previously made regarding the internal TSRs addressed some questions I have. One primary concern that I did not voice well is the potential effect of expression level on several of the findings. If we assume that the method and filtering the authors have done is robust to remove false-positive TSRs there are still some important biological questions related to expression levels and whether some of the reported findings are driven by TSRs patterns (single vs multiple) or location (C-TSRs vs P-TSRs) or whether there is confounding with expression levels. I have some suggested minor analyses that could add to supplemental figures to help the reader understand the reported findings. I think these are useful for understanding the biological relevance of these findings and should be relatively simple analyses for the authors to incorporate as supplemental figures and do not require any additional data generation.

1. For the finding that there is stronger purifying selection for genes with multiple TSRs relative to genes with a single TSR: If you simply compare genes with higher expression to genes with lower expression do you see the same pattern? I am trying to understand whether having multiple TSRs is driving this change or if having multiple TSRs is more common for higher expression changes. Which factor is most important in being associated with purifying expression - higher expression or multiple TSRs?
2. I am interested in understanding the relative expression level of P-TSRs and C-TSRs in the same tissue for specific genes. Could the authors add a supplemental figure that plots the distribution of log2 (P-TSRs / C-TSRs) per gene for each tissue? I am curious to understand if the relative level of expression from P-TSRs and C-TSRs for the same gene are roughly similar or if the P-TSRs contribute the bulk of the expression (and how often are C-TSRs higher expressed than P-TSRs for the same gene in a tissue). This is an important question to understand as it may relate to a number of the findings. The authors report that C-TSRs do not show similar TATA-box enrichments or chromatin patterns. This could reflect a distinct mechanism for initiation or it could reflect lower expression from these C-TSRs.
3. For the analyses of chromatin patterns at P-TSRs and C-TSRs. What do these plots look like if the authors plot each of the 4 quartiles of expression levels for both P-TSRs and C-TSRs? In other words, do the most highly expressed C-TSRs have patterns similar to low-expressed P-TSRs are are they still distinct? This would be very useful in showing that the location (P-TSRs vs Co-TSRs) is more critical in determining chromatin enrichments than the expression level.

Please also note the following:

-The Plant Cell now requires authors to complete and submit an author revisions checklist upon submission of a revised manuscript. The aim of the checklist is to aid authors in preparing a high-quality manuscript, facilitate the review and assessment of revised manuscripts, and help to ensure that journal standards are maintained across the board. If your manuscript is accepted, the completed checklist will be published as supplemental material attached to the article online. Please download a copy of the checklist (pdf fillable form) at this link, for submission with your revised manuscript: https://tpc.msubmit.net/html/Author_Revisions_Checklist.pdf.

-Supplemental materials should be restricted to large datasets and tables, presentation of replicates, and validation of reagents, methods, or genotypes. Any data that are used to support the major claims must be in the main manuscript. Supplemental figure legends must indicate what figure in the main manuscript is supported by the supplemental data presented. Please justify how each of the supplemental figures meet the criteria.

-Sampling methods and the nature of "biological replicates" should be described precisely (i.e. different plants, parts of plants, pooled tissue, independent pools of tissue, sampled at different times, etc.), along with a clear description of and rationale for any statistical analyses conducted. The reader should know exactly what was sampled; what forms the basis of the calculation of any means and statistical parameters reported. This is also necessary to ensure that proper statistical analysis was conducted.

RESPONSES TO REVIEWERS' COMMENTS

Reviewer #5 (Comments for the Author):

The authors provide additional detail in their response to my previous review that is helpful. I appreciate the response figure 1 provided by the authors to clarify the additional steps. I do agree that these steps will all reduce the chance of making false TSR assignments for transcripts that are partially reverse transcribed prior to a pause. I also agree that having a requirement of >1TPM reduces false-positive calls.

RESPONSE: Thanks for the notes. We are happy to have addressed the previous comments provided by the reviewer.

The authors responses to some of the questions / assertions I previously made regarding the internal TSRs addressed some questions I have. One primary concern that I did not voice well is the potential effect of expression level on several of the findings. If we assume that the method and filtering the authors have done is robust to remove false-positive TSRs there are still some important biological questions related to expression levels and whether some of the reported findings are driven by TSRs patterns (single vs multiple) or location (C-TSRs vs P-TSRs) or whether there is confounding with expression levels. I have some suggested minor analyses that could add to supplemental figures to help the reader understand the reported findings. I think these are useful for understanding the biological relevance of these findings and should be relatively simple analyses for the authors to incorporate as supplemental figures and do not require any additional data generation.

RESPONSE: We appreciated additional comments and suggestions. We have done all additional analyses suggested by the reviewer, as detailed below.

1. For the finding that there is stronger purifying selection for genes with multiple TSRs relative to genes with a single TSR: If you simply compare genes with higher expression to genes with lower expression do you see the same pattern? I am trying to understand whether having multiple TSRs is driving this change or if having multiple TSRs is more common for higher expression changes. Which factor is most important in being associated with purifying expression - higher expression or multiple TSRs?

RESPONSE: Following the suggestion #1, we have conducted comparison between genes with multiple TSRs versus genes with single TSRs and observed an overall lower Ka/Ks for genes with multiple TSRs that that for genes with a single TSR (see newly added **Supplementary Fig. 5A**). When the genes are categorized into four subgroups based on the quantiles of gene expression levels from the lowest 25% (q1) to highest 25% (q4), an overall lower Ka/Ks for genes with multiple TSRs that that for genes with a single TSR within each subgroup/quantile of expression levels are still detected (see newly added **Supplementary Fig. 5B**). Nevertheless, TSR numbers and gene expression levels are correlated, with correlation coeffects ranging from 0.54 to 0.60 (see newly added **Supplementary Fig. 5C**), it remains unclear which factor is more important in being associated with purifying expression. We have added this analysis and briefly discussed this point (see highlighted text on **pages 6-7**).

2. I am interested in understanding the relative expression level of P-TSRs and C-TSRs in the same tissue for specific genes. Could the authors add a supplemental figure that plots the distribution of log2 (P-TSRs / C-TSRs) per gene for each tissue? I am curious to understand if the relative level of expression from P-TSRs and C-TSRs for the same gene are roughly similar or if the P-TSRs contribute the bulk of the expression (and how often are C-TSRs higher expressed than P-TSRs for the same gene in a tissue). This is an important question to understand as it may relate to a number of the findings. The authors report that C-TSRs do not show similar TATA-box enrichments or chromatin patterns. This could reflect a distinct mechanism for initiation or it could reflect lower expression from these C-TSRs.

RESPONSE: Following the suggestion #2, we have newly added **Supplementary Fig. S8** that plots the distribution of log2 (P-TSRs / C-TSRs) per gene for each tissue. As shown in this supplementary figure, the majority of P-TSRs showed higher expression than C-TSRs. Nevertheless, 4.50-9.84% of C-TSRs showed higher expression that P-TSRs for the same genes. Our previously described observations together with additional observations, may reflect a distinct mechanism for transcription initiation in coding sequences. We have added this analysis and briefly discussed this point (see highlighted text on **page 8**).

3. For the analyses of chromatin patterns at P-TSRs and C-TSRs. What do these plots look like if the authors plot each of the 4 quartiles of expression levels for both P-TSRs and C-TSRs? In other words, do the most highly expressed C-TSRs have patterns similar to low-expressed P-TSRs are are they still distinct? This would be very useful in showing that the location (P-TSRs vs Co-TSRs) is more critical in determining chromatin enrichments than the expression level.

RESPONSE: Following the suggestion #3, we have plotted each of the 4 quartiles of expression levels for P-TSRs and C-TSRs. As shown in newly added **Supplementary Fig. S8**, similar patterns have been seen among the 4 quartiles. These observations may suggest that the location (P-TSRs vs Co-TSRs) is more critical in determining chromatin enrichments than the expression level. We have added this analysis and briefly discussed this point (see highlighted text on **pages 13-14**).

Once again, we'd express our appreciation to the reviewer for this additional opportunity to enhance the level of the clarity and robustness of our data and analysis.

Dear Jianxin:

We are pleased to inform you that your paper entitled "Non-canonical Alternative Transcription Initiation is Prominently Tissue-Specific, Evolutionarily Shaped, and Epigenetically Tuned in Paleopolyploid Plants" has been accepted for publication in The Plant Cell, pending a final editorial review by a science editor. At this stage, your manuscript will be evaluated by a science editor with respect to its presentation of scientific content, compliance with journal policies, and presentation for a broad readership. The Plant Cell has contracted with Plant Editors (planteditors.com) to provide this service to our authors, and you will soon receive additional information on this process.

Please note that each author needs to link their ORCID identifier to their account in the system before your manuscript can be published. If any authors do not have an ORCID linked to their account, they will receive a message with a link to complete this task. Please ensure that ALL of your coauthors have completed this task as soon as possible.

LICENSE INFORMATION Shortly after your paper is sent to production, you will receive an email from our publisher's author support team, **SciPris**, with information on article processing charges, as well as the types of Open Access license you would be able to purchase. The accepted version of your manuscript will not appear on our Advance Articles page until the license is selected and signed, so please look out for an email from SciPris. For more information see the Article Publication Fees section of our author guidelines: https://academic.oup.com/plcell/pages/General-Instructions

The Plant Cell and The Arabidopsis Information Resource (TAIR) are collaborating to collect functional annotation data about Arabidopsis genes from authors. This includes information about the gene's molecular function (e.g., kinase activity, ATP synthetase activity), the biological process/es it is involved in (e.g., endosperm development, threonine biosynthesis), its subcellular location (e.g., nucleus, ER), anatomical or developmental expression pattern (e.g., leaf, ovule, flower stage 10, seedling stage), or its partner in a protein-protein interaction (e.g., AT1G01010 interacts with AT1G01020).

If your paper contains results falling into one or more of these categories for Arabidopsis genes, we request that you now submit these data for inclusion in TAIR by filling in the form provided at the following URL: https://www.arabidopsis.org/doc/submit/functional_annotation/123. If you need further clarification on what types of data can be submitted please contact curator@arabidopsis.org.

Finally, we encourage your submission of artwork for the journal cover. If there is an image or illustration related to your paper that you would like to have considered, please email it in .pdf or .tif format to clowe@aspb.org. An image title, brief, 2-3 sentence description of the image, and the name of the person credited for the image are also encouraged. If your image is selected to appear on the online cover of the journal, you will be contacted with the scheduled issue date and further information.

We look forward to seeing your paper published.

Sincerely,

The Plant Cell Editorial Board

(Please note that due to a potential conflict of interest, EIC Blake Meyers did not participate in evaluating this manuscript).

=====
IMPORTANT REMINDER: PEER REVIEW REPORTS
=====

If you opted to publish a peer review report along with your article during the original submission process, it will be prepared by the editorial staff and publicly posted with your manuscript, inside the zip file that contains any other supplemental material. As a reminder, the peer review report is a public record of all comments from editors and reviewers, as well as your prior responses, as you received them in the decision letters for each draft of your manuscript. If you agreed to publish this report and have changed your mind, or are not sure if you selected this option, please contact the editorial office as soon as possible before signing the license agreement from our publisher.

----FOR ASPB OFFICE USE ONLY (DO NOT EDIT)----
MSID: 40866
Corresponding Author: Dr. Ma
